# Discovery of PROTACs recruiting the E3 ligase CHIP

**DOI:** 10.1039/d6cb00124f

**Published:** 2026-09-21

**Authors:** Tokiha Masuda-Ozawa, Shusuke Tomoshige, Eisuke Hayakawa, Shinichi Sato, Kenji Ohgane, Fumiaki Ohtake, Minoru Ishikawa

**Affiliations:** a Graduate School of Life Sciences, Tohoku University 2-1-1 Katahira, Aoba-ku Sendai 980-8577 Japan; b Frontier Research Institute for Interdisciplinary Sciences, Tohoku University 6-3 Aramaki aza-Aoba, Aoba-ku Sendai 980-8578 Japan; c Department of Chemistry, Ochanomizu University 2-1-1 Otsuka, Bunkyo-ku Tokyo 112-8610 Japan; d Laboratory of Protein Degradation, Institute for Advanced Life Sciences, Hoshi University 2-4-41 Ebara, Shinagawa-ku Tokyo Japan; e Graduate School of Pharmacy and Pharmaceutical Sciences, Hoshi University 2-4-41 Ebara, Shinagawa-ku Tokyo Japan

## Abstract

Proteolysis-targeting chimeras (PROTACs) are promising degraders in targeted protein degradation (TPD) systems. Although more than 600 E3 ubiquitin (Ub) ligases exist in the human genome, only a few E3 ligases have been engaged in PROTACs. It is essential to expand the range of available E3 ligase resources to combat drug resistance caused by their mutations and to find suitable candidates for the degradation of certain target proteins. In this study, we aimed to develop novel PROTACs utilizing the carboxyl terminus of the Hsc70-interacting protein (CHIP) E3 ligase, which was previously unutilized. We designed and synthesized PROTACs conjugated with CHIP ligands and target protein ligands *via* linkers. These PROTACs form a ternary complex with CHIP and a target protein and ubiquitylate the target protein *in vitro*. Furthermore, taking advantage of the fact that CHIP is not an intricate multi-subunit E3 ligase, unlike other E3 ligases utilized for PROTACs, an *in vitro* ubiquitylation assay revealed that CHIP induced not only the lysine (K) 48-linked polyubiquitin (poly-Ub) chain, which is a typical proteasome degradation signal, but also the K63 linkage. Furthermore, it induces the proteasome-dependent degradation of HaloTag protein and endogenous bromodomain-containing protein 4 (BRD4). Thus, we successfully expanded the E3 ligase toolbox for PROTACs. These new PROTACs using CHIP, which induce non-canonical proteasome degradation signals containing a mixed linkage of poly-Ub chains on the target protein, can be applied to the degradation of other proteins, especially disease-related proteins.

## Abstract 2

To expand the range of available E3 ligase resources, we developed novel proteolysis targeting chimeras (PROTACs) utilizing the carboxyl terminus of the Hsc70-interacting protein (CHIP) E3 ligase, which was previously unutilized.

## Graphical abstract

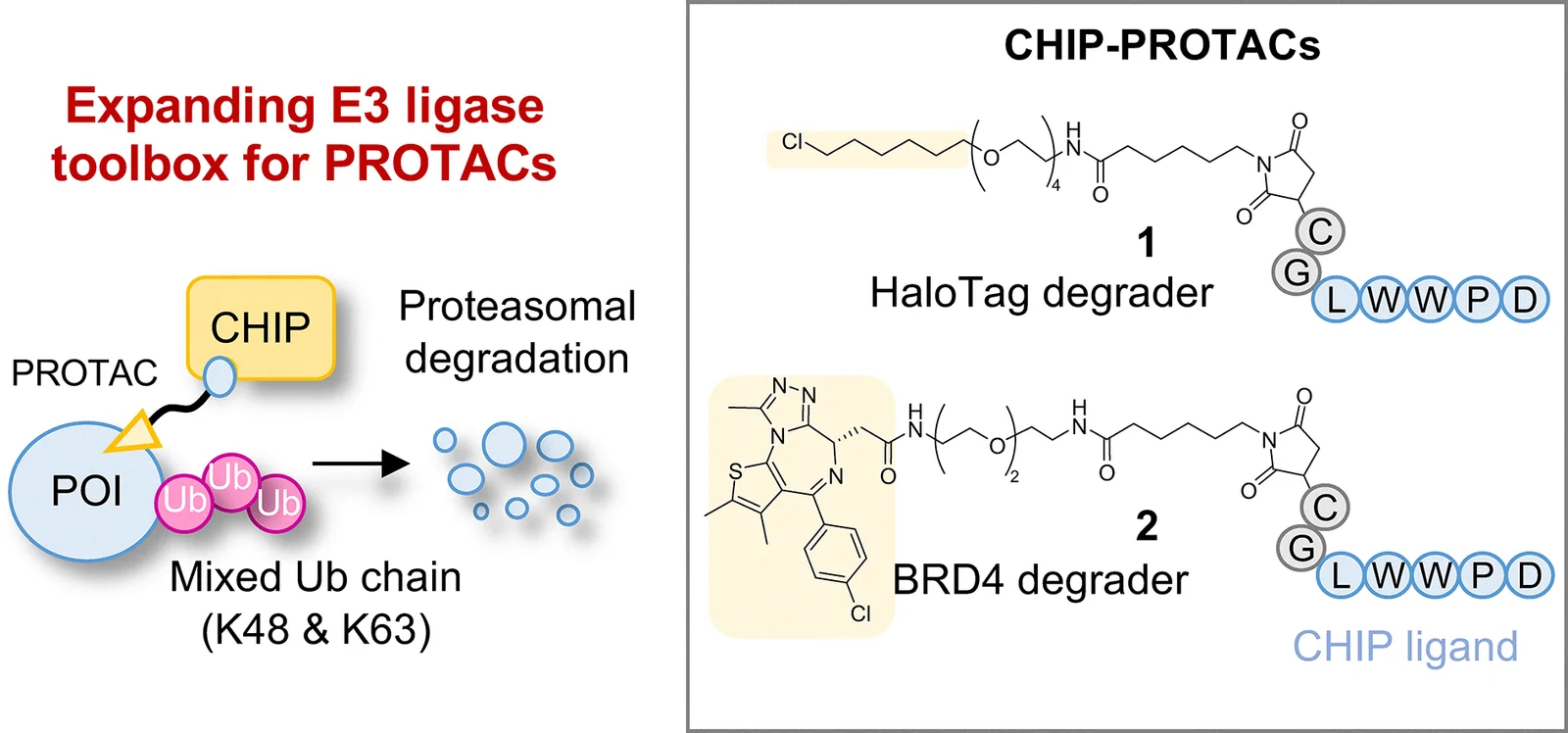

## Introduction

Targeted protein degradation (TPD) is a newly developed strategy that reduces target protein abundance without genetic manipulation. This approach is capable of removing unwanted proteins, especially undruggable therapeutic target proteins (*e.g.* transcription factors, scaffold proteins, proteins without active pockets, and protein aggregates), *via* conventional small-molecule drugs.^1^ Proteolysis-targeting chimeras (PROTACs), the most prominent degrader in the TPD strategy, are heterobifunctional molecules composed of two ligands linked by a linker that brings an E3 ubiquitin (Ub) ligase in close proximity to the target protein.^2–4^ Target proteins are ubiquitylated by the recruited E3 ligase and are then degraded in the proteasome. Several PROTAC-type drugs are currently in clinical trials, including vepdegestrant (ARV-471) and BMS-986365 (CC-94676) for estrogen receptor-positive/human epidermal growth factor receptor 2 (HER2)-negative breast cancer and metastatic castration-resistant prostate cancer, respectively.^5^ Meanwhile, it has been shown that chronic exposure to cereblon (CRBN)- and von Hippel-Lindau (VHL)-based PROTACs in tumor cell lines can cause drug resistance.^6^ In the case of immunomodulatory drugs (IMiDs), the mechanism of resistance in multiple myeloma is not fully understood. However, this resistance is being overcome by next-generation IMiDs that feature improved binding affinity to CRBN and increased potency against the same targets.^7^ The mechanism of resistance to PROTAC has been shown to be rooted in the acquisition of a genetic mutation in a hotspot of the protein–protein interaction (PPI) surface, followed by the alteration of ternary-complex formation activity.^7,8^ To overcome resistance caused by such alteration of PPI between E3 ligases and PROTACs, a potent method using alternative E3 ligases has been developed. For instance, DCAF1-based PROTAC degrades target proteins in cells with acquired drug resistance following treatment with PROTACs.^9^ In addition, one E3 ligase showed better degradation of the target protein than the other E3 ligases.^10^ The proposed mechanisms for E3-dependent target degradation efficiency changes involve either the formation of a degradation-competent ternary complex structure in the E3-PROTAC-target protein or dependence on the subcellular localization of E3 ligase. Accordingly, expanding the E3 ligase resources is desirable in the field of PROTAC.^11^ Currently, only a few E3 ligases, such as CRBN,^12^ VHL,^13^ inhibitors of apoptosis proteins (IAPs),^4^ and mouse double-minute 2 homolog (MDM2),^14^ have achieved great success in PROTACs, whereas there are more than 600 E3 ligases in the human genome.^15^ Recently, several new E3 ligases have been reported for use in PROTACs.^16^ In addition to DCAF1,^9^ DCAF16,^17^ TRIM21,^18^ and KLHDC2^19–21^ have been identified as E3 ligases in PROTACs. In addition to drug resistance, CRBN and VHL are the most commonly used substrate recognition subunit of the multi-subunit complex of Cullin RING ligases (CRLs), consisting of a scaffold Cullin protein.^22^ The regulation of the functional and structural integrity of CRLs may be challenging. Within the E3 family, E3 ligases that function as single proteins—unlike CRLs, which operate as multi-subunit complexes—are readily regulated and are therefore suitable for elucidating the biological mechanisms of PROTAC-induced target protein degradation. Therefore, we focused on the U-box type E3 ligase CHIP, which is encoded as *STUB1* (STIP1 homology and U-box containing protein 1) gene.^23^ The reported crystal structure and biochemical data have shown that CHIP is an asymmetric homodimer 35 kDa protein containing an N-terminal tetratricopeptide repeat (TPR) domain, a helical hairpin (HH) domain, and a C-terminal U-box.^24^ The interaction between the C-termini of heat shock cognate 70 (Hsc70), Hsp70, and Hsp90 chaperones is facilitated by the TPR domain.^23,25^ The U-box domain facilitates interactions with various E2 enzymes and conjugation of the poly-Ub chain to target proteins.^26,27^ It has been suggested that CHIP is responsible for the quality control of misfolded chaperone client proteins^28,29^ by switching their fate from chaperone-mediated refolding to Ub-proteasome system (UPS)-mediated degradation *via* lysine 48 (K48)-linked polyubiquitylation.^30,31^ In addition to K48-linked poly-Ub, CHIP can mediate other lysine-linked poly-Ubs (K6, K11, K27, K29, K33, and K63) on targets, chaperones, and CHIP itself.^26,32,33^ Numerous target proteins of the CHIP E3 ligase have been reported, including oncogenes, tumor suppressors, and neurodegenerative disease-related proteins (*e.g.*, tumor protein 53 (p53),^34–37^ HER2,^38,39^ immature breakpoint cluster region gene-Abelson murine leukemia viral oncogene (BCR-ABL),^40^ tau,^41–45^ alpha-synuclein,^46,47^ and NOS^48,49^).^50^ In addition, CHIP is ubiquitously expressed across almost all tissue types and localized within various cellular compartments. It is particularly highly expressed in skeletal muscle, the heart, pancreas, and brain, which exhibit high metabolic activity and rapid protein turnover.^23,51^ These features are suitable for PROTACs and applicable to a wide range of targets. To date, only a few ligands, binders, and inhibitors of CHIP have been developed and their affinities are relatively weak.^52–56^ Recently, the N-terminal acetylated pentapeptide Ac-LWWPD (named CHIPOpt) was discovered through comprehensive amino acid sequence screening of the C-terminal pentapeptides of Hsp70 and Hsp90 (M/IEEVD) that interact with the TPR domain of CHIP. Fluorescein isothiocyanate (FITC)-labeled CHIPOpt (FITC-Ahx-LWWPD) has a significantly higher binding affinity (apparent p*K*_d_ was 7.8 ± 0.1) than Hsp70s (FITC-Ahx-IEEVD; p*K*_d_ of 6.8 ± 0.1) or Hsp90s (FITC-Ahx-MEEVD; p*K*_d_ of 6.1 ± 0.1).^57^ Early proof-of-concept (POC) for PROTACs relied on peptidyl ligands, such as those for β-TRCP, VHL, and KEAP1.^2,58–60^ In particular, the application of VHL, a most widely utilized E3 ligase in the field, began with the Crews group's use of a peptide ligand.^58^ Subsequent research led to the development of small-molecule ligands with a strong binding affinity, ultimately enabling the synthesis of PROTACs with high specificity and degradation efficiency. However, peptide ligands are generally less suitable than small molecules regarding pharmacokinetics, even small-molecule recruitment does not always guarantee success; for instance, the PROTACs using small DCAF15-binder did not achieve target degradation through the recruitment of DCAF15.^59,60^ Thus, we chose the LWWPD pentapeptide sequence, a promising CHIP ligand, as the CHIP-recruiting moiety.

In this study, we aimed to expand the range of E3 ligases for PROTACs and developed a PROTAC that utilizes the CHIP E3 ligase. We synthesized chimeric compounds linking the peptide ligand of CHIP to small-molecule ligands for the target proteins (1 and 2, Fig. 1) and confirmed that these compounds could form a ternary complex with CHIP and the target proteins and further induce ubiquitylation *in vitro*. Interestingly, the poly-Ub chain in the target protein was a mixture of K48 and K63 linkages. It is possible that these mixed Ub chains enhance the proteasomal degradation signal^61,62^ and/or induce multiple degradation pathways.^63,64^ We also examined whether these PROTACs could degrade target proteins, such as the cytosolic tag protein HaloTag and the tumor-related protein endogenous bromodomain-containing protein 4 (BRD4), and verified the degradation pathways. Our results expand the PROTAC design strategy and the degradable proteome of PROTACs. This study also provides an option for overcoming PROTAC resistance.

**Fig. 1:**
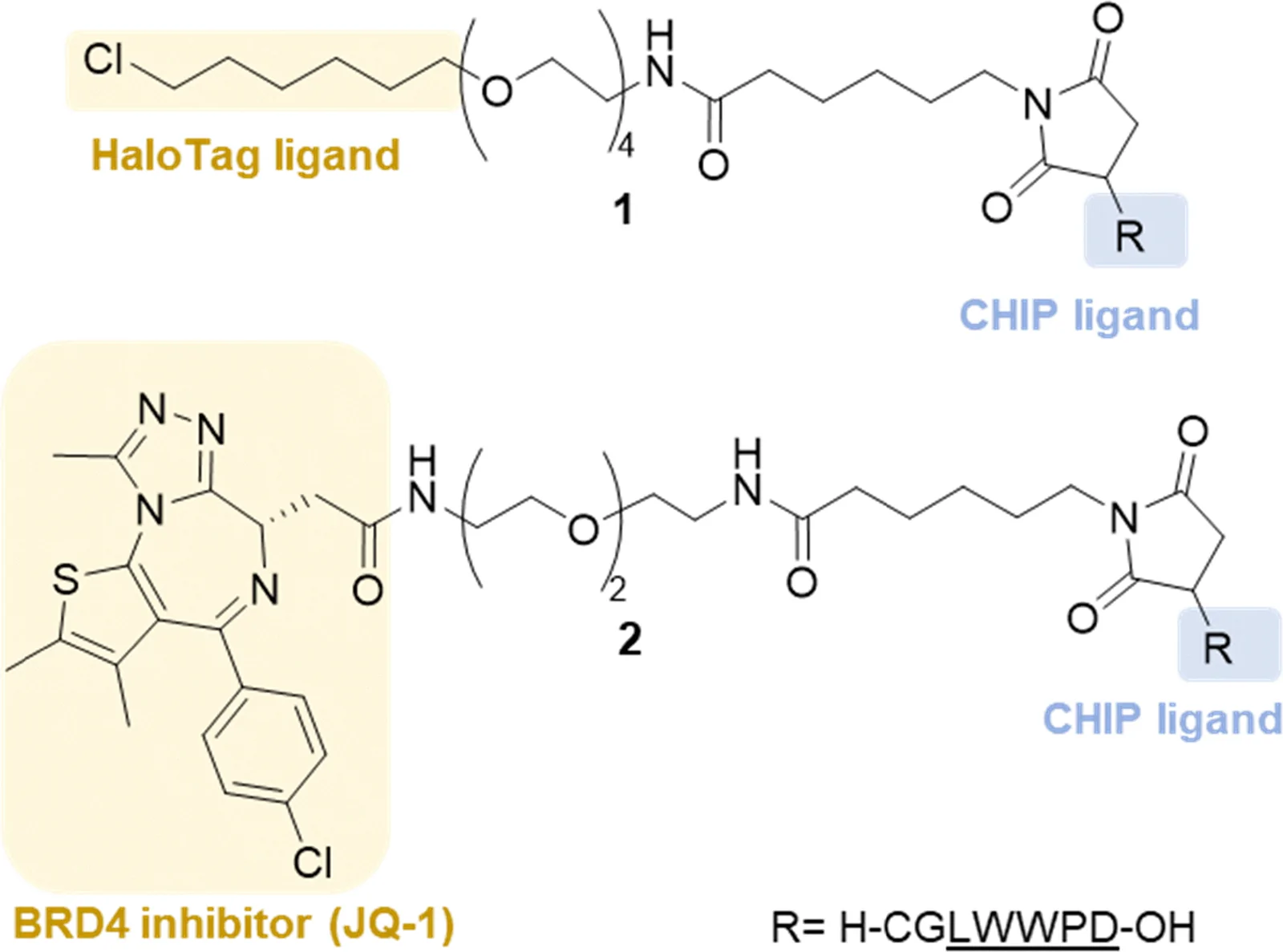
Chemical structures of degraders for the HaloTag and BRD4 proteins. The degraders 1 and 2 are the hetero-bifunctional molecules, containing the target protein ligand and CHIP E3 ligase-binding peptide. The CHIP-binding pentapeptide sequence was underlined.

## Results and discussion

We chose the pentapeptide sequence LWWPD as the CHIP ligand. To evaluate whether CHIP E3 ligase is PROTACable, we employed HaloTag as a protein of interest (POI), which has previously been targeted by PROTACs.^3,65,66^ HaloTag is a self-labeling tag that covalently binds to chloroalkane ligands. Chloroalkanes bind to Asp (D) 106 in the active pocket of HaloTag.^67^ We designed a heterobifunctional molecule, 1, which targets the HaloTag protein (Fig. 1). HaloTag and E3 ligase ligands were conjugated with a linker bearing PEG_4_. On the CHIP ligand, the C-terminal D of LWWPD is the key residue for interaction with CHIP;^57^ thus, we left the C-terminal region and added Cys-Gly (CG) residues on the N-terminus to conjugate the PEG linker by Michael addition (Scheme 1). Triethylene glycol (3) was monotosylated *via* TsCl treatment to obtain 4. The resulting tosylate was substituted with an azide in the S_N_2 reaction, followed by attachment of the chloroalkane moiety in the second S_N_2 reaction using 1-chloro-6-iodohexane. The resulting compound 7 was hydrogenated to convert the azide into an amine, which was then coupled with succinimidyl ester 9 to yield intermediate 10. Finally, the LWWPD CHIP-binding peptide was conjugated by Michael addition *via* the cysteine thiol of CG attached at the N-terminus of LWWPD to obtain the target molecule 1.

**Scheme 1:**
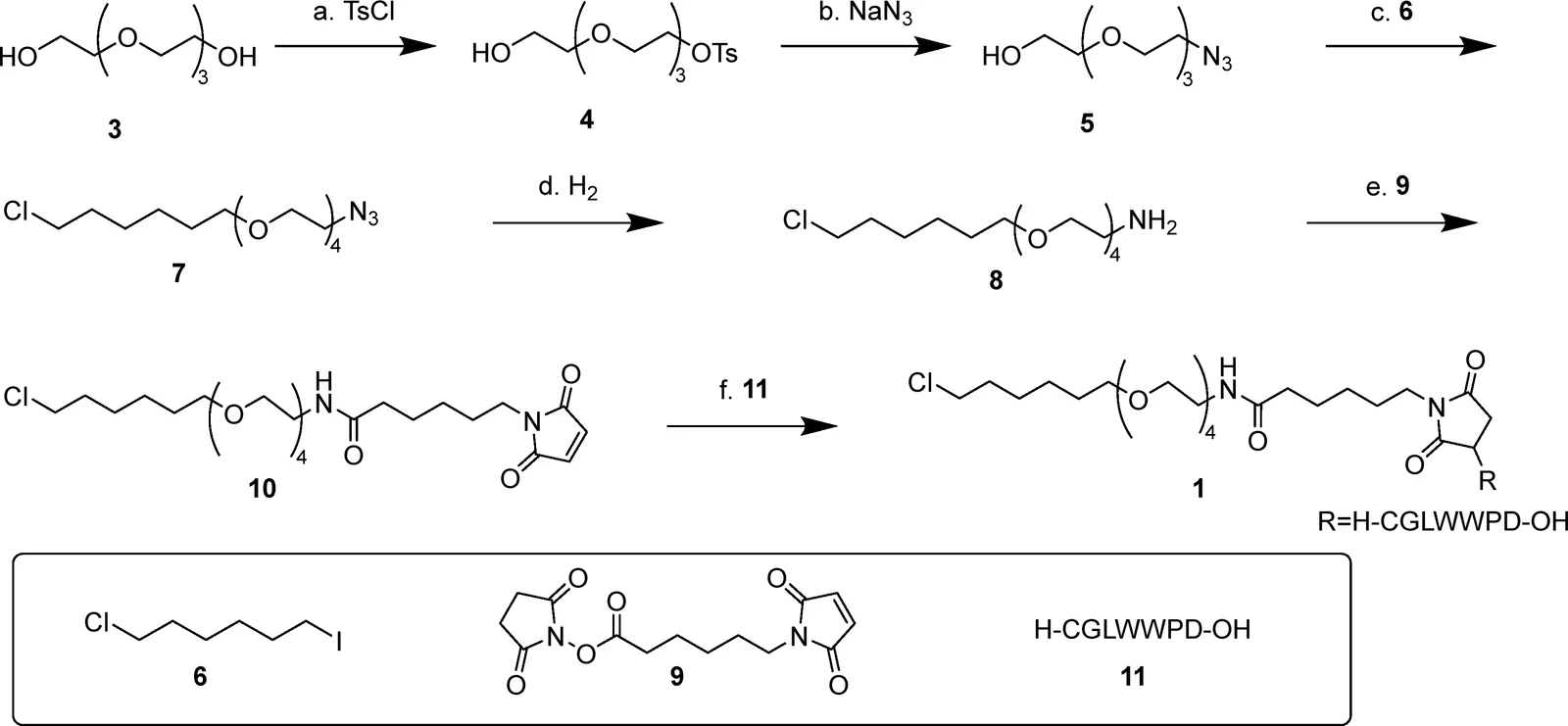
Synthesis of compound 1. (a) TsCl, NaOH, THF, 0 °C, 44%; (b) NaN_3_, DMF, 60 °C; (c) NaH, 6, DMF, 0 °C, 57% in 2 steps; (d) 10% Pd/C, H_2_, THF, r.t.; (e) DIPEA, 9, THF, r.t.; and (f) H-CGLWWPD-OH (11), NaPO_4_ buffer (pH 6.5), r.t., 98% in 3 steps.

To determine the affinity of 1 for CHIP, we conducted a saturation-binding fluorescence polarization (FP) assay using a fluorescent tracer (FITC-Ahx-LWWPD) and a recombinant His-tagged CHIP (His_6_-CHIP). 6-Aminohexanoic acid (Ahx) is a typical hydrophobic and flexible spacer. The FP was measured under the condition of 1 nM FITC-Ahx-LWWPD against varying concentrations of His_6_-CHIP (Fig. 2a). The calculated EC_50_ value was 29.0 nM and the *K*_d_ value was 28.8 nM. This is consistent with previously reported values.^57^ We conducted a competitive binding assay between FITC-Ahx-LWWPD and various concentrations of 1 in the presence of His_6_-CHIP. IC_50_ was calculated as 73.4 nM. We calculated the inhibition constant (*K*_i_) using the Nikolovska–Coleska equation,^68^ which accounts for the FP assay. The *K*_i_ value of 1 was 4.2 nM (Fig. 2b). These results show that 1 has a strong affinity for the CHIP protein, similar to that of the CHIPOpt peptide. To investigate whether CHIP and HaloTag form a ternary complex in the presence of 1, we conducted a pull-down assay using a purified recombinant GST-tagged HaloTag (GST-HT).^69^ GST-HT was preincubated with varying concentrations of 1. His_6_-CHIP was added to the GST-HT and 1 mixture. The ternary complex was captured using glutathione-Sepharose affinity beads (Fig. 2c). In the presence of 1, His_6_-CHIP was pulled down by GST-HT, whereas His_6_-CHIP was not pulled down in the absence of 1 (Fig. 2d). Optimal His_6_-CHIP pull-down occurred at 10 µM of 1, while higher concentrations (>10 µM) reduced the yield. This suggests a hook effect, which is frequently encountered with PROTAC molecules at high concentrations.^70^ The observation of this effect provides robust evidence of a ternary complex. The discrepancy between the IC_50_ value in the FP assay and the effective concentration in the pull-down assay arises from the difference in assay sensitivity. In the pull-down assay, a relatively high protein concentration (2 µM of His_6_-CHIP) was employed to ensure robust Coomassie brilliant blue (CBB) detection of the precipitated proteins. In this high-concentration regime, the observed effective concentration of 1 (1–10 µM) is physically constrained by the protein levels. Since the concentration required for a visible effect is typically expected to be at least 1/2 of protein concentration under these conditions, these results are stoichiometrically consistent with the assay parameters, despite the lower *K*_d_ values obtained in the FP assay.

**Fig. 2:**
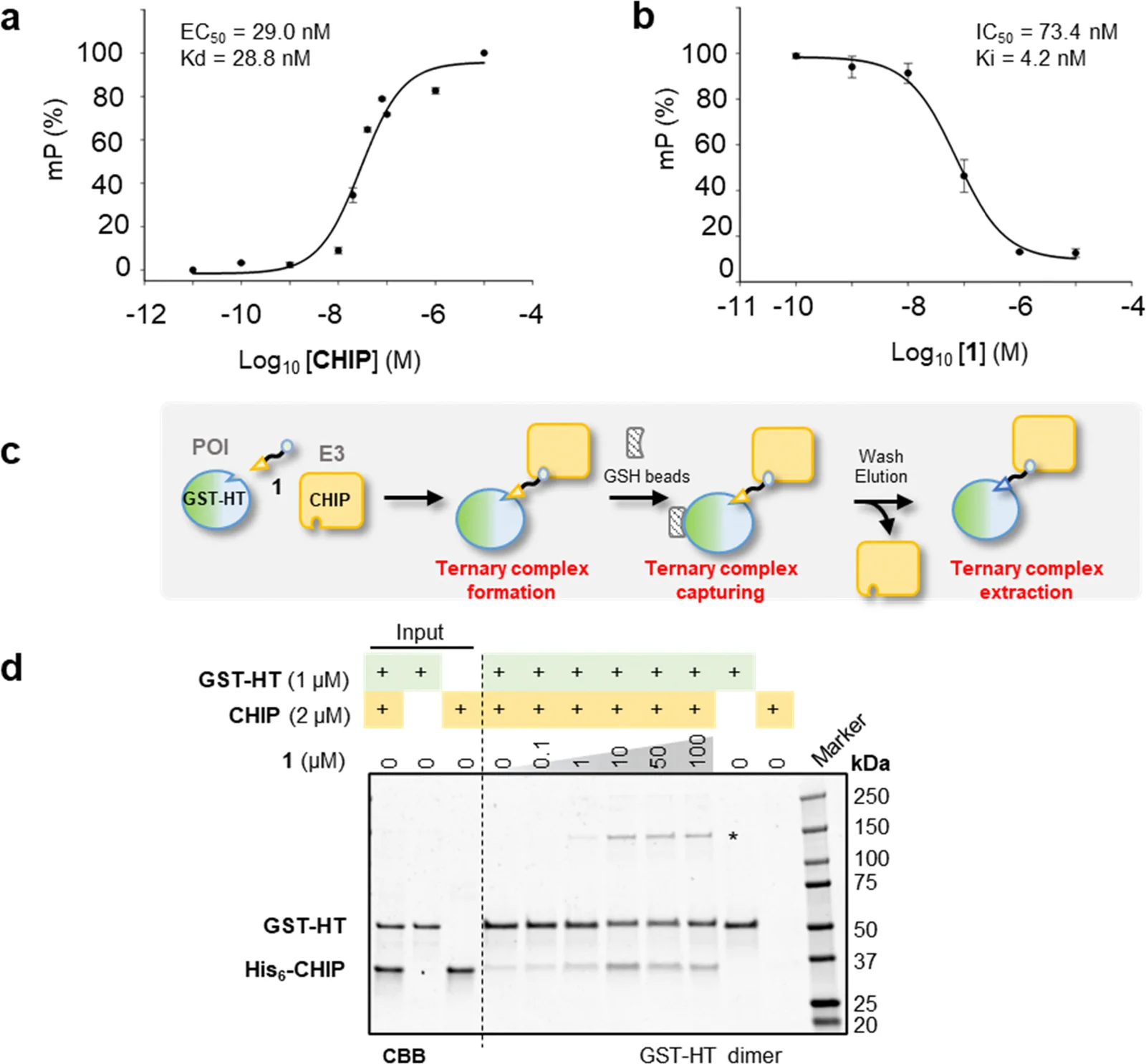
*In vitro* ternary complex formation between GST-HT, PROTAC 1, and CHIP. (a) Saturation fluorescence polarization (FP) assay. Normalized mP values for individual replicate samples (triplicate) were plotted. mP indicates milli polarization units. The fitting of the sigmoidal dose–response sigmoid curve and the calculation of apparent *K*_d_ were performed using the SigmaPlot software. Error bars represent standard error. (b) Competition binding FP assay. Normalized mP values for individual replicate samples (triplicate) were plotted and the fitting of one-site competition sigmoid curve was performed. The *K*_i_ value was calculated using eqn (5) (the details are provided in the Materials and method section). Error bars represent standard error. (c) Schematic of the *in vitro* ternary complex formation and pull-down. E3, E3 ligase; GSH, glutathione; and POI, protein of interest. (d) Ternary complex was separated by SDS-PAGE and detected by CBB staining. CHIP protein was co-eluted with GST-HT depending on the presence of 1. Asterisk, an unexpected band at 120 kDa might be the estimated GST-HT dimer.

Next, leveraging the ability of CHIP to act as a single-subunit E3 ligase without a scaffold protein, we investigated whether 1 promotes ubiquitylation of the target protein *in vitro*. Previous studies have shown that CHIP assembles various types of Ub chains depending on the E2 type.^71^ One E2 enzyme, UbcH5a-c, could induce all seven types of Lys-linked Ub chains on the substrate proteins.^72^*In vitro* ubiquitylation assay was conducted using E1 (GST-UBE1-His_8_), E2 (Ubc5Hc), E3 (His_6_-CHIP), and Ub in the presence of Mg^2+^ and ATP to assess the activity of 1 (Fig. 3a). To easily and exclusively detect the ubiquitylated GST-HT protein from the auto-ubiquitylated CHIP protein, GST-HT was labeled with fluorescein (GST-HT-FAM) (Fig. S1a). In the presence of 1 and His_6_-CHIP, CBB-stained gel showed a smeared band at a higher molecular weight (Fig. 3b). The fluorescence image of this gel clearly showed that the smeared band was derived from GST-HT-FAM (Fig. 3b). In addition, we tested whether the CHIP ligand peptide (11, H-CGL̲W̲W̲P̲D̲-OH) and the HaloTag ligand with linker (12)^73^ (Fig. 3b) could promote target ubiquitylation. Neither 11 nor a mixture of 11 and 12 promoted protein smearing; however, 12 slightly induced GST-HT-FAM ubiquitylation (Fig. 3b). A possible explanation is that a part of GST-HT-FAM undergoes a conformational change upon binding to the HT ligand. Since CHIP intrinsically interacts with and ubiquitylates non-native proteins,^74^ it may recognize 12-conjugated GST-HT-FAM as a substrate for ubiquitylation. These results indicate that 1 promotes GST-HT-FAM ubiquitylation *via* ternary complex formation. To evaluate the ubiquitylation efficiency, we performed an assay using varying concentration of 1. While a minor smear was observed even in the absence of 1 (0 µM), this band shift – representing poly-ubiquitylated GST-HT-FAM – increased dramatically in a concentration-dependent manner. A hook effect was observed at the highest concentration of 1 (2.5 µM) (Fig. S2). Time-course monitoring (0–240 min) revealed that ubiquitylation was detectable at 5 min and a plateau at 180 min (Fig. S3). To further investigate the poly-Ub chain architecture, GST-HT-FAM in a mixture of ubiquitylation components (E1, E2, E3, and Ub) was purified by 2-step purification using glutathione-Sepharose beads and Ni-Sepharose beads (Fig. S1b). The linkage of the Ub chain on GST-HT-FAM was analyzed by western blotting using an anti-Ub antibody and K48 and K63 chain-specific Ub antibodies. As expected, smearing was caused by polyubiquitylation and the Ub chain contained both K48 and K63 (Fig. 3d). It is still unclear whether the Ub chain induced by CHIP with 1 contains linkages other than K48 and K63, owing to the limitation of commercially available chain-specific antibodies. Taken together, these results show that CHIP can form a ternary complex with the target protein and ubiquitylate it with a mixed poly-Ub chain containing K48 and K63 in a 1-dependent manner *in vitro*.

**Fig. 3:**
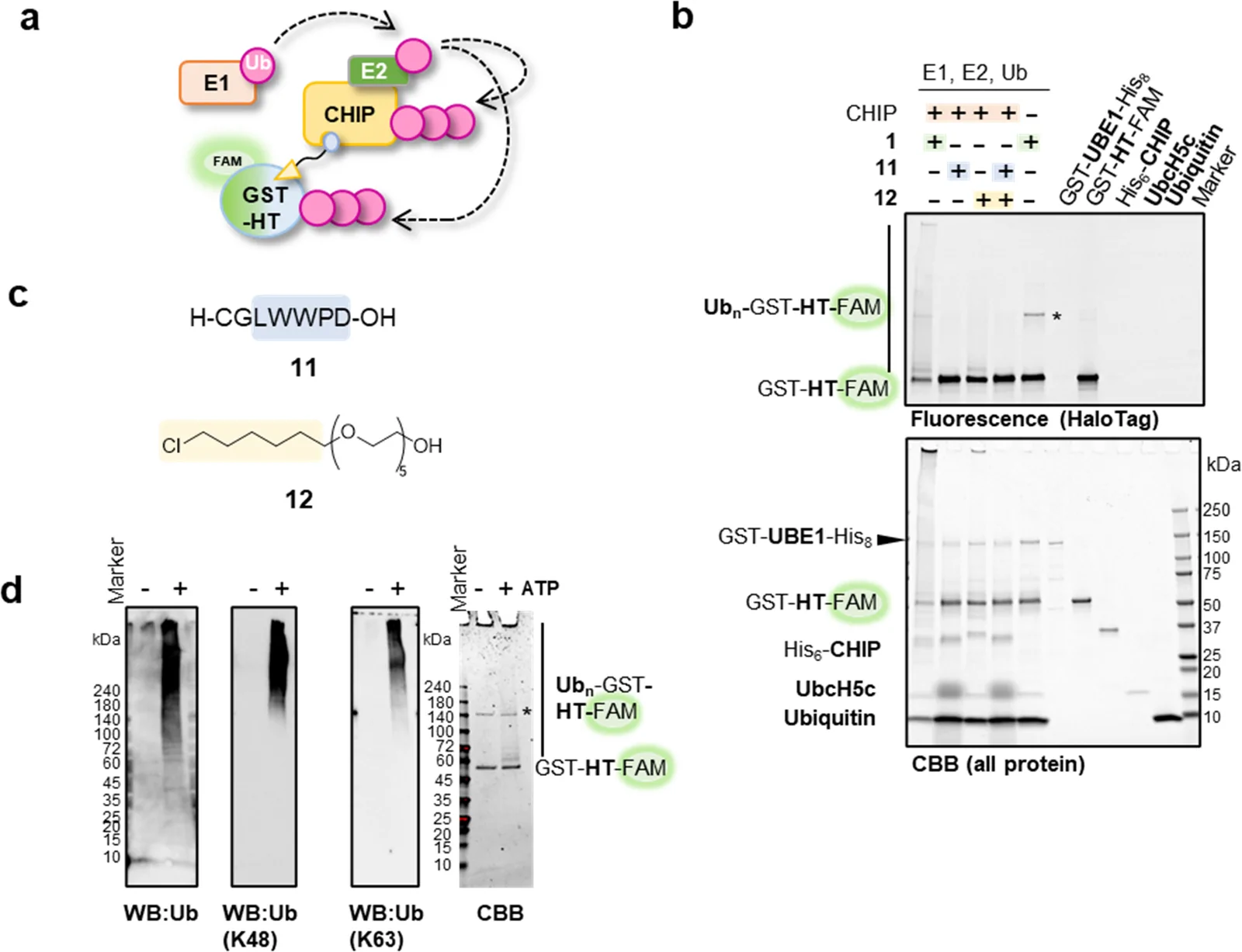
*In vitro* ubiquitylation assay. (a) Schematic of the ubiquitylation of GST-HT-FAM. His_6_-CHIP ubiquitylated GST-HT-FAM *via*1, in addition to CHIP auto-ubiquitylation. (b) GST-HT-FAM, E1 (GST-UBE1-His_8_), E2 (UbcH5c), His_6_-CHIP and Ub were mixed with 0.5 µM of 1, or each ligand (11, 12 or a mixture of those) and incubated at 37 °C for 2 h. GST-HT-FAM was separated by SDS-PAGE and visualized by the fluorescence of FAM and CBB staining. (c) Chemical structures of the CHIP ligand (11) and HaloTag ligand (12). Ligand 11 is a hepta-peptide (H-CGLWWPD-OH), including the CHIP TPR domain-binding sequence, LWWPD. Ligand 11 is composed of chlorohexane (HaloTag ligand) and a PEG linker. (d) Western blotting showed that the purified GST-HT-FAM from the Ub assay mixture was polyubiquitylated, and the Ub chains contained both K48 and K63 linkages. Ubiquitylation does not occur in the absence of ATP. The asterisk represents a GST-HT dimer.

We then investigated the intracellular degradation activity of 1. Given that the CHIP ligand (11) contains negatively charged aspartic acid and carboxyl groups—which often hinder cell permeability—we first assessed the cellular uptake of 1 using an HT-TMR ligand competition assay. While HT-TMR ligand normally labels intracellular HT, the presence of 1 should block this process through competitive covalent binding (Fig. S4a). Compound 1 inhibited TMR labeling in a concentration-dependent manner; notably, 8 µM of 1 reduced labeling to 15.0% within 1 hour (Fig. S4b and c). This confirms that 1 is cell-permeable and capable of binding to intracellular HT. Following this confirmation, we evaluated the degradation of a transiently expressed HT-emerald luciferase (HT-ELuc) fusion protein in HeLa cells. The degree of HaloTag degradation was estimated as the change in luciferase-mediated bioluminescence intensity.^73,75^ We examined 1 dose-dependency of HT-ELuc on cell degradation (Fig. 4a). Treatment with 0.1, 1 and 10 µM of 1 for 6 hours reduced the luminescence intensity to 0.82, 0.75 and 0.58 relative to the DMSO control (0 µM of 1), respectively. Then, we used HEK293 cells stably expressing HT-ELuc to investigate whether HaloTag degradation induced by 1 depends on the proteasome. In the presence of 3 µM of 1, the degradation of HT-ELuc was canceled by proteasome inhibitors (10 µM of MG132, 0.5 µM of bortezomib and 1 µM of epoxomicin) as 1.01, 1.08 and 1.00, respectively (Fig. 4b). These short-time exposure of proteasome inhibitors (6 hour treatment) did not affect cell viability (Fig. S5a and b). Taken together, these results showed that 1 promote the degradation of HT-ELuc *via* the UPS. This proof-of-concept study verified that CHIP E3 ligase can be utilized for TPD. In the 10 µM concentration of 1, the degradation rate was reduced (Fig. 4b). This reduction in HT-ELuc degradation may be due to the hook effect.^71^ Higher concentrations of PROTACs predominantly formed binary complexes with POI or E3 ligase, compromising POI degradation. Furthermore, the POI degradation activity in transiently expressing HeLa cells was higher than that in the stable HEK293 cell lines. The expression yields are typically higher when stably transfected cells are used. Excess POI expression masked POI degradation induced by PROTACs.

**Fig. 4:**
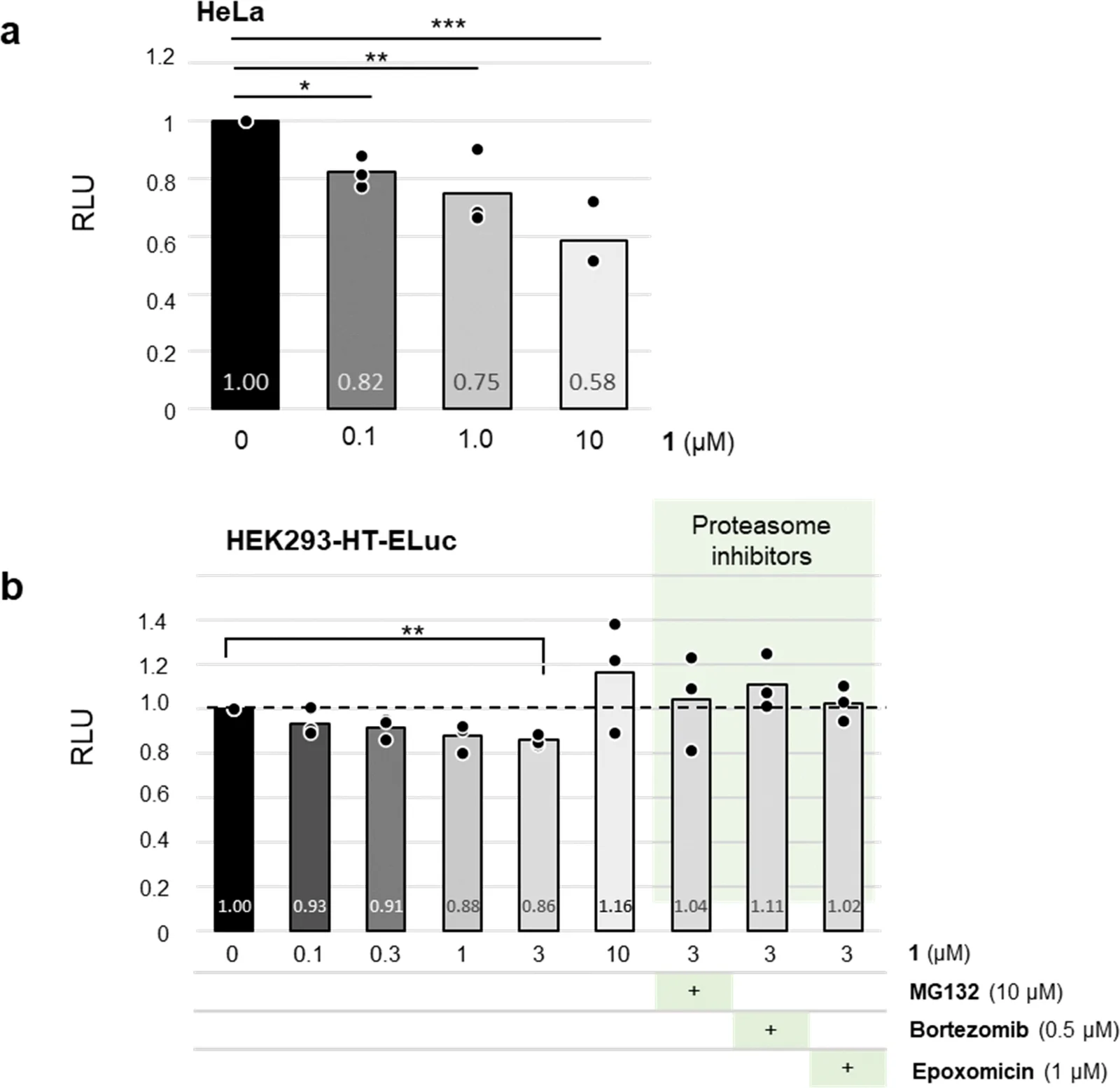
HaloTag degradation in human cell lines. (a) Transiently expressed HaloTag-conjugated Emerald luciferase (HT-ELuc) in HeLa cells was treated with 1 for 6 h. HaloTag degradation were estimated by the luciferase reporter assay. Luciferase assays were performed in triplicate, and the resulting signals were normalized to cell viability (measured by the WST-8 assay). Relative intensity unit (RLU) of control (0 µM of 1) was set as 1. Bar graph shows the mean of three independent experiments. Each data point is shown as a dot. (b) Degradation of constitutively expressed HT-ELuc in HEK293 cells induced by 3 µM of 1 was canceled by proteasome inhibitors (MG132, bortezomib and epoxomicin). Bar graph shows the mean of three independent experiments. Each data point is shown as a dot. *P* values between multiple groups were determined *via* one-way repeated measures ANOVA followed by *post hoc* Dunnett's test. Statistical difference between two groups was calculated using one-sample *t*-test (two-tailed). Asterisks represent significance as follows: *** <0.001, ** < 0.01, and * < 0.05.

To test whether PROTACs utilizing CHIP can induce the degradation of endogenous target proteins, we focused on the BRD4 protein, a well-established target for both cancer therapy and TPD. BRD4 regulates the translation and proliferation of cancer cells by recognizing acetylated histones. JQ1, a BRD4 inhibitor, blocked the interaction between BRD4 and acetylated histones. JQ1 suppressed cancer cell proliferation.^76^ To date, several PROTAC using JQ1 has been established.^12,13^ In this study, we designed and synthesized a BRD4 degrader (2). JQ1 and the CHIP ligand (LWWPD) were conjugated *via* a linker (Fig. 1 and Scheme 2). Carboxylic acid 13 was coupled with amine 14 under EDCI-HOBt conditions to obtain the intermediate 15. The deprotection of *N*-Boc and coupling with maleimide-containing carboxylic acid 16 afforded the precursor 17. Finally, Michael addition with peptide 11 afforded the target molecule 2.

**Scheme 2:**
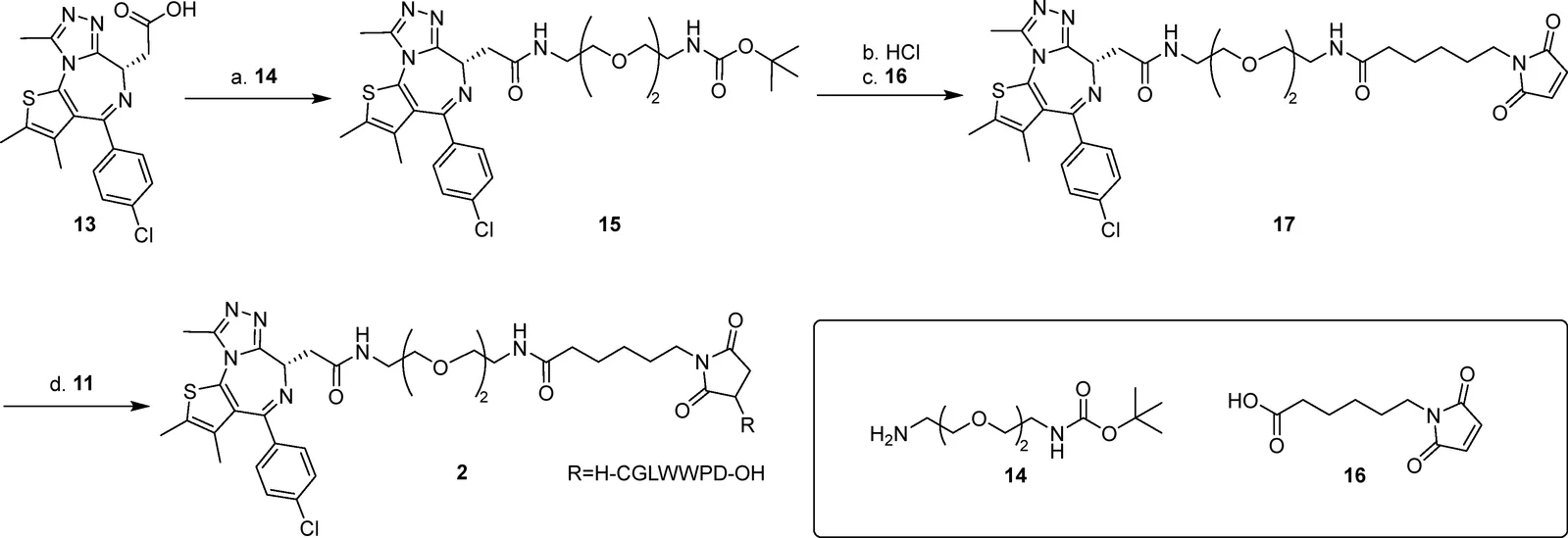
Synthesis of compound 2. (a) HOBt, EDC-HCl, DIPEA, THF, 14, 0 °C to r.t.; (b) HCl, CPME, DCM, r.t.; (c) HATU, DIPEA, DCM, 16, r.t., 73% in 3 steps; and (d) 11, Na–PO_4_ buffer (pH 6.8), r.t., 63%.

We examined whether 2 facilitates the formation of a ternary complex with BRD4 and CHIP *in vitro*. Recombinant His_6_-CHIP was preincubated with 2. To form the ternary complex, a mixture of His_6_-CHIP and 2 was combined with HeLa lysate containing the endogenous BRD4 protein. His_6_-CHIP and its interacting molecules were captured using Ni-ion affinity beads and eluted with imidazole. BRD4 was pulled down using His_6_-CHIP in the presence of 2. In the absence of 2, BRD4 was not detected in the eluted fraction (Fig. 5a and Fig. S6). This indicated that the detected BRD4 was one of the molecules that interacted with His_6_-CHIP *via*2. Next, we examined 2 dose-dependency and time course of BRD4 degradation in HeLa cells. HeLa cells were treated with 0, 0.1, 1 and 10 µM of 2 for 6 or 24 h. BRD4 protein levels were evaluated using western blot analysis. Markedly BRD4 degradation occurred in 6 hours in the presence of 1 µM of 2 (Fig. 5b). The quantification of the intensity of bands showed that BRD4 decreased to 0.65 in 6 h and 0.57 in 24 h with 1 µM of 2, relative to that of 0 µM control. The degradation efficiency at a higher concentration of 2 (10 µM) was slightly lower than that at 1 µM for both 6 and 24 hour treatments (0.69 and 0.64, respectively) (Fig. 5c). This reduction in BRD4 degradation with the higher concentration of 2 suggested the hook effect. These treatment conditions (0.1, 1 and 10 µM of 2) did not show any cell toxicity. We evaluated the selectivity of compound 2-induced degradation among BET family proteins. Specifically, we examined whether BRD2 and BRD3 are also degraded by compound 2. The results revealed that both BRD2 and BRD3 were degraded to an extent comparable to that of BRD4 (Fig. S7), indicating a lack of the BRD4 selectivity typically observed with MZ1.^77^ While JQ1 is a pan-bromodomain inhibitor, the isoform selectivity of JQ1-based PROTACs within the BET family is known to vary depending on factors such as the recruited E3 ligase and the stability of the resulting ternary complex. For instance, the BRD4 selectivity of the VHL-recruiting PROTAC, MZ1, is attributed to the formation of a stable ternary complex with BRD4.^78^ Conversely, the CRBN-recruiting PROTAC, dBET1, non-selectively degrades all BET family members.^12^ Similarly, compound 2, which recruits CHIP, degraded the entire BET family without exhibiting BRD4 preference. This absence of selectivity properties may stem from the differences in ternary complex stability governed by both the structural properties of the CHIP ligase and the specific PROTA architecture, including linker geometry. Then, to examine the mechanism of BRD4 degradation induced by 2, HeLa cells were treated with proteasome inhibitors (MG132 and bortezomib) 1 hour prior to compound 2 treatment. Proteasome inhibitors abrogated compound 2-induced BRD4 degradation (Fig. 5d). Excess CHIP ligands (20 and 100 times to 2 concentrations) also inhibited BRD4 degradation. In addition, the BRD4 ligand (JQ1-COOH) (13), CHIP ligand (11), or a mixture of these did not reduce BRD4 levels (Fig. 5d). These results suggested that 2 incorporated into the cell induced endogenous BRD4 degradation *via* the UPS. In addition, the chimeric structures of the E3 ligase ligands and POI ligand were necessary for BRD4 degradation. While the development of cell-permeable CHIP ligands remains challenging, as previously reported for macrocyclic peptides by Ng *et al.*,^79^ our results demonstrate that the peptidyl ligands utilized in this study possess sufficient membrane permeability for intracellular activity. Specifically, the intracellular internalization and subsequent target engagement were demonstrated *via* a HaloTag-covalent bonding assay (Fig. S4) and further supported by functional competition studies (Fig. 5d). The fact that an excess of unconjugated CHIP ligands (11, H-CGLWWPD-OH) prevented BRD4 degradation strongly suggests that our linear peptide sequence is capable of reaching the cytosol to compete for CHIP binding, thereby enabling effective targeted protein degradation. To confirm that CHIP is responsible for the degradation of the target protein, we evaluated compound 2-induced BRD4 degradation in a CHIP-knockout (CHIP-KO) cell line. Although BRD4 was degraded in wild-type (WT) HeLa cells, this degradation was abolished in the CHIP-KO cell line (Fig. S8). Consequently, we confirmed that the compound 2-induced degradation of BRD4 is dependent on CHIP E3 ligase.

**Fig. 5:**
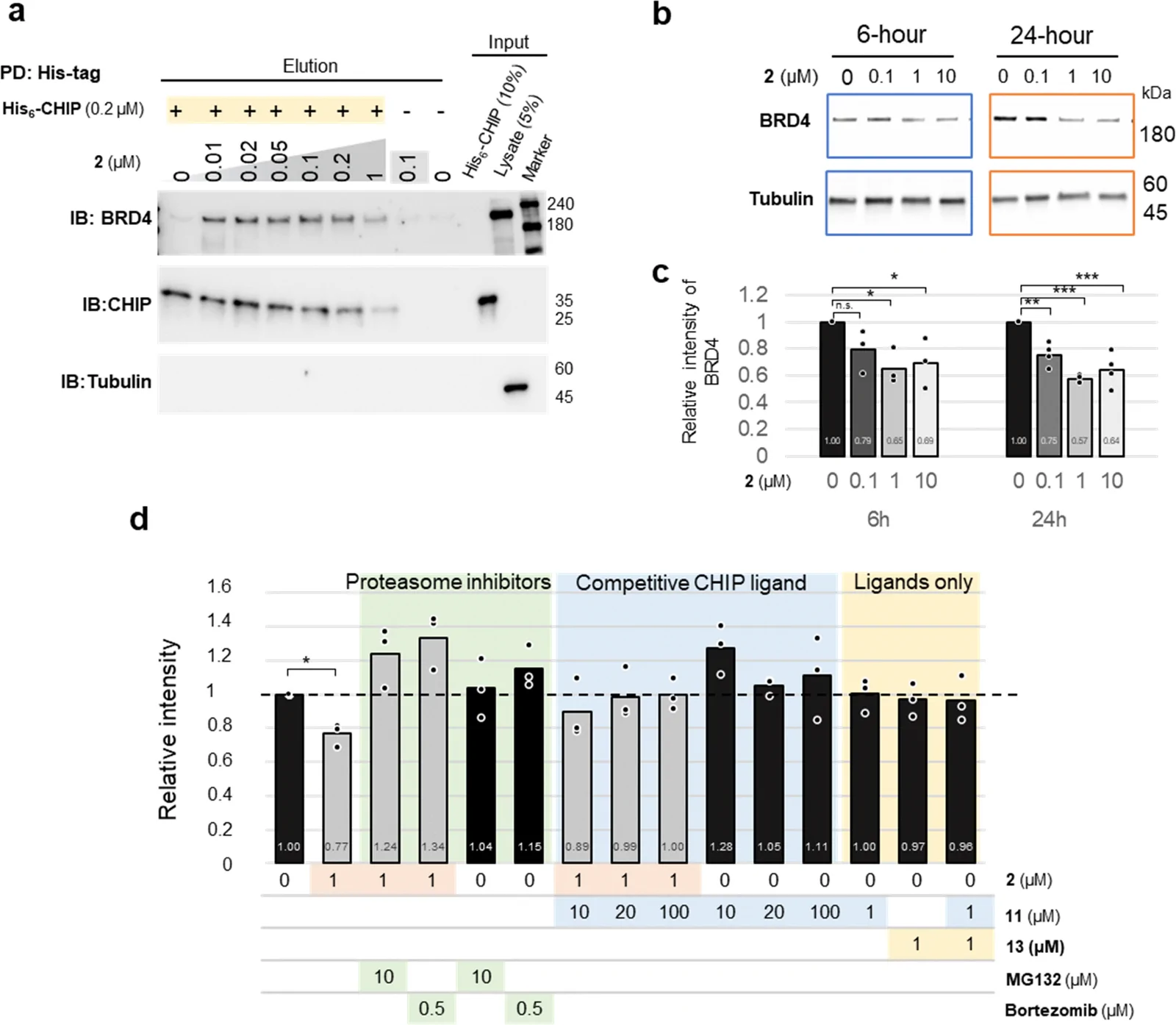
BRD4 degradation in HeLa cells. (a) Representative images of the *in vitro* ternary complex formation from three independent experiments. Pre-incubated His_6_-CHIP and 2 mixtures were incubated with HeLa cell lysate. The ternary complex was pulled down using His-tag affinity beads. BRD4, His_6_-CHIP, and tubulin were detected using western blotting. BRD4 co-precipitated with His_6_-CHIP in the presence of compound 2. (b) Representative western blots of three (6 h) and four replicates (24 h). Blots showed BRD4 levels in HeLa cells at the indicated concentrations of 2. (c) Bar graphs represent quantitative data of BRD4 degradation from three (6 h) and four (24 h) replicates. Band intensities of BRD4 were normalized to those of tubulin. The relative intensity of control (0 µM of 1) was set as 1. Bar graph shows the mean of independent experiments. (d) Degradation of BRD4 protein (6 hour treatment with 2) was inhibited by proteasome inhibitors (MG132 and bortezomib) and excess CHIP ligand (11). 11 and BRD4 ligand (JQ1 carboxylic acid) (13) or a mixture of these ligands did not induce BRD4 degradation. Bar graph shows mean of three independent experiments. Each data point is shown as a dot. *P* values between multiple groups were determined *via* one-way repeated measures ANOVA followed by *post hoc* Dunnett's test. Statistical difference between two groups was calculated using a one-sample *t*-test (two-tailed). Asterisks represent significance as follows: *** <0.001, ** < 0.01, and * < 0.05. n.s. stands for “not statistically significant” (*p* ≥ 0.05).

To further understand the mechanism of BRD4 degradation, we tested whether other E3 ligases involve the BRD4 degradation pathway. E3 ligases are classified into four groups: HECT (homologous to the E6AP carboxyl terminus), RING (really interesting new gene), U-box, and RBR (RING-IBR-RING) E3 ligases.^80^ Although CHIP is categorized as a U-box-type E3 ligase, the RING E3 ligase is one of the major groups of E3-characterized RING domain. Cullin-RING ligases (CRLs) are multimeric E3-containing Cullin scaffold proteins that are activated by neddylation.^22^ MLN4924 is an inhibitor of neural precursor cell-expressed developmentally downregulated protein 8 (NEDD8) activation enzyme (NAE). MLN4924 inhibits CRL E3 ligase activity by blocking the NEDDylation of Cullins.^81^ HeLa cells were treated with MLN4924 1 hour prior to 2 or dBET1 addition. dBET1 is a potent PROTAC-connected CRBN and BRD4 ligand (JQ1).^12^ CRBN is a member of the CRL4^CRBN^ E3 ligase belonging to CRLs. BRD4 degradation by dBET1 was almost completely abolished by treatment with MLN4924. In contrast, 2-induced BRD4 degradation was not significantly inhibited by MLN4924 (Fig. S9a and b). These results confirm that CRLs are not likely to be involved in BRD4 degradation induced by 2.

Although 1 and 2 succeed in reduced levels of HT-ELuc and BRD4 proteins, the degree of degradation efficacy remains less than 50%. Possible explanations for this moderate degradation activity include instability of the peptidyl CHIP ligand in the cell and unexpected interactions between the CHIP ligand and other proteins. In general, most linear peptides composed of l-amino acids exhibit short intracellular half-life (5–30 min).^82^ Although such l-peptides are generally unstable in cells, the CHIP-interacting pentapeptide (IEEVD) has been reported to remain stable for up to 6 hours in HEK293T cells.^83^ In addition to the stability of peptidyl ligand moiety, thiosuccinimide linkage connecting the linker and the peptide is susceptible to degradation under physiological conditions with high glutathione (GSH) concentrations.^84^ In the cytosol, where the GSH levels are elevated, thiol exchange and retro-Michael reactions can lead to PROTAC instability. These structural and metabolic properties may account for the moderate degradation efficiency observed in our study. For the second point, similar to CHIP, Hsp70–Hsp90 organizing protein (HOP) recognizes the C-terminus of Hsp70/Hsp90 (M/IEEVD) through its TPR domains.^29^ HOP is involved in the refolding of the Hsp70/Hsp90 client protein.^85^ In cases where target proteins contain unfolded or disordered regions, HOP might induce degradation rather than refolding of target proteins. In addition to CHIP or HOP, some E3 ligases interact with chaperone proteins such as CUL5^86^ and E6-AP.^87^ It is possible that these additional E3 ligases play a role in TPD cooperation with CHIP.

## Conclusion

The scarcity of novel PROTACable E3 ligase candidates is a research hotspot. In this study, we focused on CHIP E3 ligase, which is involved in the degradation of disease-related and unfolded proteins. We designed and synthesized PROTACs 1 and 2 composed of an LWWPD pentapeptide sequence as a CHIP ligand and ligands for target proteins (*i.e.* a well-established tag-protein, HaloTag, and a therapeutic target, BRD4), *via* a linker. Both 1 and 2 formed stable ternary complexes with CHIP and its target proteins, and induced ubiquitylation *in vitro*. In mammalian cells, 1 and 2 induced target protein degradation at 1 µM within 6 h in a proteasome- and CHIP-dependent manner. These results demonstrate, for the first time, that CHIP E3 ligase can be utilized as PROTACs. Moreover, CHIP ligands remain to be improved in terms of biological stability, binding specificity, molecular weight, and cell permeability for more efficient degradation of target proteins. Recently, Lucas *et al.* have identified small-molecule CHIP binders.^52^ Thus, this potent CHIP ligand may be useful as a warhead for PROTAC.

## Experimental

### Chemistry

#### General

All the chemicals were obtained from commercial sources and used without further purification, except for DMF, acetonitrile, and ethanol. Briefly, DMF was dried by passage through molecular sieves 4A. Acetonitrile and ethanol were dried by passage through molecular sieves 3A. The reactions were monitored by TLC. Routine TLC was performed using silica gel 60 F254 plates (Merck, Germany). Flash chromatography was performed using the indicated solvent system (Isolera, Biotage) on silica gel 60 N (spherical, neutral, particle size 40–50 µm) (Kanto Chemical. Co. Inc., Japan) or Sfär Silica HC D (High-Capacity Duo 20 µm) (Biotage, Sweden). ^1^H NMR spectra were recorded using a JEOL JNM ECA-600 spectrometer (600 MHz). Chemical shifts (*δ*) are reported in ppm relative to the internal TMS signal (0.00 ppm) for CDCl_3_ solutions (7.26 ppm for ^1^H). Multiplicities are reported using the following abbreviations: s, singlet; d, doublet; t, triplet; dd, double doublet; m, multiplet; br, broad; and *J*, coupling constant in hertz. Preparative TLC (PTLC) was performed on a glass plate of PLC Silica Gel 60 F254 (Merck, Germany). Analytical HPLC was carried out using a JASCO PU-980 HPLC Pump and an SSC-3315 Degassing Unit with a JASCO MD-2018 Plus Photodiode Array Detector, an SSC-2120 Column Oven, a JASCO AS-4050 HPLC Autosampler, and a JASCO LC-NetII/ADC Interface Box using a C18 reverse phase column (Inertsil ODS-4, 150 × 4.6 mm, 5 µm; GL Science Inc., Japan) with mobile phase A: 0.1% formic acid (FA) in H_2_O and mobile phase B: acetonitrile or methanol. The injection volume was 20 µL, the flow rate was 1.0 mL min^−1^, and the column temperature was 40 °C. Purity was determined by measuring the absorbance at 220 nm. Preparative HPLC was carried out using a JASCO LC-Net II/ADC Interface Box, an FCC Fraction Collector Controller, a CHF122SC fraction collector, an FV-4000-06 Fraction valve unit, a UV-4075 UV/Vis Detector and a PU-4086 Semi-preparative Pump using a C18 reverse phase column (InertSustain 20 × 250 mm, 5 µm; GL Science Inc.). Gel permeation chromatography (GPC) was performed using a LaboACE LC-5060 column (Japan Analytical Industry Co., Ltd, Japan) with two PlusJAIGEL-GS320 columns. ESI-MS was performed using a micrOTOF spectrometer (Bruker, MA, USA). MALDI-MS was performed using an AB Sciex TOF/TOF 5800 (Sciex, MA) and Autoflex Speed-S1 (Bruker Daltonics, MA).

### Synthesis

#### Compound 1

##### 2-(2-(2-(2-Hydroxyethoxy)ethoxy)ethoxy)ethyl 4-methylbenzenesulfonate (4)

The solution of tetraethylene glycol (3, 4.5 mL, 26.3 mmol) in THF (1 mL) was kept cool (*i.e.*, at 0 °C). Sodium hydroxide (373 mg, 9.32 mmol) and the mixture of 4-methylbenzenesulfonyl chloride (1.00 g, 5.25 mmol) in THF (10 mL) were added into the reaction mixture. The reaction was held at 0 °C for 1 h, and then, it was held at r.t. for 1 h. The reaction was then quenched with ice-cold water. The reaction mixture was diluted with ethyl acetate (AcOEt) and ice-cold water. The organic layer was washed with ice water, dried over Na_2_SO_4_, filtered, and concentrated under vacuum. The concentrate was purified using flash column chromatography. 4 (0.797 g, 2.29 mmol, 44%) was obtained as a clear light green oil. ^1^H-NMR (600 MHz, CDCl_3_) *δ* 7.78 (d, *J* = 8.3 Hz, 2H), 7.33 (d, *J* = 8.3 Hz, 2H), 4.15 (dd, *J* = 5.4, 4.5 Hz, 2H), 3.69–3.67 (m, 4H), 3.63 (ddd, *J* = 17.0, 5.5, 3.7 Hz, 4H), 3.58 (t, *J* = 4.1 Hz, 6H), 2.50–2.60 (1H), 2.43 (s, 3H). MS (ESI) *m/z* 371 (M + Na)^+^.

##### 2-(2-(2-(2-Azidoethoxy)ethoxy)ethoxy)ethan-1-ol (5)

A solution of sodium azide (149.9 mg, 2.306 mmol) was slowly added to a solution of 4 (517 mg, 1.48 mmol) in DMF (4 mL). The reaction was held at 60 °C for 3 h. The reaction mixture was diluted with EtOAc, and toluene was concentrated under vacuum. The concentrate was purified using flash column chromatography. 5 (328 mg, 1.50 mmol) was obtained as a colorless oil. ^1^H NMR (600 MHz, CDCl_3_) data are identical to those previously reported. ^1^H-NMR (600 MHz, CDCl_3_) *δ* 3.73 (dd, *J* = 9.2, 5.5 Hz, 2H), 3.70–3.66 (m, 11H), 3.62–3.61 (m, 2H), 3.40 (t, *J* = 5.0 Hz, 2H). MS (ESI) *m/z* 242 (M + Na)^+^.

##### 1-Azido-18-chloro-3,6,9,12-tetraoxaoctadecane (7)

NaH (69.888 mg, 2.9 mmol) was added to a solution of 5 (229 mg, 1.05 mmol) in 1.0 mL of DMF at 0 °C in inert gas. After stirring at 0 °C for 30 min, to a solution of 1-chrolo-6-iodohexane (6) (440 mg, 271 µL, 1.78 mmol) in 1.0 mL of DMF was added the reaction mixture at 0 °C. After stirring at r.t. for 2 h, the reaction was quenched by adding an aqueous HCl solution (1 M) and AcOEt, extracted with AcOEt and brine, dried over Na_2_SO_4_, filtered, and concentrated under vacuum. The residue was purified by silica gel chromatography using a gradient of hexane : EtOAc = 3 : 1–1 : 3. 7 was obtained as a yellow oil (199.4 mg, 0.5902 mmol, 57% in two steps). ^1^H-NMR (600 MHz, CDCl_3_) *δ* 3.68–3.62 (m, 12H), 3.56 (t, *J* = 5.0 Hz, 2H), 3.51 (t, *J* = 6.6 Hz, 2H), 3.44 (t, *J* = 6.6 Hz, 2H), 3.37 (t, *J* = 5.0 Hz, 2H), 1.78–1.73 (m, 2H), 1.60–1.55 (m, 2H), 1.46–1.41 (m, 2H), 1.38–1.33 (m, 2H). MS (ESI) *m/z* 360 (M + Na)^+^.

##### 18-Chloro-3,6,9,12-tetraoxaoctadecan-1-amine (8)

Palladium-activated carbon (Pd 10%) (5.6 mg, 0.148 mmol) was added to a solution of 7 (50 mg, 0.148 mmol) in 1 mL of THF under H_2_ atmosphere (balloon). After stirring at r.t. for 2 h, the reaction mixture was filtered and concentrated under vacuum. 8 was obtained as a yellow oil (44.6 mg, 0.143 mmol). ^1^H-NMR (600 MHz, CDCl_3_) *δ* 3.68–3.57 (m, 12H), 3.54–3.50 (m, 4H), 3.45 (t, *J* = 6.6 Hz, 2H), 2.86 (t, *J* = 5.3 Hz, 2H), 1.79–1.75 (m, 2H), 1.62–1.57 (m, 4H), 1.47–1.42 (m, 2H), 1.39–1.34 (m, 2H). MS (ESI) *m/z* 312 (M + H)^+^.

##### *N*-(18-Chloro-3,6,9,12-tetraoxaoctadecyl)-6-(2,5-dioxo-2,5-dihydro-1*H*-pyrrol-1-yl)hexanamide (10)

A mixture of 8 and 2,5-dioxopyrrolidin-1-yl 6-(2,5-dioxo-2,5-dihydro-1*H*-pyrrol-1-yl)hexanoate (9, 3.1 mg, 0.010 mmol) in 1 mL of THF were stirred at r.t. for 48 h. *N*,*N*-Diisopropylethylamine (DIPEA) (1.73 µL, 9.94 µmol) was added and stirred at r.t. for 1 h. After the addition of 9 (30.6 mg, 0.990 mmol), the reaction was maintained at r.t. for 2 h and stopped by evaporation. The residue was purified by silica gel chromatography with the gradient of chloroform : methanol = 99.2 : 0.8 to 93.6 : 6.4. 10 was obtained as a yellow oil (36.93 mg, 73.129 µmol). ^1^H-NMR (600 MHz, CDCl_3_) *δ* 6.68 (s, 2H), 6.15 (s, 1H), 3.66–3.61 (m, 10H), 3.59–3.50 (m, 8H), 3.46–3.43 (m, 4H), 2.16 (t, *J* = 7.6 Hz, 2H), 1.80–1.75 (m, 2H), 1.68–1.63 (m, 4H), 1.61–1.57 (m, 2H), 1.47–1.29 (m, 6H). MS (ESI) *m/z* 527 (M + H)^+^.

##### *S*-(1-(25-Chloro-6-oxo-10,13,16,19-tetraoxa-7-azapentacosyl)-2,5-dioxopyrrolidin-3-yl)-l-cysteinylglycyl-l-leucyl-l-tryptophyl-l-tryptophyl-l-prolyl-l-aspartic acid (1)

Cysteine-maleimide Michael addition reaction was conducted in 100 mM Na–PO_4_ buffer (pH 6.5) with 4.9% acetonitrile and 6.9% DMSO. Prior to the addition of 11 (H-CGLWWPD-OH) (3.6 mg, 4.1 µmol, 1 eq.) and 10 (3.7 mg, 7.35 µmol, 1.8 eq.), they were dissolved in DMSO. The reaction mixture was then incubated at r.t. for 20 h. The reaction mixture was dried in a SpeedVac for 3 h and freeze-dried for 18 h. The sample was purified using GPC. GPC was performed at a methanol : TFA ratio of 100 : 0.1. Products containing the fractions were collected and concentrated under vacuum. Samples dissolved in DMSO were diluted with acetonitrile : water : TFA = 80 : 20 : 0.1 and then mixed with 1 µL of α-cyano-4-hydroxyciannamic acid (CHCA) solution (5 mg mL^−1^ in acetonitrile : 0.1% TFAaq. = 50 : 50) on a MALDI-TOF plate. The peaks of the modified peptides were detected using MALDI-TOF analysis (Autoflex Speed-S1, Bruker Daltonics). 1 was obtained as a yellow oil (4.1 mg, 2.7 µmol, 98% in 3 steps) MALDI-TOF *m/z* = 1402.406 (M + Na)^+^. Purity was 95.5%.

#### Compound 2

##### *tert*-Butyl(*S*)-(2-(2-(2-(2-(4-(4-chlorophenyl)-2,3,9-trimethyl-6*H*-thieno[3,2-*f*][1,2,4]triazolo[4,3-*a*][1,4]diazepin-6-yl)acetamido)ethoxy)ethoxy)ethyl)carbamate (15)

*tert*-Butyl (2-(2-(2-aminoethoxy)ethoxy)ethyl)carbamate (14) was added to a mixture of (*S*)-2(4-(4-chlorophenyl)-2,3,9-trimethyl-6*H*-thieno[3,2-*f*][1,2,4]triazolo[4,3-*a*][1,4]diazepin-6-yl)acetic acid (13) (43.2 mg, 108 µmol), 1-(3-dimethylaminopropyl)-3-ethylcarbodiimide hydrochloride (EDC·HCl) (54.0 mg, 28.0 µmol), 1-hydroxybenzotriazole (HOBt) (49.7 mg, 32.5 µmol) and DIPEA (37 µL, 0.21 mmol) in THF (2 mL) at 0 °C. The reaction mixture was stirred overnight at r.t. The reaction mixture was diluted with water and EtOAc. The organic layer was washed with water, dried over Na_2_SO_4_, filtered, and concentrated under vacuum. The concentrate was purified using flash column chromatography (CHCl_3_/MeOH). Although some impurities were detected by NMR spectroscopy, crude 15 was used in subsequent reactions without further purification. ^1^H-NMR (600 MHz, CDCl_3_) *δ* 7.41 (d, *J* = 8.3 Hz, 2H), 7.33 (d, *J* = 8.7 Hz, 2H), 4.64 (s, 1H), 3.64–3.51 (m, 11H), 3.35 (s, 3H), 2.66 (s, 3H), 2.40 (s, 3H), 1.67 (s, 3H), 1.46–1.42 (m, 9H) MS (ESI) *m/z* 653 (M + Na)^+^.

##### (*S*)-*N*-(2-(2-(2-(2-(4-(4-Chlorophenyl)-2,3,9-trimethyl-6*H*-thieno[3,2-*f*][1,2,4]triazolo[4,3-*a*][1,4]diazepin-6-yl)acetamido)ethoxy)ethoxy)ethyl)-6-(2,5-dioxo-2,5dihydro-1*H*-pyrrol-1-yl)hexanamide (17)

A solution of HCl (1.3 mmol) in cyclopentyl methyl ether (CPME) (312 µL) was added to a solution of crude 15 (77.8 mg, 125 µmol) in THF (2 mL) and stirred at r.t. overnight. The reaction mixture was then concentrated under vacuum. The product was used in the next step without further purification. DIPEA (174 µL, 1.00 mmol) and 1-[bis(dimethylamino)methylene]-1*H*-1,2,3-triazolo[4,5-*b*]pyridinium 3-oxide hexafluorophosphate (HATU) (120 mg, 317 µmol) were added to the mixture of the crude product and 6-maleimidohexanoic acid (16, 57.7 mg, 273 mol) in DCM (2 mL). The reaction mixture was stirred overnight at rt. The reaction mixture was purified by PTLC (CHCl_3_/MeOH, 20 : 1). 17 (66.3 mg 91.5 µmol, 73% in 3 steps) was obtained as a brown amorphous. ^1^H-NMR (600 MHz, CDCl_3_) *δ* 7.40 (d, *J* = 8.7 Hz, 2H), 7.32 (d, *J* = 8.7 Hz, 2H), 7.18 (t, *J* = 5.5 Hz, 1H), 6.76 (t, *J* = 5.5 Hz, 1H), 6.68–6.65 (m, 2H), 4.64 (dd, *J* = 8.3, 6.0 Hz, 1H), 3.66–3.31 (m, 16H), 2.66 (s, 3H), 2.40 (s, 3H), 2.15 (t, *J* = 7.6 Hz, 2H), 1.67 (s, 3H), 1.63–1.58 (m, 2H), 1.55–1.50 (m, 2H), 1.27–1.22 (m, 2H) MS (ESI) *m/z* 746 (M + Na)^+^.

##### *S*-(1-(1-((*S*)-4-(4-Chlorophenyl)-2,3,9-trimethyl-6*H*-thieno[3,2-*f*][1,2,4]triazolo[4,3-*a*][1,4]diazepin-6-yl)-2,13-dioxo-6,9-dioxa-3,12-diazaoctadecan-18-yl)-2,5-dioxopyrrolidin-3-yl)-l-cysteinylglycyl-l-leucyl-l-tryptophyl-l-tryptophyl-l-prolyl-l-aspartic acid (2)

Cysteine-maleimide Michael addition reaction was conducted in 100 mM Na–PO_4_ buffer (pH 6.5) with 8% acetonitrile and 11.5% DMSO. Prior to addition, 11 (H-CGLWWPD-OH) (2.38 mg, 2.72 µmol, 1 eq.) and 17 (6.42 mg, 8.86 µmol, 3.26 eq.) were diluted with DMSO. The reaction mixture was then incubated at r.t. for 20 h. The crude reaction mixture was dried using a SpeedVac centrifugal evaporator (Thermo Fisher Scientific) under vacuum for 3 h and freeze-dried for 18 h. The samples were purified using GPC. GPC was performed using a LaboACE LC-5060 (PlusJAIGEL-GS320) with methanol/TFA (100/0.1) as the eluent. Products containing the fractions were collected and concentrated under vacuum. The sample dissolved in DMSO was diluted with acetonitrile : water : TFA = 80 : 20 : 0.1 and then mixed with 1 µL of CHCA solution on a MALDI-TOF plate. The peaks of the modified peptides were detected using MALDI-TOF analysis (Autoflex Speed-S1, Bruker Daltonics). 2 was obtained as a yellow oil (2.9 mg, 2.72 µmol, 63%). MALDI-TOF *m/z* = 1621.244 (M + Na)^+^. Purity was 95.2% (Table 1).

**Table 1:** Details regarding reagents used in this study

Reagent or resource	Source	Identifier
**Antibodies**		
Anti-BRD4 (E2A7X)	Cell signaling technology	13440
Anti-Tubulin nFAB Rhodamine	BIO-RAD	12004164
Anti-Ub	Proteintech	10201-2-AP
Anti-Ub K48 linkage specific	Abcam	ab140601
Anti-Ub K63 specific	EMD Millipore	05-1308
Rabbit IgG StarBright Blue 700	BIO-RAD	12004162
Peroxidase conjugate Anti-Rabbit IgG (H + L) antibody	SeraCare	5220-0458
**Bacterial and virus strains**		
BL21(DE3)	Nippon gene	314-06533
**Chemicals, peptides, and recombinant proteins**		
dBET1	Angene International Ltd.	AG00ASDV
MG-132	Fujifilm Wako	135-18453
Bortezomib	Fujifilm Wako	021-18901
Epoxomicin	MedChemExpress	HY-13821
Protease inhibitor cocktails	Nacalai Tesque	25955-11
Penicillin–streptomycin solution (×100)	Fujifilm WAKO	168-23191
Lipofectamine LTX PLUS	Thermo Fisher Scientific	15338100
H-CGLWWPD-OH	This study, GL Biochem	
FITC-Ahx-LWWPD	This study, GL Biochem	
Ubiquitin	R&D systems	U-100H
UbcH5c/UBE2D3	R&D systems	E2-627-100
Critical commercial assays		
Cell counting kit-8	Dojindo	CK04
**Experimental models: cell lines**		
HeLa	RIKEN BRC	RCB0007
HEK293 HT-ELuc	73	
**Recombinant DNA**		
pFN21AB5414 (HaloTag-CREB1)	Kazusa DNA	FHC00339
pGEX4T-1-HaloTag	69	
pET-His_6_-TEV-hSTUB1-co*	This study, Vector builder	VB211011-1011chk
pGEX6P1-E1	Dr Y. Sato (Tottori Univ.)	
pCMV-HaloTag-flag-ELuc	73	
**Software and algorithms**		
SigmaPlot 15	Hulinks	
**Other**		
His Trap FF 1 mL	Cytiva	17525501
GSTrap FF 5 mL	Cytiva	17513102
HisTrap HP 5 mL	Cytiva	17524802
Glutathione Sepharose 4B	Cytiva	17075601
ResourceQ 1 mL	Cytiva	17117701
Ni Sepharose 6 ff	Cytiva	17531801
HisMag Sepharose Ni	Cytiva	289967388
4–20% Mini-PROTEAN TGX Precast Protein Gels	BIO-RAD	4561096
Chemi-Lumi One L	Nacalai Tesque	07880-54

### Biology

#### Protein expression vector cloning

##### His_6_-CHIP

The vector used to overexpress His_6_-CHIP in bacteria (pET-6xHis/TEV/hSTUB1[NM_005861.4](co)*) was constructed by VectorBuilder (Vector ID: VB211011-1011chk). The human STUB1 [NM_005861.4] ORF was codon-optimized and inserted into the pET vector.

#### Protein expression and purification

##### His_6_-CHIP

The His_6_-CHIP protein was expressed in *Escherichia coli* BL21(DE3) cells (Nippon Gene). BL21(DE3) cells were transformed using the pET-His_6_-TEV-hSTUB1-co* plasmid. Transformed cells were grown in LB medium until O.D. 600 reached 0.5–0.7 at 37 °C, and protein expression was induced with 0.1 mM isopropyl-β-d-thiogalactopyranoside (IPTG) for 18 h at 20 °C. Cell pellets were resuspended in lysis buffer (50 mM Tris–HCl at pH 8.1, 300 mM NaCl, and 1 mM phenylmethylsulfonyl fluoride (PMSF)). Cells were lysed by sonication (SFX150HH, BRANSON), and then the lysate was clarified by centrifugation at 26 900 × *g* (15 000 rpm) using himac-21 with an R24A rotor (Eppendorf) at 6 °C for 30 min. A 1 mL HisTrap column (Cytiva, Tokyo, Japan) was equilibrated in an NB1 buffer (50 mM Tris–HCl of pH 8.1, 300 mM NaCl, and 10 mM imidazole). The clarified lysate was then applied to a column. The column was washed extensively with NB1 and NW buffer (50 mM Tris–HCl of pH 8.1, 300 mM NaCl, and 30 mM imidazole). Proteins were eluted over stepwise gradients of 50, 100, 150, 200, 250, 300, 400, and 500 mM imidazole. The peak fractions were pooled and dialyzed with CHIP storage buffer [50 mM Tris–HCl (pH 8.1), 150 mM NaCl] using Amicon-Ultra-0.5 (10K MWCO) (#UFC501024, Merck, Germany), aliquoted and then stored at −80 °C. The concentration of the protein was determined with the molar absorption coefficient^88^*ε*_280_ = 102 050 M^−1^ cm^−1^. The absorbance at 280 nm was measured using a NanoDrop spectrophotometer (Thermo Fisher Scientific).

##### GST-UBE1-His_8_

For GST- and His-tagged human E1(UBE1), the expression vector (pGEX6P1-E1) was kindly gifted by Dr Yusuke Sato (Tottori University) and transformed into BL21(DE3) cells. Transformed cells were grown in LB medium with 0.1 mg mL^−1^ of ampicillin until O.D. 600 reached 0.5–0.7 at 37 °C, and protein expression was induced with 0.5 mM IPTG at 20 °C overnight. The collected cells were resuspended in an NB2 buffer (50 mM Tris–HCl pH 7.4, 150 mM NaCl, and 1 mM PMSF) and lysed by sonication. Lysate was clarified by centrifugation at 26 900 × *g* for 30 min at 6 °C. The clarified cell lysates were added to 5 mL of HisTrap HP (Cytiva). The column was washed with 8 column volume (CV) of the NB2 buffer. Then, the E1 protein was eluted over a 0–500 mM imidazole gradient in 10 CV with an NGC Quest chromatography system (BIO-RAD). The peak fractions were pooled and concentrated using Amicon-Ultra-0.5 (100 K MWCO) (#UFC510024, Merck, Germany), and then diluted 10 times with QB buffer (50 mM Tris–HCl pH 8.0). The sample was applied to ResourceQ 1 mL (Cytiva). The column was washed with 5 CV of QB buffer, and the E1 protein was eluted over a 0 to 1000 mM NaCl gradient. The peak fractions were pooled and concentrated using Amicon-Ultra-0.5 (Merk Millipore), diluted 10 times with NB2, and applied to 5 mL of HisTrapHP (Cytiva). The column was washed with 3 CV of NB2 buffer containing 0, 48, or 60 mM imidazole. E1 was eluted over 0–500 mM imidazole gradient. Purified protein was concentrated and buffer exchanged using Amicon-Ultra-0.5 (100 K MWCO) (Merck, Millipore) with an NB2 buffer. The concentration of the protein was determined with *ε*_280_ = 145 435 M^−1^ cm^−1^.

#### Fluorescence labeling

First, 40 µM of GST-HaloTag protein and 1 mM of fluorescein-5-maleimide (25 eq.) were incubated in a labeling buffer (20 mM NaPO_4_ of pH 6.5, 150 mM NaCl, and 5 mM EDTA) at r.t. for 2 h. Then, 1 M Tris–HCl (pH 6.8) was added to the reaction mixture to quench the reaction (final concentration 20 mM Tris–HCl). Excess fluorescent dye was removed by passing the pre-equilibrated Micro Bio-Spin 6 columns (#732-6221, BIO-RAD, CA) through PBS. The labeled protein (GST-HT-FAM) concentration and degree of labeling were calculated by measuring the absorbance of the labeled protein at 280 nm and 495 nm using NanoDrop.

Protein concentration.

*ε* = protein molar extinction coefficient (102 050 M^−1^ cm^−1^ for GST-HaloTag)*A*_max_ = *A*_495_
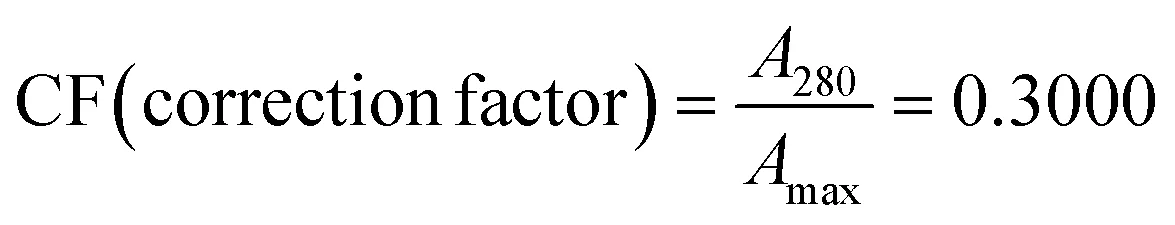
1
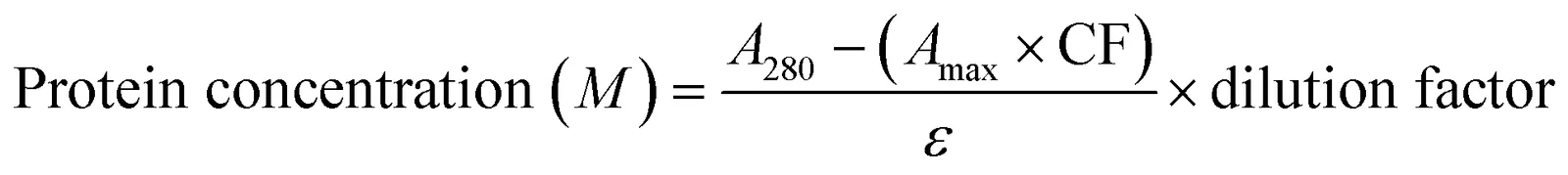


Calculate degree of labeling.

*ε*′ = molar extinction coefficient of fluorescein = 68 000 M^−1^ cm^−1^2
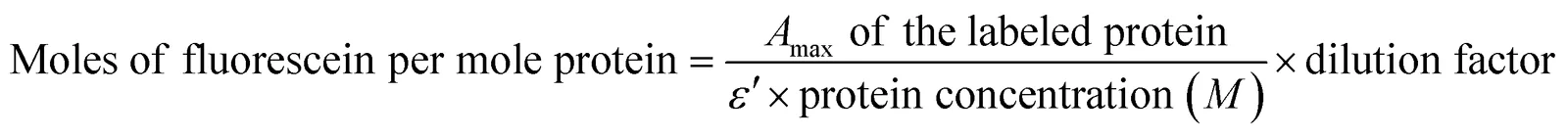


#### Cell culture

HeLa cells and HEK293 cells stably expressing HaloTag-conjugated Emerald Luciferase (HEK293-HT-ELuc) were cultured in Dulbecco's modified Eagle medium (DMEM) (#044-29765, Fujifilm WAKO, Osaka, Japan) containing 10% heat-inactivated fetal bovine serum (FBS) (#FB-1003/500, Biosera, France), penicillin and streptomycin (#26253-84, Nacalai Tesque, Kyoto, Japan) at 37 °C in a humidified atmosphere containing 5% CO_2_ in air. These cells were used in biological assays for 6–25 passages after thawing. For compound treatment with transient expression of HT-ELuc, HeLa cells were seeded at a density of 1.0 × 10^6^ cells per dish or well in 6 cm dishes or 6-well plates and cultured for 24 h. The cells were then transfected with an expression vector (pCMV-HaloTag-flag-ELuc) using Lipofectamine LTX PLUS transfection reagent (#15338100, Thermo Fisher Scientific, MA) for 4–6 h. Transfected cells were re-seeded at a density of 1.4 × 10^4^ cells per well in 96-well white plates (#655098; Greiner Bio-One, Austria) and cultured for 18 h. Then, the cells were treated with compounds in DMSO (final DMSO concentration, 0.1%). For cotreatment with proteasome inhibitors, final DMSO concentration was 0.2% at the indicated concentrations and time points. For compound treatment, stable HEK293-HT-Eluc cells were seeded at a density of 1.5 × 10^4^ cells per well in 96-well white plates and cultured for 24 h. The cells were then treated with compounds at the indicated concentrations and time points. Proteasome inhibitors (MG132, bortezomib, and epoxomicin) were applied 1 h prior to the compound addition.

For endogenous BRD4 proteins, HeLa cells were seeded at 1.0 × 10^5^ cells per well in a 24-well plate for 18 h. The cells were treated with the indicated concentrations of the compounds. The inhibitors were applied 1 h prior to the addition of compound 2. The cells were harvested and lysed using radioimmunoprecipitation assay (RIPA) buffer with SDS (#08714-04, Nacalai). After 15 min of incubation on ice, the lysate was sonicated to reduce the viscosity. Cell debris in the lysate was removed by centrifugation (Micro centrifuge Model 3740, Kubota, Japan) at 13 500 × *g* for 5 min at 4 °C. The total protein concentration in the supernatant was determined using a bicinchoninic acid (BCA) protein assay.

#### *In vitro* binding assay – FP assay

FP assays were performed in a 50 µL volume using black half-area 96-well plates (675086, Greiner) and read on an EnVision 2104 multilabel microplate reader (PerkinElmer) at rt.

#### Saturation binding

Saturation binding assay with 1 nM of tracer (FITC-Ahx-LWWPD) in DMSO and varying concentrations of His_6_-CHIP mixture was run in BA1 buffer (25 mM HEPES pH 7.5, 50 mM KCl, and 0.01% TritonX-100). Then, 25 µL of 2× tracer solution (2 nM FITC-Ahx-LWWPD in BA1 buffer) and 25 µL of 2× protein solution (His_6_-CHIP in BA1 buffer) were mixed in a well. The final DMSO concentration was 0.5%. The tracer-protein mixture was incubated at r.t. for 15 min, and the FP was read using a microplate reader (triplicate). The percentages of millipolarization (mP) were calculated with the mP value at the highest concentration of His_6_-CHIP (10^−5^ M) as 100% and that at the lowest concentration of His_6_-CHIP (10^−11^ M) as 0%. mP (%) data were plotted relative to log_10_[CHIP]M. Curve fitting to the sigmoidal dose–response model was performed using the SigmaPlot 15.0 software (Hulinks). *K*_d_ was extrapolated from EC_50_:3
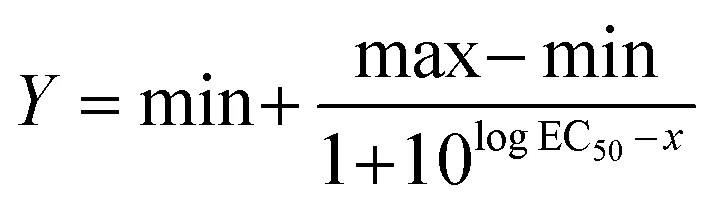


#### Competition binding assays

A 2× protein-tracer complex (2 nM Tracer + 2× EC_85_ His_6_-CHIP) was prepared in the BA1 buffer. A total of 2× varying concentrations of 1 were prepared in the BA buffer. Then, 25 µL of each solution was added to each well and incubated for 15 min at r.t. The final concentrations are as follows: His_6_-CHIP, 75 nM, tracer, 1 nM; and DMSO, 1.5%. The percentage of MP was calculated using 0 nM compound 1 as 100 nM and 0 nM His_6_-CHIP as 0. mP (%) data were plotted relative to log_10_[1] M. Curve fitting of the model for one-site competition was performed using SigmaPlot 15.0:4
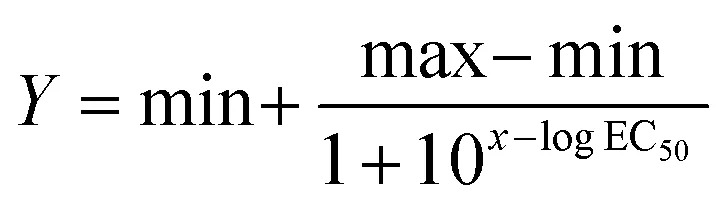


The inhibition constant (*K*_i_) was calculated using eqn (5) established by Nikolovska–Coleska *et al.*:^68^

5 *K*_i_ = [I]_50_/([L]_50_/*K*_d_ + [P]_0_/*K*_d_ + 1)

#### *In vitro* binding assay-pull down

##### GST-HT

Purified recombinant (1 µM) of GST-HaloTag protein and varying concentrations of 1 are incubated in the BA1 buffer [25 mM HEPES (pH 7.5), 50 mM KCl, 0.01% TritonX-100] at r.t. for 30 min. Then, 2 µM His_6_-CHIP was added and incubated for 30 min at r.t. The final DMSO concentration was 10%. The complex was captured by incubation with glutathione-Sepharose 4B (#17075601, Cytiva) at 4 °C. After washing the beads with BA1 buffer, the captured complex was eluted with BA1 buffer containing 10 mM of reduced-glutathione for 30 min at 4 °C. Eluted fractions were separated by SDS-PAGE using 4–20% gradient Mini-Protein TGX gel (#4561096, Bio-Rad) and stained with CBB.

##### BRD4

First, 0.2 µM of His_6_-CHIP and various concentrations of 2 were incubated in the BA2 buffer [100 mM Tris–HCl (pH 8.0), 150 mM NaCl, 5 mM EDTA and 10% (v/v) glycerol] at r.t. for 30 min, and then HeLa cell lysate in BA2 lysis buffer [10 mM Tris–HCl (pH 7.5), 150 mM NaCl, 0.5 mM EDTA, 1% (v/v) NP-40 and 10% (v/v) glycerol] containing a protease inhibitor cocktail (#25955-11, Nacalai Tesque) and 10 µM MG132 was added and incubated for 15 min on ice. The final DMSO concentration was 1.57%. The complex was captured by incubation with HisMag Sepharose Ni (#289967388, Cytiva) for 1 h at 4 °C. To prevent nonspecific binding, the reaction mixture contained 5 mM imidazole. After washing the beads with BA2 buffer containing 5 mM or 50 mM imidazole, the complex was eluted with BA2 buffer containing 500 mM imidazole for 30 min on ice. Eluted fractions were separated by SDS-PAGE using 7.5% miniprotein TGX gel (Bio-Rad), and the proteins were visualized by western blotting.

### *In vitro* ubiquitylation

Briefly, 1 was pre-incubated in 50 mM Tris–HCl (pH 8.1) buffer at r.t. for 30 min, and then 1 µM of GST-HT-FAM was added and incubated at r.t. for 30 min. After that, 625 nM His_6_-CHIP was added to compound 1 and the GST-HT-FAM mixture. The mixture was then incubated at r.t. for 30 min (solution B). Following that, 300 nM of GST-E1-His_8_, 625 nM of E2 (UbcH5c/UBE2D3) (#E2-627-100, R&D systems) and 30 µM of ubiquitin (Ub) (#U-100H, R&D systems) were preincubated at 37 °C for 30 min in Ub assay buffer [50 mM Tris–HCl, 5 mM ATP, 5 mM MgCl_2_, 0.6 mM DTT, pH 7.4] (solution A). The final DMSO concentration was 2.5%. Ubiquitylation was initiated by mixing Solutions A and B. After incubation for 1 to 4 h at 37 °C, the samples were placed on ice to stop the reaction. The products were separated by SDS-PAGE with 4–20% gradient Mini-Protein TGX gel (Bio-Rad) and analyzed by CBB staining and fluorescence gel imaging using a ChemiDoc touch imager (Bio-Rad). To analyze the Ub chain on GST-HT-FAM, we purified ubiquitylated and non-ubiquitylated GST-HT-FAM proteins from the reaction mixture by 2-step purification. In the first step, the tagged proteins (E1 and CHIP) were removed by passing the reaction mixture through Ni Sepharose 6 Fast Flow Beads (#17531801, Cytiva). In the second step, untagged proteins (E2 and free Ub) were removed by capturing GST-tagged proteins (ubiquitylated and non-ubiquitylated GST-HT-FAM) on glutathione-Sepharose 4 B beads. Ubiquitylated and non-ubiquitylated GST-HT-FAMs were separated by SDS-PAGE, transferred onto a PVDF membrane, and probed with anti-Ub (#10201-2-AP, Proteintech, IL, USA), K48 chain-specific (#ab140601, Abcam, UK), or K63 chain-specific (#05-1308, EMD Millipore, MA, USA) antibodies.

### Luciferase assay

Luciferase luminescence was measured as described previously.^73,75^ The viability of the compound-treated cells in a 96-well white wall microplate was assessed using the cell counting kit-8 (DOJINDO, Kumamoto, Japan). Then, 10 µL of CCK-8 solution was added to each well of 96-well plate. After 1 hour incubation at 37 °C in a 5% CO_2_ incubator, the absorbance at 450 nm was measured using an Envision 2104 multilabel microplate reader (PerkinElmer, MA). Subsequently, the medium was removed, and 50 µL of Lysis-Luc solution [100 mM 3-mercapto-1,2-propandiol, 30 mM tricine, 8 mM Mg(OAc)_2_, 200 µM EDTA, 1% (v/v) Triton X-100, 1.5 mM ATP, 500 µM coenzyme A trilithium salt (#13163-64, Nacalai Tesque), 250 µM sodium-d-Luciferin (#14682, Cayman Chemical Company, MI)] was added to each well. One minute later, luminescence was measured using an EnVision 2104 multilabel reader. Relative luminescence unit (RLU) values were calculated by dividing the luminescence intensity by the cell viability.

### Western blotting

Samples were mixed with 5× Laemmli buffer (0.25% [w/v] BPB [Bromophenol Blue], 0.5 M DTT, 50% [v/v] glycerol, 10% [w/v] SDS, 250 mM Tris–HCl, pH 6.8), and then, heated at 95 °C for 3 min. SDS-PAGE was performed by 4–20% or 7.5% mini-protease TGX gels (BIO-RAD). Proteins in the gel were transferred onto a PVDF membrane. After blocking with Bullet Blocking One (Nacalai Tesque), the membrane was washed 3 times with TBS-T buffer (20 mM Tris, 150 mM NaCl, 0.1% [v/v] Tween20, pH 7.6). Proteins on the membrane were probed with the following primary antibodies: anti-Ub antibody (#10201-2-AP, Protein Tech, 1:5000), anti-Ub K48 linkage-specific (#ab140601, Abcam, 1:2000), anti-Ub K63 linkage-specific (#05-1308, Merck, 1:2000), and anti-BRD4 (E2A7X) (#13440, Cell Signaling Technology, 1:2000). The membranes were then washed three times with TBS-T buffer. For chemiluminescence detection, the membranes were probed with the secondary antibody goat anti-rabbit IgG peroxidase conjugate (#5220-0458, SeraCare, 1:10 000). After washing three times with TBS-T buffer, the immunoblots were visualized using an ECL substrate (#07880-54, Nacalai Tesque). For western blotting, goat anti-rabbit IgG StarBright Blue 700 (#12004162, BIO-RAD, 1:5000) and Anti-Tubulin hFAB Rhodamine (#12004164, BIO-RAD, 1:5000) were used as secondary antibodies. Immunoblots were visualized using a ChemiDoc Touch (Bio-Rad) imaging system.

### Statistical analysis

All statistical calculations were performed using SigmaPlot 15.0 software (Hulinks).

## Author contributions

T. M.-O.: conceptualization, investigation, formal analysis, data curation, funding acquisition, writing – original draft. S. T.: supervision, writing – review and editing. E. H.: investigation and formal analysis. S. S.: writing – review and editing. K. O.: methodology, writing – review and editing, supervision. F. O.: project administration, supervision, writing – review and editing. M. I.: project administration, supervision, funding acquisition, writing – review and editing.

## Conflicts of interest

There are no conflicts to declare.

## Abbreviations

AcOEt: Ethyl acetate
BCA: Bicinchoninic acid assay
BCR-ABL: Breakpoint cluster region gene-Abelson murine leukemia viral oncogene
BPB: Bromophenol blue
BRD4: Bromodomain containing 4
CBB: Coomassie brilliant blue
CHCA: α-Cyano-4-hydroxyciannamic acid
CHIP: Carboxyl terminus of Hsp70 interacting protein
CPME: Cyclopentyl methyl ether
CRBN: Cereblon
CRL: Cullin RING ligases
CV: Column volume
DCAF1: DDB1 and CUL4 associated factor 1
DDB1: DNA damage-binding protein 1
DIPEA: *N*,*N*-Diisopropylethylamine
DMEM: Dulbecco's modified Eagle medium
E1: Ubiquitin activating enzyme
E2: Ubiquitin conjugating enzyme
E3: Ubiquitin ligase
E6-AP: Homologous to E6-AP carboxyl terminus domain
EDC: 1-(3-Dimethylaminopropyl)-3-ethylcarbodiimide
ELuc: Emerald luciferase
FA: Formic acid
FAM: Fluorescein
FBS: Fetal bovine serum
FITC: Fluorescein isothiocyanate
FP: Fluorescence polarization
GPC: Gel permeation chromatography
GST-HT: GST-tagged HaloTag protein
HATU: 3-Oxide hexafluorophosphate
HECT: Homologous to the E6AP carboxyl terminus
HER2: Human epidermal growth factor receptor 2
HH: Helical hairpin
HOBt: 1-Hydroxybenzotriazole
Hop: Hsp70–Hsp90 organizing protein
Hsc70: Heat shock cognate 70
IAP: Inhibitors of apoptosis proteins
IPTG: Isopropyl-β-d-thiogalactopyranoside
LB: Luria-Bertani medium
MDM2: Mouse double minute 2 homolog
mP: Millipolarization
NAE: NEDD8 activation enzyme
NEDD8: Neural precursor cell expressed developmentally down-regulated protein 8
P53: Tumor protein 53
PMSF: Phenylmethylsulfonyl fluoride
POI: Protein of interest
Poly-Ub: Poly ubiquitin
PROTAC: Proteolysis targeting chimera
PTLC: Preparative thin layer chromatography
PVDF: Polyvinylidene difluoride
RBR: RING–IBR–RING
RING: Really interesting new gene
RIPA: Radioimmunoprecipitation assay
RLU: Relative luminescence unit
STIP1: Stress induced phosphoprotein 1
STUB1: STIP1 homology and U-Box containing protein 1
TPD: Targeted protein degradation
TPR: Tetratricopeptide repeat
Ub: Ubiquitin
UPS: Ubiquitin-proteasome system
VHL: von Hippel-Lindau tumor suppressor.

## Supplementary Material

CB-007-D6CB00124F-s001

## Data Availability

All data generated or analysed during this study are included in this published article and its SI. Supplementary information including biological analyses, HPLC charts and MS spectra for compound 1 and 2, Supplementary method for cell permeability assay, and original gel images of SDS-PAGE analyses is available. See DOI: https://doi.org/10.1039/d6cb00124f.

## References

[cit1] Liu X., Ciulli A. (2023). ACS Cent. Sci..

[cit2] Sakamoto K. M., Kim K. B., Kumagai A., Mercurio F., Crews C. M., Deshaies R. J. (2001). Proc. Natl. Acad. Sci. U. S. A..

[cit3] Buckley D. L., Van Molle I., Gareiss P. C., Tae H. S., Michel J., Noblin D. J., Jorgensen W. L., Ciulli A., Crews C. M. (2012). J. Am. Chem. Soc..

[cit4] Itoh Y., Ishikawa M., Naito M., Hashimoto Y. (2010). J. Am. Chem. Soc..

[cit5] Ma Z., Zhou J. (2025). J. Med. Chem..

[cit6] Zhang L., Riley-Gillis B., Vijay P., Shen Y. (2019). Mol. Cancer Ther..

[cit7] Bird S., Pawlyn C. (2023). Blood.

[cit8] Hanzl A., Casement R., Imrichova H., Hughes S. J., Barone E., Testa A., Bauer S., Wright J., Brand M., Ciulli A., Winter G. E. (2023). Nat. Chem. Biol..

[cit9] Schröder M., Renatus M., Liang X., Meili F., Zoller T., Ferrand S., Gauter F., Li X., Sigoillot F., Gleim S., Stachyra T.-M., Thomas J. R., Begue D., Khoshouei M., Lefeuvre P., Andraos-Rey R., Chung B., Ma R., Pinch B., Hofmann A., Schirle M., Schmiedeberg N., Imbach P., Gorses D., Calkins K., Bauer-Probst B., Maschlej M., Niederst M., Maher R., Henault M., Alford J., Ahrne E., Tordella L., Hollingworth G., Thomä N. H., Vulpetti A., Radimerski T., Holzer P., Carbonneau S., Thoma C. R. (2024). Nat. Commun..

[cit10] Bondeson D. P., Smith B. E., Burslem G. M., Buhimschi A. D., Hines J., Jaime-Figueroa S., Wang J., Hamman B. D., Ishchenko A., Crews C. M. (2018). Cell Chem. Biol..

[cit11] Békés M., Langley D. R., Crews C. M. (2022). Nat. Rev. Drug Discovery.

[cit12] Winter G. E., Buckley D. L., Paulk J., Roberts J. M., Souza A., Dhe-Paganon S., Bradner J. E. (2015). Science.

[cit13] Zengerle M., Chan K.-H., Ciulli A. (2015). ACS Chem. Biol..

[cit14] Schneekloth A. R., Pucheault M., Tae H. S., Crews C. M. (2008). Bioorg. Med. Chem. Lett..

[cit15] Jevtić P., Haakonsen D. L., Rapé M. (2021). Cell Chem. Biol..

[cit16] Ishida T., Ciulli A. (2021). SLAS Discovery.

[cit17] Zhang X., Crowley V. M., Wucherpfennig T. G., Dix M. M., Cravatt B. F. (2019). Nat. Chem. Biol..

[cit18] Zeng J., Santos A. F., Mukadam A. S., Osswald M., Jacques D. A., Dickson C. F., McLaughlin S. H., Johnson C. M., Kiss L., Luptak J., Renner N., Vaysburd M., McEwan W. A., Morais-de-Sá E., Clift D., James L. C. (2021). Nat. Struct. Mol. Biol..

[cit19] Kim Y., Seo P., Jeon E., You I., Hwang K., Kim N., Tse J., Bae J., Choi H.-S., Hinshaw S. M., Gray N. S., Sim T. (2023). Cell Chem. Biol..

[cit20] Hickey C. M., Digianantonio K. M., Zimmermann K., Harbin A., Quinn C., Patel A., Gareiss P., Chapman A., Tiberi B., Dobrodziej J., Corradi J., Cacace A. M., Langley D. R., Békés M. (2024). Nat. Struct. Mol. Biol..

[cit21] Scott D. C., Dharuman S., Griffith E., Chai S. C., Ronnebaum J., King M. T., Tangallapally R., Lee C., Gee C. T., Yang L., Li Y., Loudon V. C., Lee H. W., Ochoada J., Miller D. J., Jayasinghe T., Paulo J. A., Elledge S. J., Harper J. W., Chen T., Lee R. E., Schulman B. A. (2024). Nat. Commun..

[cit22] Deshaies R. J., Joazeiro C. A. P. (2009). Annu. Rev. Biochem..

[cit23] Ballinger C. A., Connell P., Wu Y., Hu Z., Thompson L. J., Yin L.-Y., Patterson C. (1999). Mol. Cell. Biol..

[cit24] Zhang M., Windheim M., Roe S. M., Peggie M., Cohen P., Prodromou C., Pearl L. H. (2005). Mol. Cell.

[cit25] McDonough H., Patterson C. (2003). Cell Stress Chaperones.

[cit26] Jiang J., Ballinger C. A., Wu Y., Dai Q., Cyr D. M., Höhfeld J., Patterson C. (2001). J. Biol. Chem..

[cit27] Hatakeyama S., Yada M., Matsumoto M., Ishida N., Nakayama K.-I. (2001). J. Biol. Chem..

[cit28] Leak R. K. (2014). J. Cell Commun. Signaling.

[cit29] Chakraborty A. and Edkins A. L., in *The Networking of Chaperones by Co-Chaperone*s, ed. A. L. Edkins and G. L. Blatch, Springer International Publishing, Cham, 2023, pp. 351–387

[cit30] Cyr D. M., Höhfeld J., Patterson C. (2002). Trends Biochem. Sci..

[cit31] Wiederkehr T., Bukau B., Buchberger A. (2002). Curr. Biol..

[cit32] Alberti S., Demand J., Esser C., Emmerich N., Schild H., Höhfeld J. (2002). J. Biol. Chem..

[cit33] Murata S., Minami Y., Minami M., Chiba T., Tanaka K. (2001). EMBO Rep..

[cit34] Esser C., Scheffner M., Höhfeld J. (2005). J. Biol. Chem..

[cit35] Wang J., Zhao Q., Qi Q., Gu H., Rong J., Mu R., Zou M., Tao L., You Q., Guo Q. (2011). J. Cell. Biochem..

[cit36] Sisoula C., Trachana V., Patterson C., Gonos E. S. (2011). Free Radical Biol. Med..

[cit37] Muller P., Hrstka R., Coomber D., Lane D. P., Vojtesek B. (2008). Oncogene.

[cit38] Zhou P., Fernandes N., Dodge I. L., Reddi A. L., Rao N., Safran H., DiPetrillo T. A., Wazer D. E., Band V., Band H. (2003). J. Biol. Chem..

[cit39] Xu W., Marcu M., Yuan X., Mimnaugh E., Patterson C., Neckers L. (2002). Proc. Natl. Acad. Sci. U. S. A..

[cit40] Tsukahara F., Maru Y. (2010). Blood.

[cit41] Elliott E., Tsvetkov P., Ginzburg I. (2007). J. Biol. Chem..

[cit42] Dickey C. A., Kamal A., Lundgren K., Klosak N., Bailey R. M., Dunmore J., Ash P., Shoraka S., Zlatkovic J., Eckman C. B., Patterson C., Dickson D. W., Nahman N. S., Hutton M., Burrows F., Petrucelli L. (2007). J. Clin. Invest..

[cit43] Hatakeyama S., Matsumoto M., Kamura T., Murayama M., Chui D., Planel E., Takahashi R., Nakayama K. I., Takashima A. (2004). J. Neurochem..

[cit44] Petrucelli L. (2004). Hum. Mol. Genet..

[cit45] Shimura H., Schwartz D., Gygi S. P., Kosik K. S. (2004). J. Biol. Chem..

[cit46] Kalia L. V., Kalia S. K., Chau H., Lozano A. M., Hyman B. T., McLean P. J. (2011). PLoS One.

[cit47] Shin Y., Klucken J., Patterson C., Hyman B. T., McLean P. J. (2005). J. Biol. Chem..

[cit48] Chen L., Kong X., Fu J., Xu Y., Fang S., Hua P., Luo L., Yin Z. (2009). Cell. Immunol..

[cit49] Peng H.-M., Morishima Y., Jenkins G. J., Dunbar A. Y., Lau M., Patterson C., Pratt W. B., Osawa Y. (2004). J. Biol. Chem..

[cit50] Edkins A. L. , in The Networking of Chaperones by Co-chaperones: Control of Cellular Protein Homeostasis, ed. G. L. Blatch and A. L. Edkins, Springer International Publishing, Cham, 2015, pp. 219–242

[cit51] Kumar S., Basu M., Ghosh M. K. (2022). Genes Dis..

[cit52] Lucas S. C. C., Milbradt A. G., Breed J., De Genst E., Jackson A., Solovyeva A., Ackroyd B., Bauer M. R., Liu J., Longmire D., Petrović D., Rivers E. L., Stubbs C., Winlow P., Bazzaz S., Dickson P., Gikunju D., Guié M.-A., Guilinger J. P., Hupp C. D., Jetson R., Keefe A. D., Nugai K., Yeoman J. T. S., Zhang Y., Feng X., Yu D., Phillips C. (2025). ACS Med. Chem. Lett..

[cit53] Wang L., Kumar R., Winblad B., Pavlov P. F. (2024). Eur. J. Med. Chem..

[cit54] Kanack A. J., Olp M. D., Newsom O. J., Scaglione J. B., Gooden D. M., McMahon K., Smith B. C., Scaglione K. M. (2022). ChemBioChem.

[cit55] Choi W. H., Yun Y., Park S., Jeon J. H., Lee J., Lee J. H., Yang S.-A., Kim N.-K., Jung C. H., Kwon Y. T., Han D., Lim S. M., Lee M. J. (2020). Proc. Natl. Acad. Sci. U. S. A..

[cit56] Pabon N. A., Xia Y., Estabrooks S. K., Ye Z., Herbrand A. K., Süß E., Biondi R. M., Assimon V. A., Gestwicki J. E., Brodsky J. L., Camacho C. J., Bar-Joseph Z. (2018). PLoS Comput. Biol..

[cit57] Ravalin M., Theofilas P., Basu K., Opoku-Nsiah K. A., Assimon V. A., Medina-Cleghorn D., Chen Y.-F., Bohn M. F., Arkin M., Grinberg L. T., Craik C. S., Gestwicki J. E. (2019). Nat. Chem. Biol..

[cit58] Jr. Schneekloth J. S., Fonseca F. N., Koldobskiy M., Mandal A., Deshaies R., Sakamoto K., Crews C. M. (2004). J. Am. Chem. Soc..

[cit59] Zoppi V., Hughes S. J., Maniaci C., Testa A., Gmaschitz T., Wieshofer C., Koegl M., Riching K. M., Daniels D. L., Spallarossa A., Ciulli A. (2019). J. Med. Chem..

[cit60] Hsia O., Hinterndorfer M., Cowan A. D., Iso K., Ishida T., Sundaramoorthy R., Nakasone M. A., Imrichova H., Schätz C., Rukavina A., Husnjak K., Wegner M., Correa-Sáez A., Craigon C., Casement R., Maniaci C., Testa A., Kaulich M., Dikic I., Winter G. E., Ciulli A. (2024). Nature.

[cit61] Kaiho-Soma A., Akizuki Y., Igarashi K., Endo A., Shoda T., Kawase Y., Demizu Y., Naito M., Saeki Y., Tanaka K., Ohtake F. (2021). Mol. Cell.

[cit62] Ohtake F., Tsuchiya H., Saeki Y., Tanaka K. (2018). Proc. Natl. Acad. Sci. U. S. A..

[cit63] Chesser A., Pritchard S., Johnson G. V. (2013). Front. Neurol..

[cit64] Vasco Ferreira J., Rosa Soares A., Silva Ramalho J., Pereira P., Girao H. (2015). Sci. Rep..

[cit65] Tomoshige S., Naito M., Hashimoto Y., Ishikawa M. (2015). Org. Biomol. Chem..

[cit66] Tomoshige S., Hashimoto Y., Ishikawa M. (2016). Bioorg. Med. Chem..

[cit67] Los G. V., Encell L. P., McDougall M. G., Hartzell D. D., Karassina N., Zimprich C., Wood M. G., Learish R., Ohana R. F., Urh M., Simpson D., Mendez J., Zimmerman K., Otto P., Vidugiris G., Zhu J., Darzins A., Klaubert D. H., Bulleit R. F., Wood K. V. (2008). ACS Chem. Biol..

[cit68] Nikolovska-Coleska Z., Wang R., Fang X., Pan H., Tomita Y., Li P., Roller P. P., Krajewski K., Saito N. G., Stuckey J. A., Wang S. (2004). Anal. Biochem..

[cit69] Mishima Y., Yasuda S., Kunitomi A., Masuda-Ozawa T., Tomoshige S., Kato S., Kadono Y., Nakagawa K., Ishikawa M. (2025). Sci. Rep..

[cit70] Pettersson M., Crews C. M. (2019). Drug Discovery Today Technol..

[cit71] Soss S. E., Yue Y., Dhe-Paganon S., Chazin W. J. (2011). J. Biol. Chem..

[cit72] Kim H. T., Kim K. P., Lledias F., Kisselev A. F., Scaglione K. M., Skowyra D., Gygi S. P., Goldberg A. L. (2007). J. Biol. Chem..

[cit73] Tomoshige S., Komatsu F., Kikuchi T., Sugiyama M., Kawasaki Y., Ohgane K., Furuyama Y., Sato S., Ishikawa M., Kuramochi K. (2024). Bioorg. Med. Chem..

[cit74] Rosser M. F. N., Washburn E., Muchowski P. J., Patterson C., Cyr D. M. (2007). J. Biol. Chem..

[cit75] Ohgane K., Yoshioka H., Hashimoto Y. (2019). MethodsX.

[cit76] Belkina A. C., Denis G. V. (2012). Nat. Rev. Cancer.

[cit77] Gadd M. S., Testa A., Lucas X., Chan K.-H., Chen W., Lamont D. J., Zengerle M., Ciulli A. (2017). Nat. Chem. Biol..

[cit78] Crowe C., Nakasone M. A., Chandler S., Craigon C., Sathe G., Tatham M. H., Makukhin N., Hay R. T., Ciulli A. (2024). Sci. Adv..

[cit79] Ng S., Brueckner A. C., Bahmanjah S., Deng Q., Johnston J. M., Ge L., Duggal R., Habulihaz B., Barlock B., Ha S., Sadruddin A., Yeo C., Strickland C., Peier A., Henry B., Sherer E. C., Partridge A. W. (2022). J. Med. Chem..

[cit80] Yang Q., Zhao J., Chen D., Wang Y. (2021). Mol. Biomed..

[cit81] Soucy T. A., Smith P. G., Milhollen M. A., Berger A. J., Gavin J. M., Adhikari S., Brownell J. E., Burke K. E., Cardin D. P., Critchley S., Cullis C. A., Doucette A., Garnsey J. J., Gaulin J. L., Gershman R. E., Lublinsky A. R., McDonald A., Mizutani H., Narayanan U., Olhava E. J., Peluso S., Rezaei M., Sintchak M. D., Talreja T., Thomas M. P., Traore T., Vyskocil S., Weatherhead G. S., Yu J., Zhang J., Dick L. R., Claiborne C. F., Rolfe M., Bolen J. B., Langston S. P. (2009). Nature.

[cit82] Mathur D., Prakash S., Anand P., Kaur H., Agrawal P., Mehta A., Kumar R., Singh S., Raghava G. P. S. (2016). Sci. Rep..

[cit83] Nadel C. M., Ran X., Gestwicki J. E. (2020). Anal. Biochem..

[cit84] Baldwin A. D., Kiick K. L. (2011). Bioconjugate Chem..

[cit85] Höhfeld J., Cyr D. M., Patterson C. (2001). EMBO Rep..

[cit86] Ehrlich E. S., Wang T., Luo K., Xiao Z., Niewiadomska A. M., Martinez T., Xu W., Neckers L., Yu X.-F. (2009). Proc. Natl. Acad. Sci. U. S. A..

[cit87] Mishra A., Godavarthi S. K., Maheshwari M., Goswami A., Jana N. R. (2009). J. Biol. Chem..

[cit88] Pace C. N., Vajdos F., Fee L., Grimsley G., Gray T. (1995). Protein Sci..

