# Supplementary material for: Discovery of PROTACs recruiting the E3 ligase CHIP
Source: RSC Chem Biol. 2026 Sep 21;7(10):1997–2013. doi: 10.1039/d6cb00124f (PMC13592199; doi:10.1039/d6cb00124f)
Supplement: Supplementary file 1 [file CB-007-D6CB00124F-s001.pdf]

Supplementary Information (SI) for RSC Chemical Biology.
This journal is © The Royal Society of Chemistry 2026

Supporting Information

Discovery of PROTACs recruiting the E3 ligase CHIP

Tokiha Masuda-Ozawa[1], Shusuke Tomoshige[1*], Eisuke Hayakawa[1], Shinich Sato[1,2], Kenji Ohgane[3], Fumiaki Ohtake[4,5] and Minoru Ishikawa[1*]

1, Graduate School of Life Sciences, Tohoku University, 2-1-1 Katahira, Aoba-ku, Sendai 980-8577, Japan

2, Frontier Research Institute for Interdisciplinary Sciences, Tohoku University, 6-3 Aramaki aza-Aoba, Aoba-ku, Sendai 980-8578, Japan

3, Department of Chemistry, Ochanomizu University, 2-1-1 Otsuka, Bunkyo-ku, Tokyo 112-8610, Japan

4, Laboratory of Protein Degradation, Institute for Advanced Life Sciences, Hoshi University, 2-4-41 Ebara, Shinagawa-ku, Tokyo, Japan.

*Authors for correspondence

e-mail: minoru.ishikawa.e4@tohoku.ac.jp

Tel: +81-22-217-6197

e-mail: stomoshi@tohoku.ac.jp

Tel: +81-22-217-6198

Table of contents

1. **Figure S1**. Fluorescein-labeling and 2-step Ub-GST-HT purification.
2. **Figure S2**. *In vitro* ubiquitylation of GST-HT; PROTAC concentration dependency.
3. **Figure S3**. *In vitro* ubiquitylation of GST-HT; Time course.
4. **Figure S4.** Cell permeability of compound **1**.
5. **Figure S5.** Cell viability assay for compound **1** treated cells.
6. **Figure S6**. Reproducibility of **Figure 5a**
7. **Figure S7**. Degradation of BET family proteins induced by compound **2**.
8. **Figure S8**. Compound **2**-induced BRD4 degradation is abolished in CHIP-KO HeLa cells.

9. **Figure S9**. Analysis of BRD4 degradation mechanism.
10. **Figure S10**. HPLC spectra of compound **1** and **2**.
11. **Figure S11**. MS (MALDI-TOF) spectra of **1** and **2**.
12. **Supplementary method**.
13. **Original gel images**.

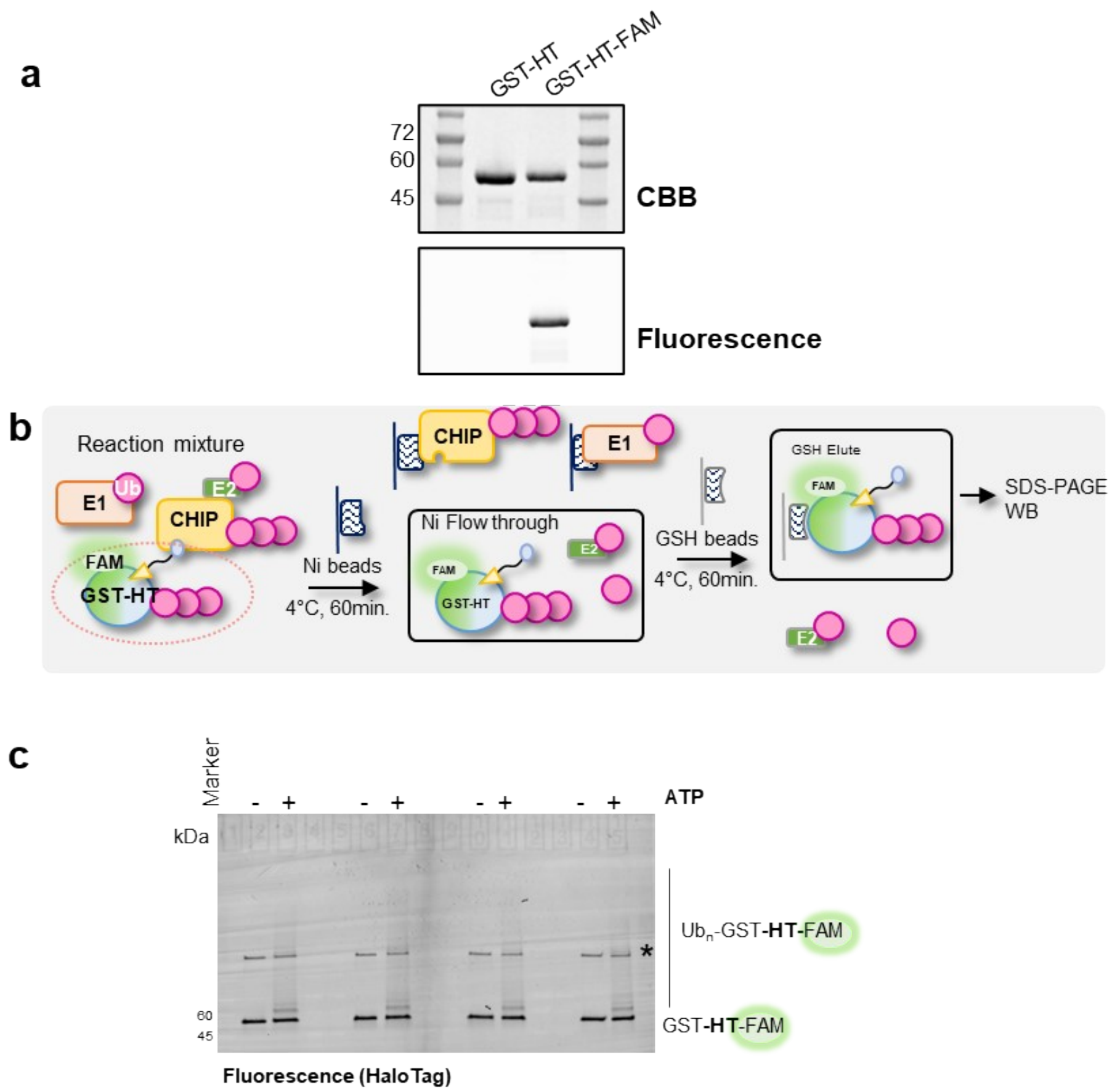

Figure S1. Fluorescein-labeling and 2-step Ub-GST-HT purification.
a, Images of GST-HT and GST-HT-FAM on sodium dodecyl sulfate-polyacrylamide gel electrophoresis (SDS-PAGE) The labeling efficiency was calculated from the absorbance at 280 and 295 nm. The estimated labeling efficiency was 3 fluorescent moles/protein moles. **b**, Schematic representation of the purification of ubiquitylated GST-HT-FAM after *in vitro* ubiquitylation. Ubiquitylated and non-ubiquitylated GST-HT-FAM were purified using Ni and glutathione (GSH) beads. His-tagged CHIP and E1 were captured on the Ni beads.

Untagged E2 and free ubiquitin were passed through GSH beads. **c**, Whole gel images of GST-HT-FAM and Ub_n-GST-HT-FAM. Each set of two lanes containing (-) and (+) ATP corresponds to the immunoblotting images for anti-Ub, anti-Ub (K48 linkage-specific), anti-Ub (K63 linkage-specific), and CBB in **Figure 2d**, respectively. A fluorescence image was captured before transfer to a PVDF membrane. Asterisks represent the GST-HT dimers.

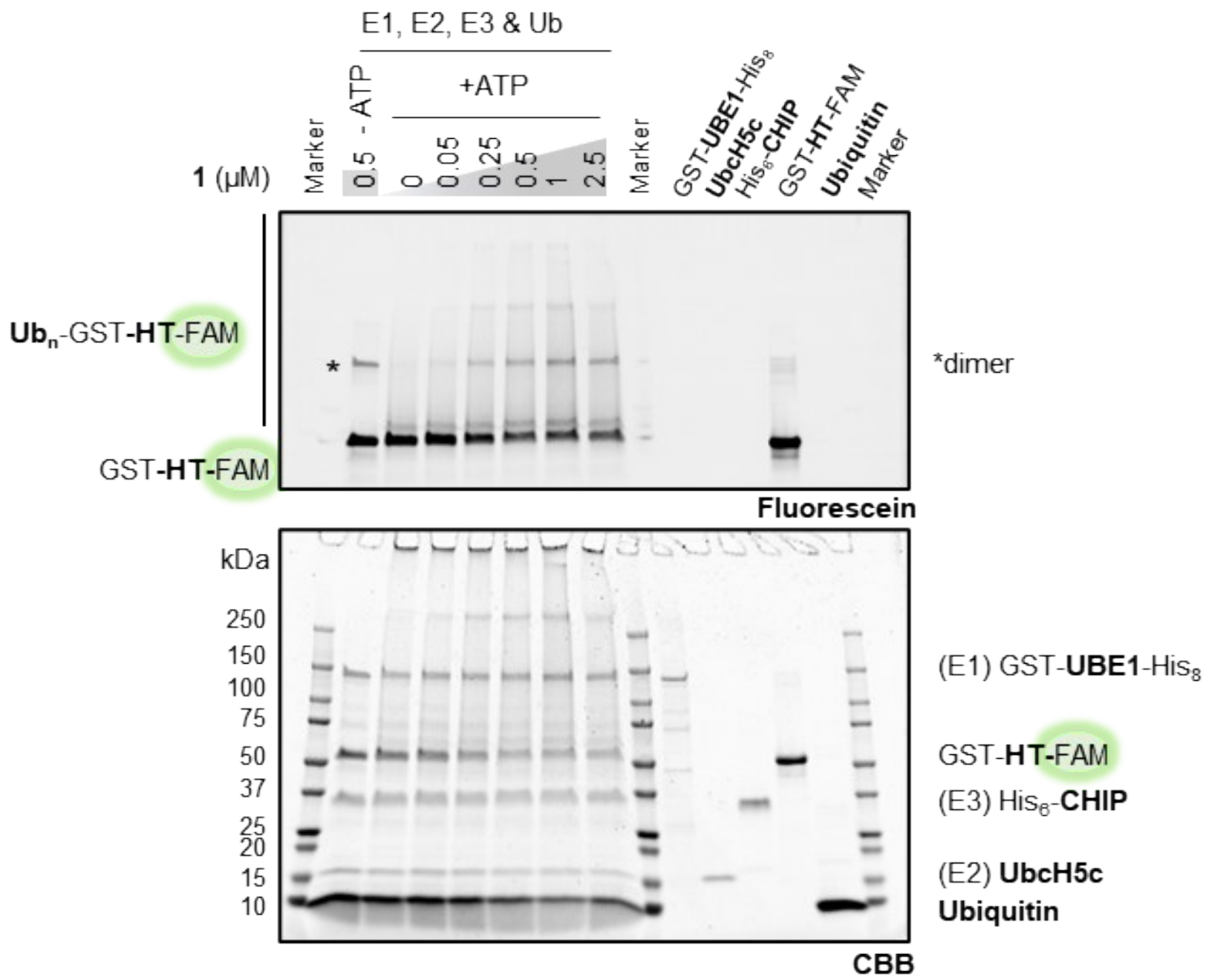

Figure S2. *In vitro* ubiquitylation of GST-HT; PROTAC concentration dependency. GST-HT-FAM, E1 (UBE1), E2 (UbcH5c), E3 (CHIP), and ubiquitin at various concentrations of **1** were mixed and incubated at 37 °C for 60 min. Proteins were separated by SDS-PAGE and visualized by CBB staining. The upper panel shows a fluorescence image of an SDS-PAGE gel. The lower panel shows a CBB-stained gel image. The expression of ubiquitylated GST-HT-FAM increased in a dose-dependent manner. The asterisk denotes a GST-HT dimer.

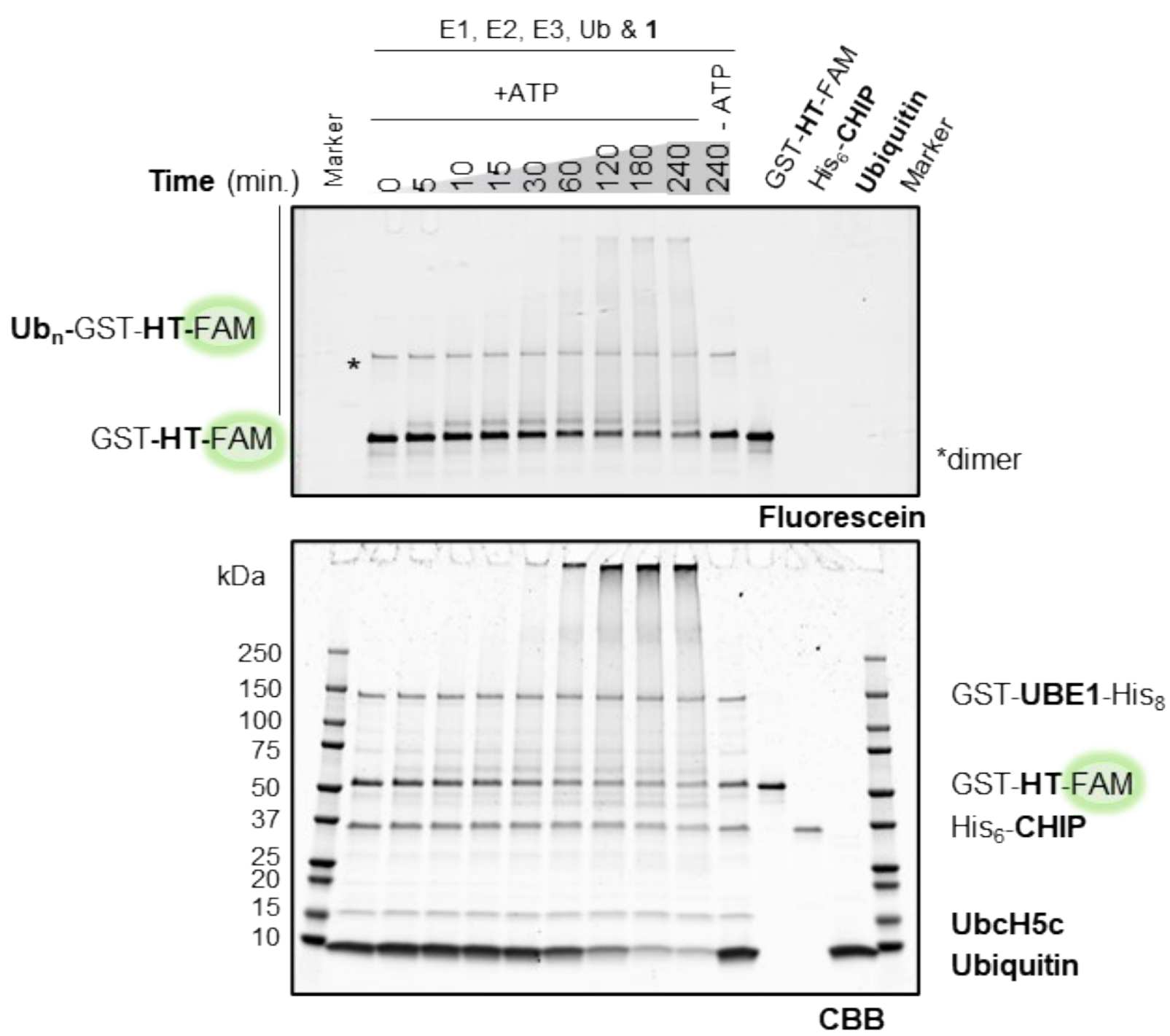

Figure S3. ***In vitro*** **ubiquitylation of GST-HT; Time course.**

1 (0.5 μM), GST-HT-FAM, E1 (UBE1), E2 (UbcH5c), E3 (CHIP) and ubiquitin were mixed and incubated at 37 °C for 0 to 240 minutes. Proteins were separated by SDS-PAGE and visualized by fluorescence gel imaging and CBB staining. The upper panel shows a fluorescence image of an SDS-PAGE gel. The lower panel shows a CBB-stained gel image. Smearing of GST-HT-FAM with higher molecular weights increased in a time-dependent manner. The asterisk denotes a GST-HT dimer.

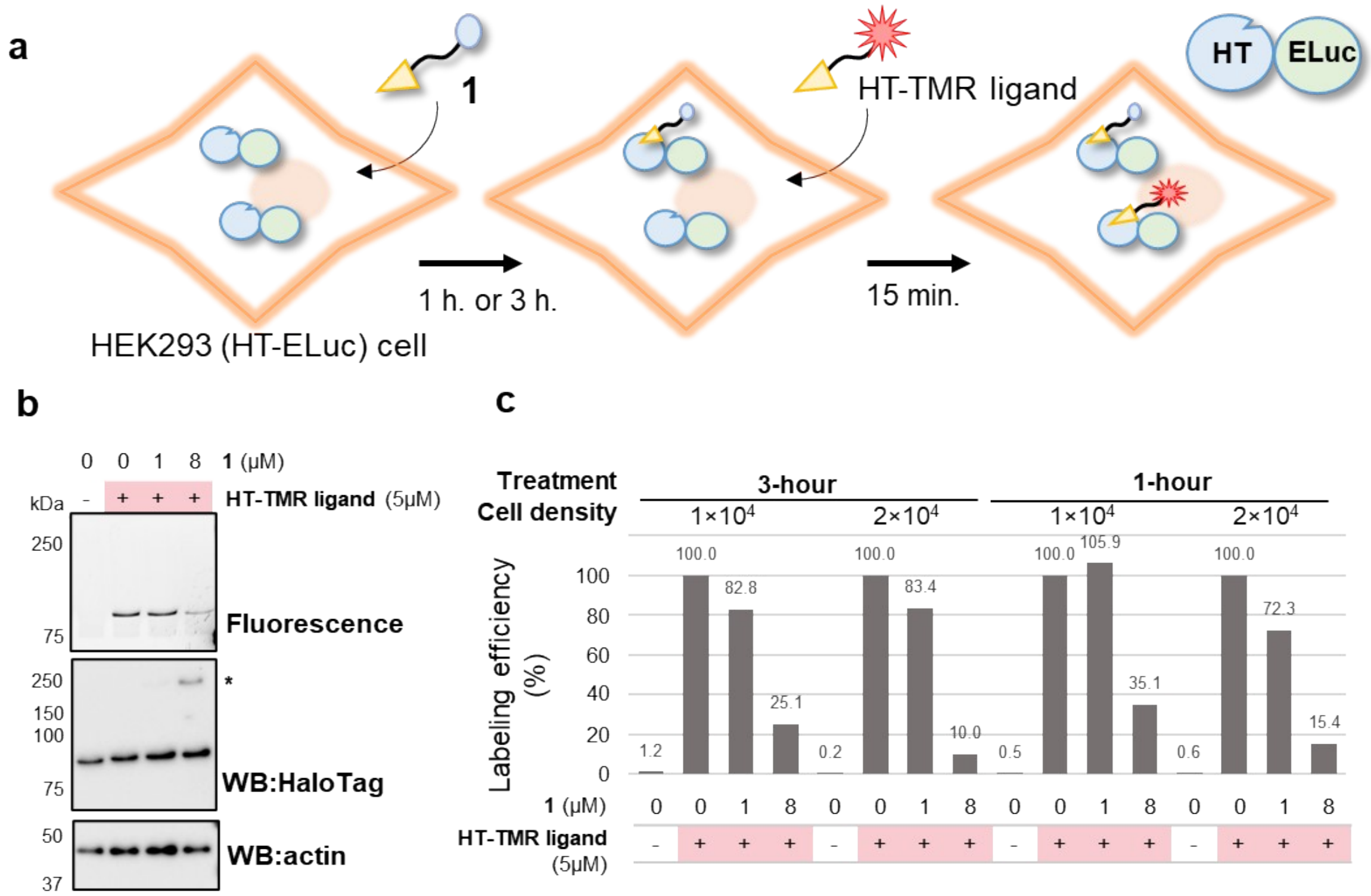

Figure S4. **Evaluation of the cell permeability of compound 1.**

a, Schematic representation of cell permeability assay. To estimate the cellular uptake of compound **1**, stably HT-ELuc expressing HEK293 cells were pre-treated with compound **1** for 1 or 3 hours, following HT-TMR ligand treatment. **b**, Representative images of fluorescence for TMR-labeled HT protein (top). Western blot analysis showing total HT protein level and loading control (actin) (bottom). Dada of 1-hour treatment of 1 in the 2×10^4 cells/well were shown. **c**, The efficiency of TMR labeling was calculated by using total band intensity of TMR fluorescence bands and anti-HT bands. Labeling efficiency (%) of the control (0 μM of **1** in the presence of HT-TMR ligand) was set as 100%. Data are from a single experiment. TMR labeling efficiency was dramatically inhibited in a **1** dose-dependent manner. The asterisk denotes the HT dimer.

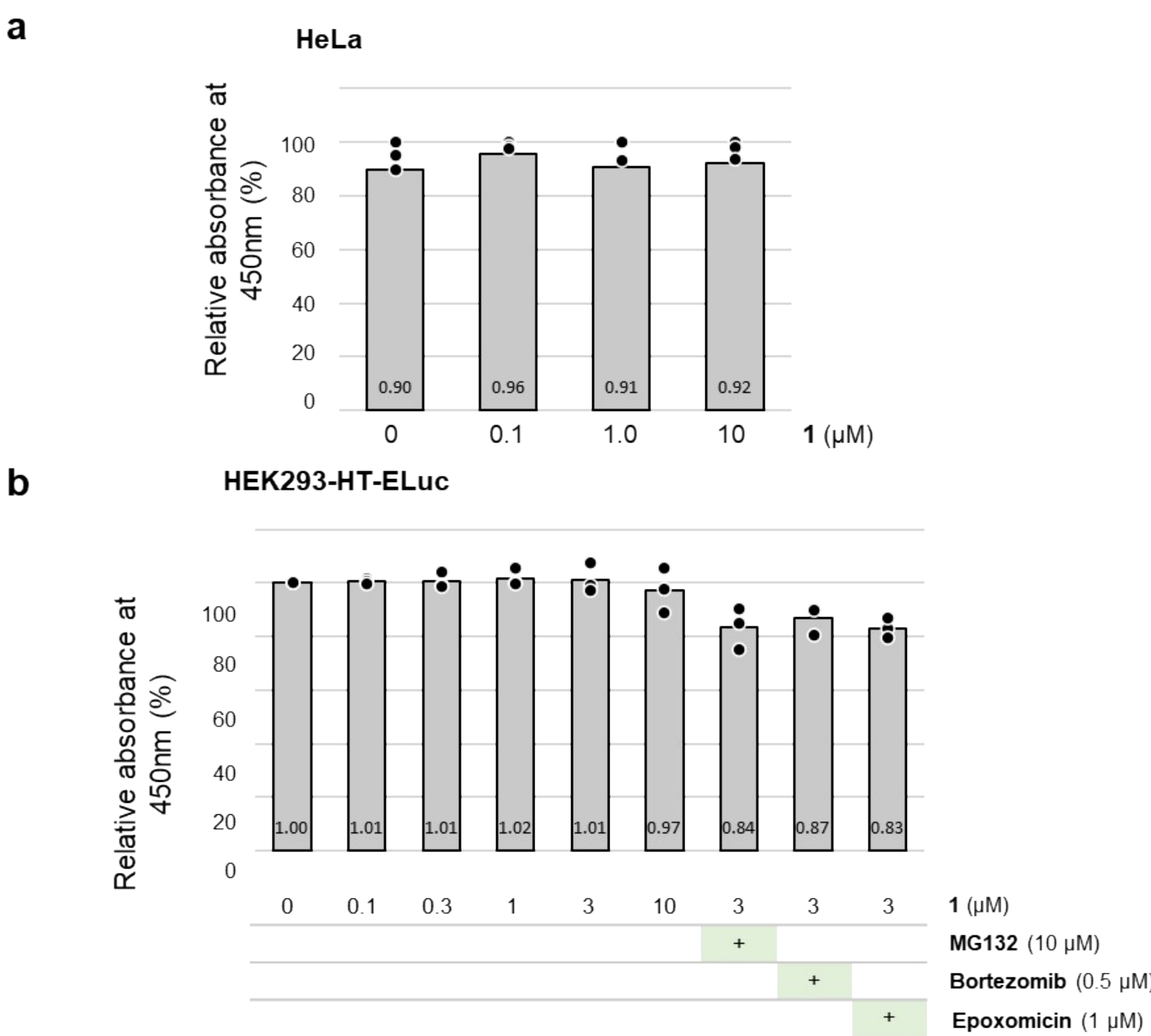

Figure S5. Cell viability of cells treated with compound 1.

The cell viability was measured using the WST-8 assay after a 6-hour treatment with compound **1**. Relative absorbance at 450 nm was normalized to the control (0 μM of **1**), which was set as 100%. The bar graphs represent the mean of three independent experiments, with individual data points overlaid as dots. No severe cytotoxicity was observed following treatment with **1** or proteasome inhibitors (MG132, bortezomib, and epoxomicin). **a,** HeLa cells transiently expressing HT-ELuc. **b,** HEK293 cells stably expressing HT-ELuc. These WST-8 datasets correspond to the cell viability measurements used for the normalization of the luciferase assays presented in **Figure 4a** and **4b**, respectively.

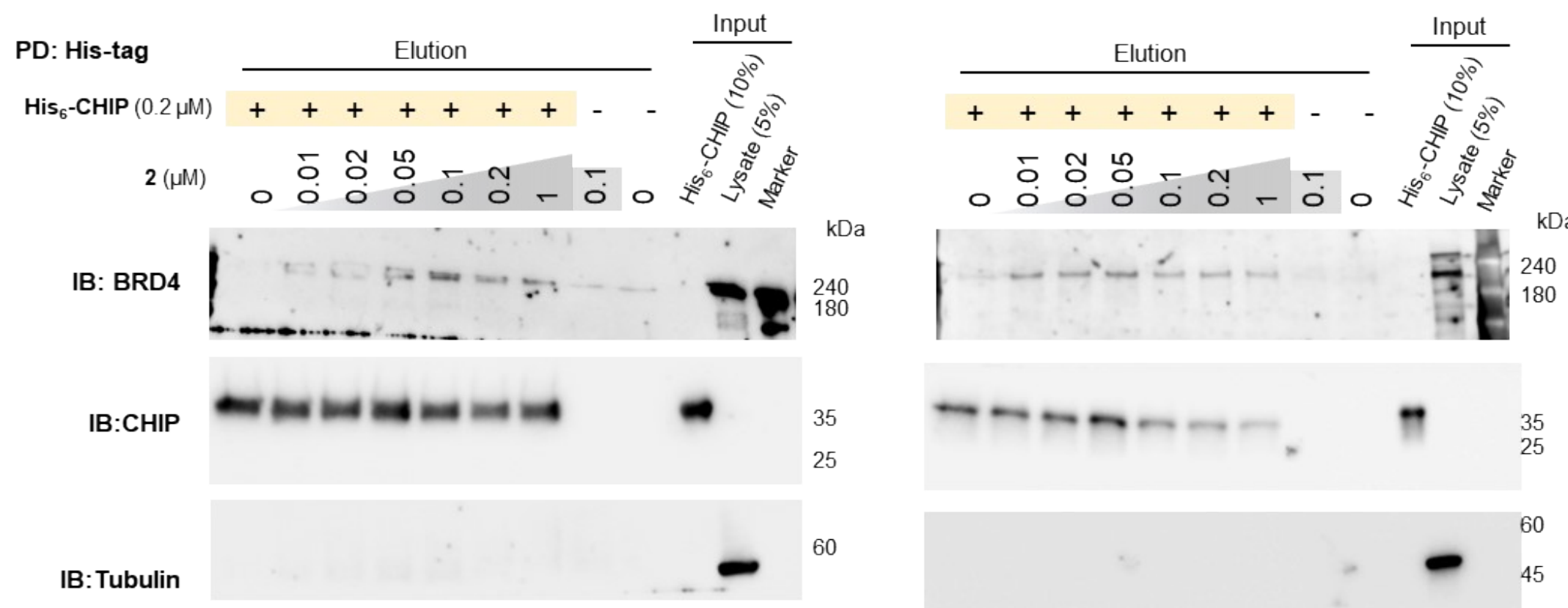

Figure S6. Reproducibility of *in vitro* ternary complex formation in Figure5a.

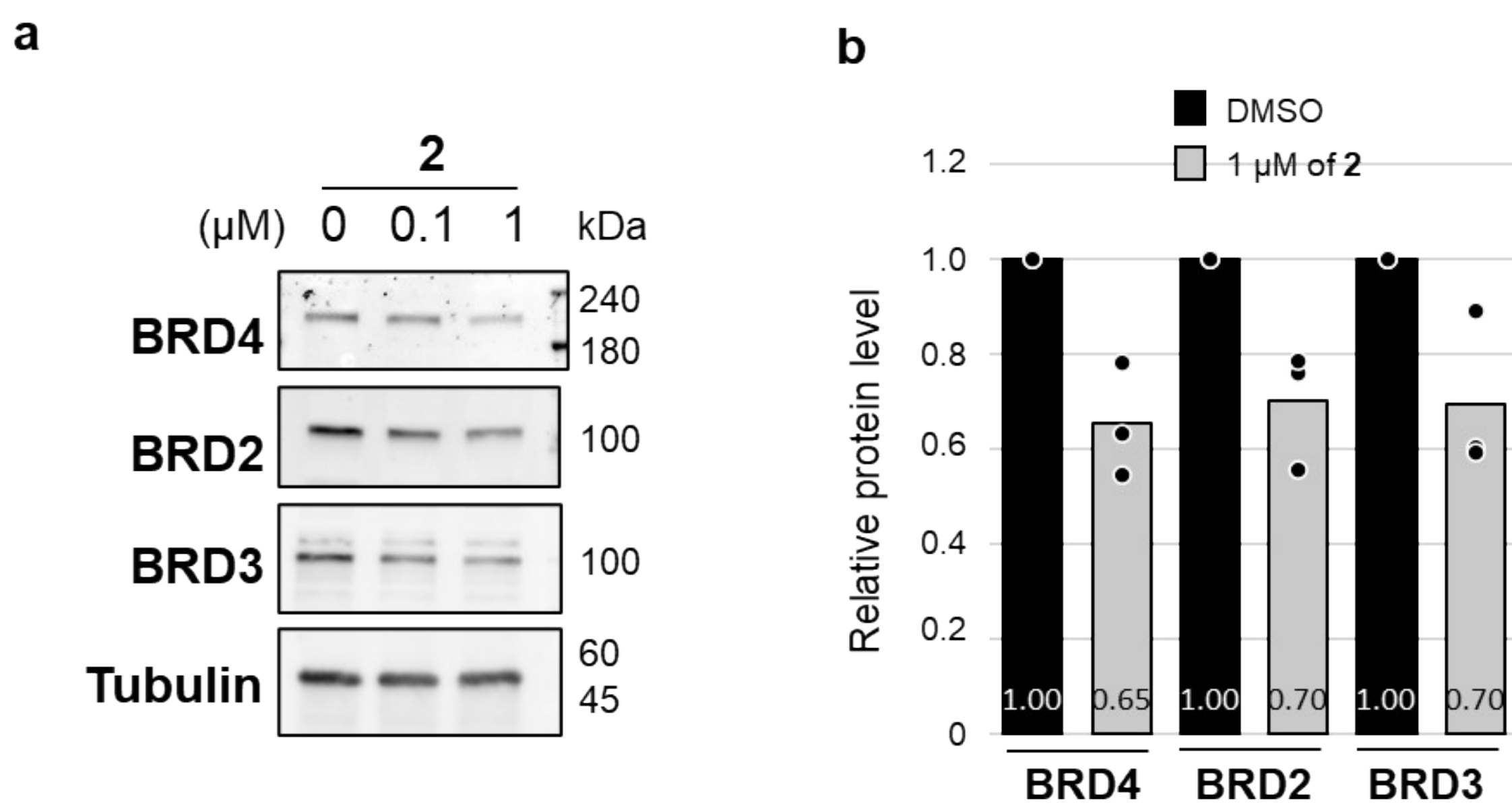

Figure S7. Degradation of BET family proteins induced by compound 2.
a, Representative western blots showing BRD4, BRD2, and BRD3 levels in HeLa cells treated with the indicated concentrations of compound **2** for 24h. **b**, Quantitative analysis of BRD4, BRD2, and BRD3 degradation. Western blot band intensities of BRD4, BRD2, and BRD3 were normalized to that of tubulin. Relative intensities of DMSO control were set to 1. Bars and numerical values at the bottom of the graphs represent the mean of three independent experiments. Individual data points are shown as dots.

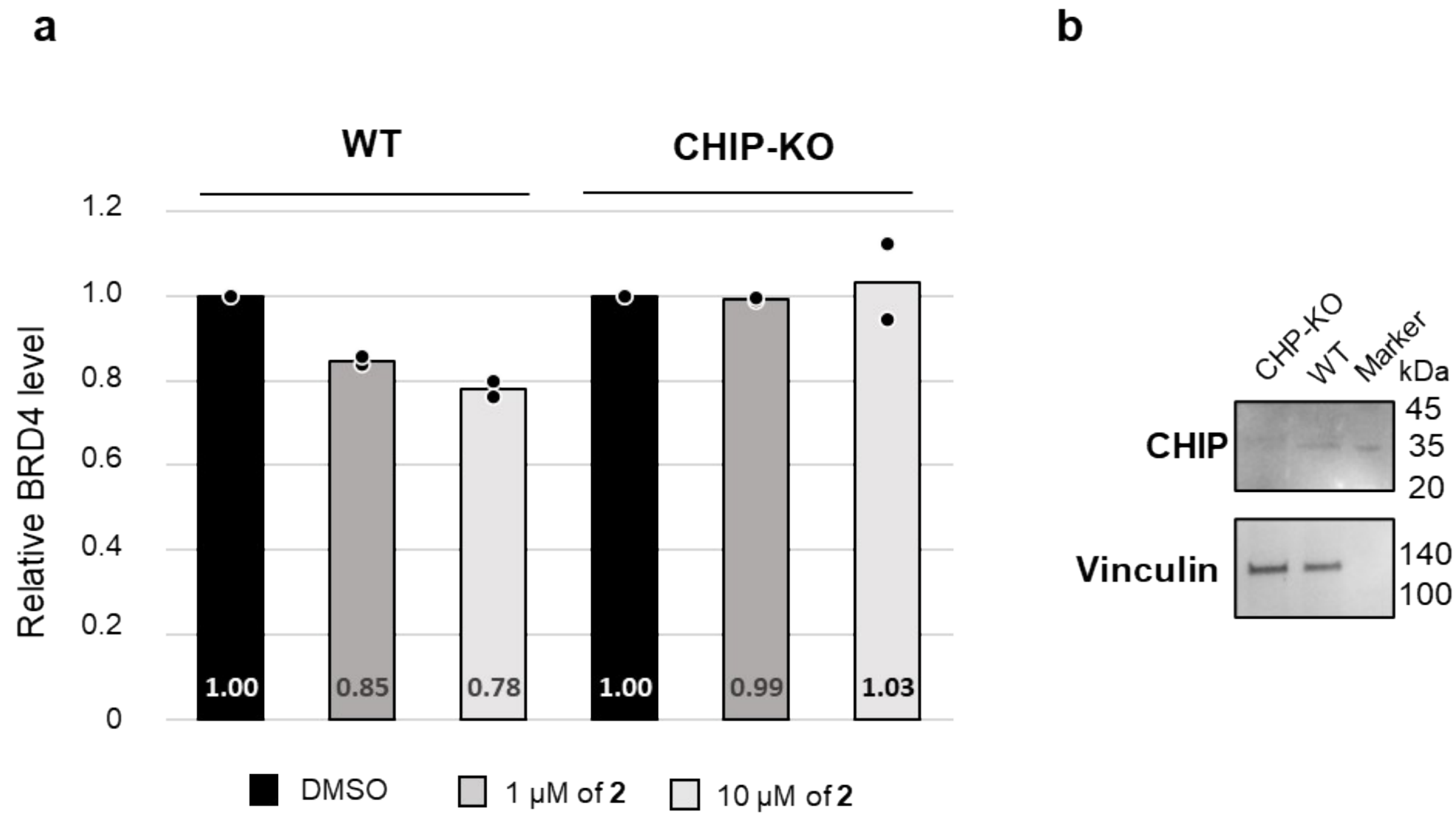

Figure S8. Compound 2-induced BRD4 degradation is abolished in CHIP-KO HeLa cells.

a. Quantitative analysis of BRD4 protein levels in wild-type (WT) and CHIP-knockout (CHIP-KO) HeLa cells treated with the indicated concentrations of compound **2** for 24h. Western blot band intensities of BRD4 were normalized to that of tubulin. Relative intensities of DMSO control were set to 1. Bars and numerical values at the bottom of the graph represent the mean of two independent experiments. Individual data points are shown as dots. **b**. Western blot analysis of CHIP (37 kDa) expression in WT and CHIP-KO HeLa cell lysates. Vinculin was used as a loading control.

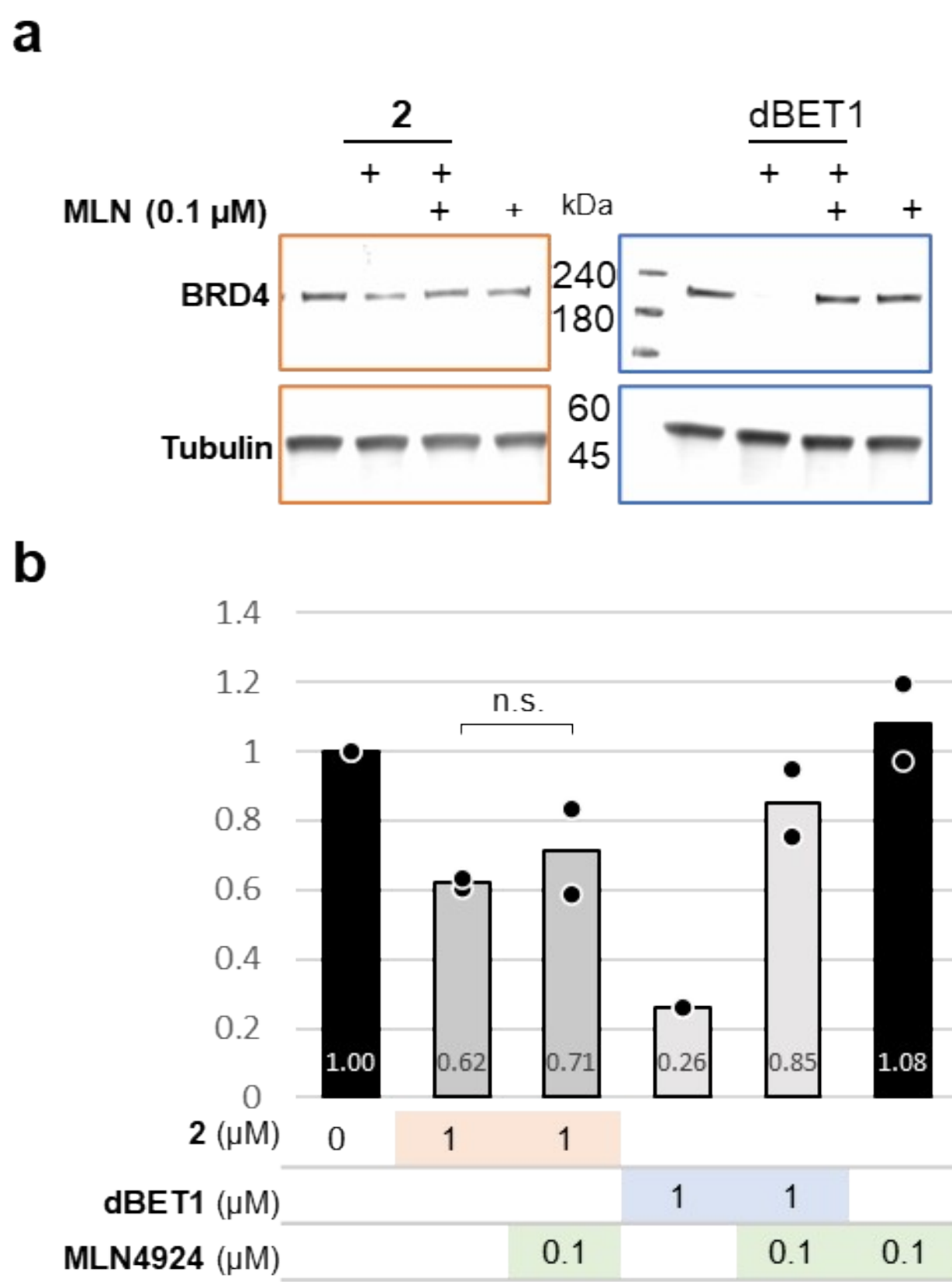

Figure S9. Analysis of BRD4 degradation mechanism.

a-b, BRD4 degradation in the presence of MLN4924. **a**, Representative western blot images from two repeats. BRD4 expression HeLa cells were treated with Compound **2** for 6 h. 0.1 µM of inhibitor was added to cells 1-hour prior to addition of **2** (1 µM). **b**, Quantitative analysis of the relative intensity of BRD4. The intensity of BRD4 was normalized to that of tubulin. Intensity of DMSO control (0 µM) was set as 1. The bar graph shows the mean relative intensity from two independent repeats. Each data point is plotted as a dot. Although the degradation induced by dBET1 was strongly inhibited by MLN4924, degradation induced by **2** was not significantly altered. Statistical difference between 1 µM of **2** and 1 µM of **2** + 0.1 µM of MLN4924 were analyzed by paired t-test (two-tailed). n.s. stands for "not statistically significant" ($p \geq 0.05$).

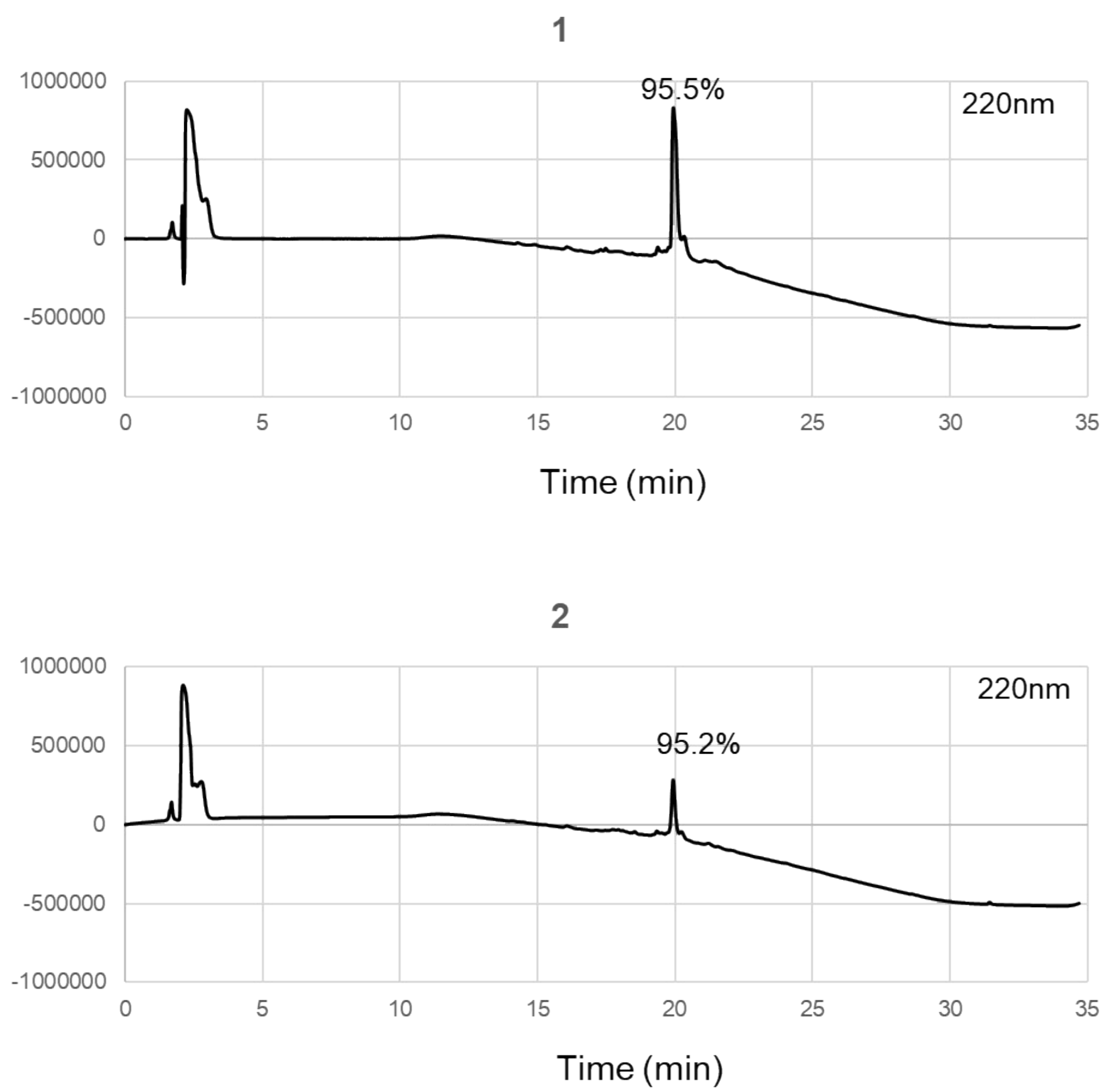

Figure S10. HPLC spectra of 1 and 2.

The assayed compounds were at least 95% pure as determined by HPLC. The peak area ratio for each assay is shown. The HPLC analysis conditions were as follows: mobile phase, 0.1% formic acid in H_2O and mobile phase, acetonitrile. UV signals were monitored at 220 nm for 35 min.

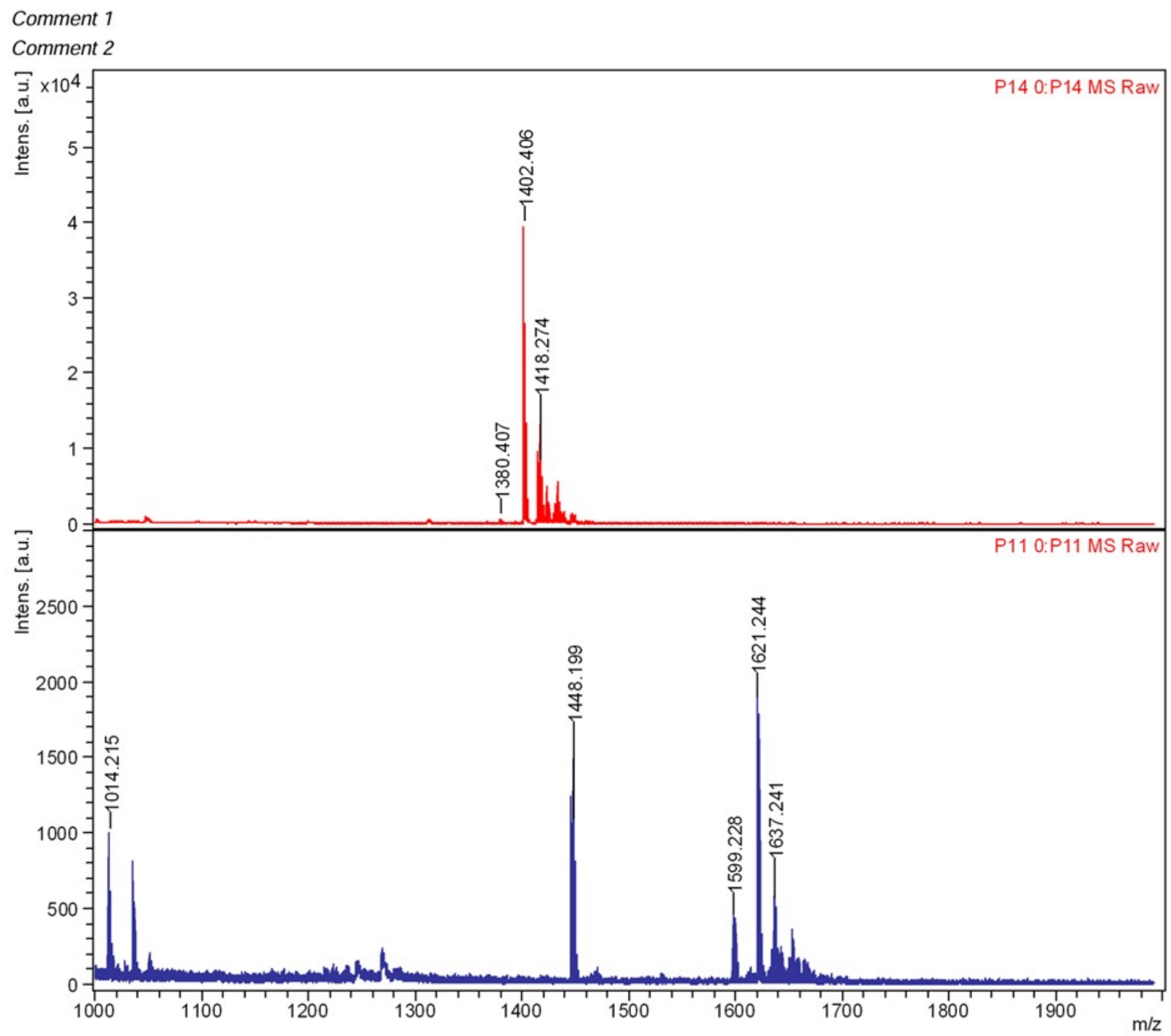

Figure S11. **MS (MALDI-TOF) spectrum of compound 1 and 2.**

Supplementary method

Cell Permeability Assay

HEK293-HT-ELuc cells were seeded at a density of 1×10^4 or 2×10^4 cells/well in 96-well white plates. After 24 h of incubation, the cells were treated with 0, 1, or 8 μM of **1**. After 1 or 3 h of treatment with **1**, the cells were incubated with 5 μM HaloTag-TMR ligand (#8252, Promega, WI) for 15 min at 37 °C in a 5% CO_2 incubator. To remove non-internalized compounds, the medium was removed and the cells were washed twice with PBS. Fresh medium was added, and the cells were further incubated at 37 °C for 30 min to allow for the washout of unbound ligands.

The cells were then washed with PBS and resuspended in ice-cold lysis buffer [50 mM Tris-HCl (pH 7.4), 150 mM NaCl, 1 mM EDTA, 0.5% Triton X-100, and a protease inhibitor cocktail (#25955-11, Nacalai Tesque)], followed by incubation on ice for 30 min. The lysates were clarified by centrifugation at 20,000 × g for 5 min at 4 °C. Total protein concentrations were determined by BCA assay. To visualize TMR-labeled HT-ELuc, the proteins were separated by SDS-PAGE, and fluorescence gel images were captured using a ChemiDoc Touch MP imager (Bio-Rad, CA).

Subsequently, the proteins were transferred from the SDS-PAGE gel onto a PVDF membrane and probed with the following primary antibodies: anti-HaloTag pAb (#G9281, Promega, 1:1,000) and anti-β-actin (ACTB) (#A5316, Sigma-Aldrich, 1:3,000). The membrane was then incubated with the corresponding secondary antibodies: anti-rabbit IgG polyclonal antibody (#NBP1-75297, Novus Biologicals, 1:3,000) or anti-mouse IgG antibody, HRP conjugate (#123-49, Merck, 1:2,000). Immunoreactive bands were visualized using an ECL substrate (#07880-54, Nacalai Tesque).

CHIP/STUB1 knockout cell line

HeLa cells were co-transfected with CHIP CRISPR/Cas9 KO plasmid (h2) and CHIP HDR plasmid (h2) (#sc-400815-KO-2 and #sc-400815-HDR-2, respectively, Santa Cruz Biotechnology) using lipofectamine LTX PLUS (Invitrogen). Successfully transfected clones were selected using 1.0 or 1.25 μg/ml of puromycin (#19752-64, Nacalai Tesque, CAS:58-58-2). To isolated single clones, cells were seeded by limiting dilution in 96-well plates (0.8 cells/well). Expanded monoclonal cells were used for further experiments. CHIP-knockout cell clones were validated by western blotting.

Western blotting

BRD2, BRD3, and CHIP proteins were detected by using anti-BRD2 (D8924) (#5848, Cell Signaling Technology, 1:2,000), anti-BRD3 (#11859-1-AP, Proteintech, 1:1,000), and anti-CHIP (G-2) (#sc-133066, Santa Cruz Biotechnology, 1:500) as primary antibodies. The membranes were probed with the secondary antibody, goat anti-rabbit IgG peroxidase conjugate (#5220-0458, SeraCare, 1:10,000) or goat anti-mouse IgG HRP conjugate (#12-349, Merck, 1:2,000). Proteins bands were visualized using ECL substrates (#07880-54, Nacalai Tesque or # 296-69901, Wako Pure Chemical Industries).

Original gel images

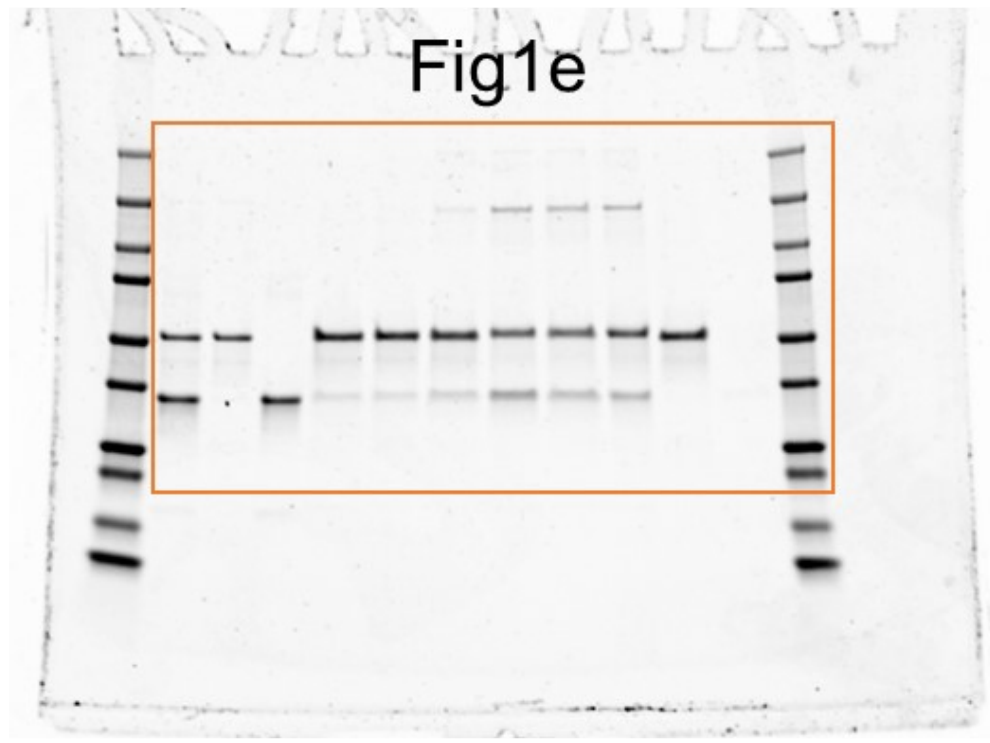

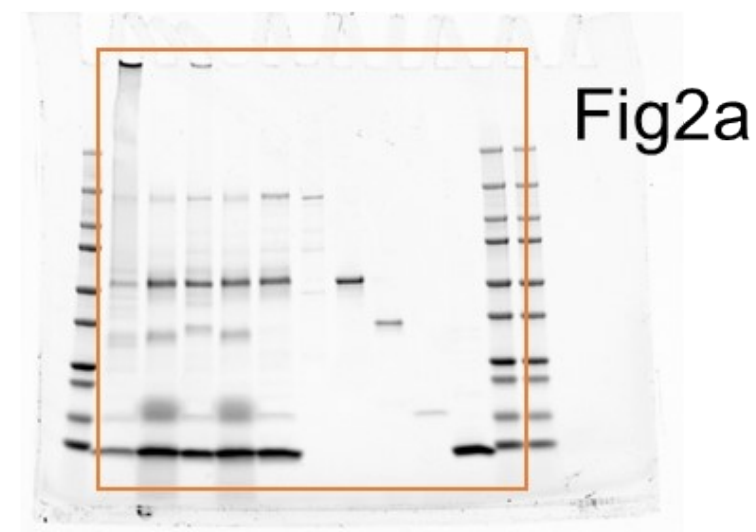

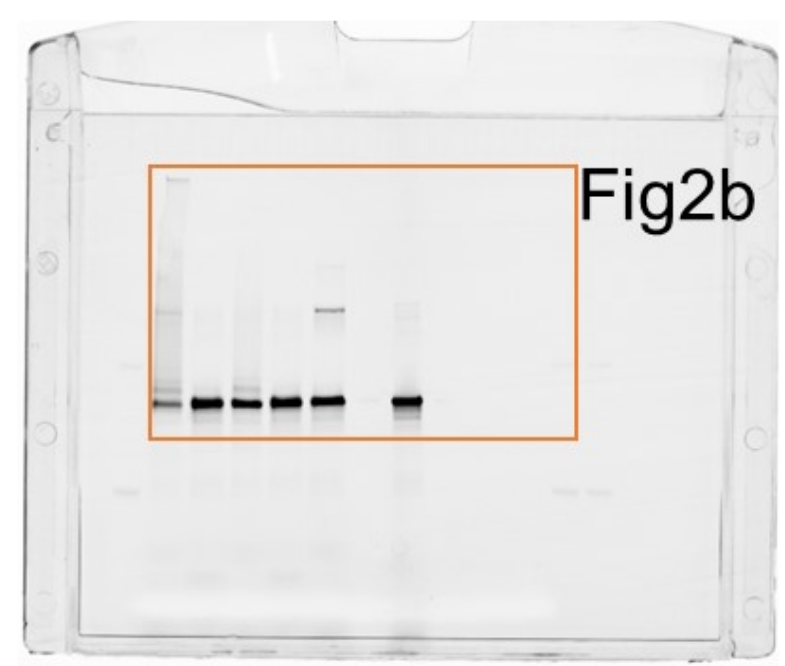

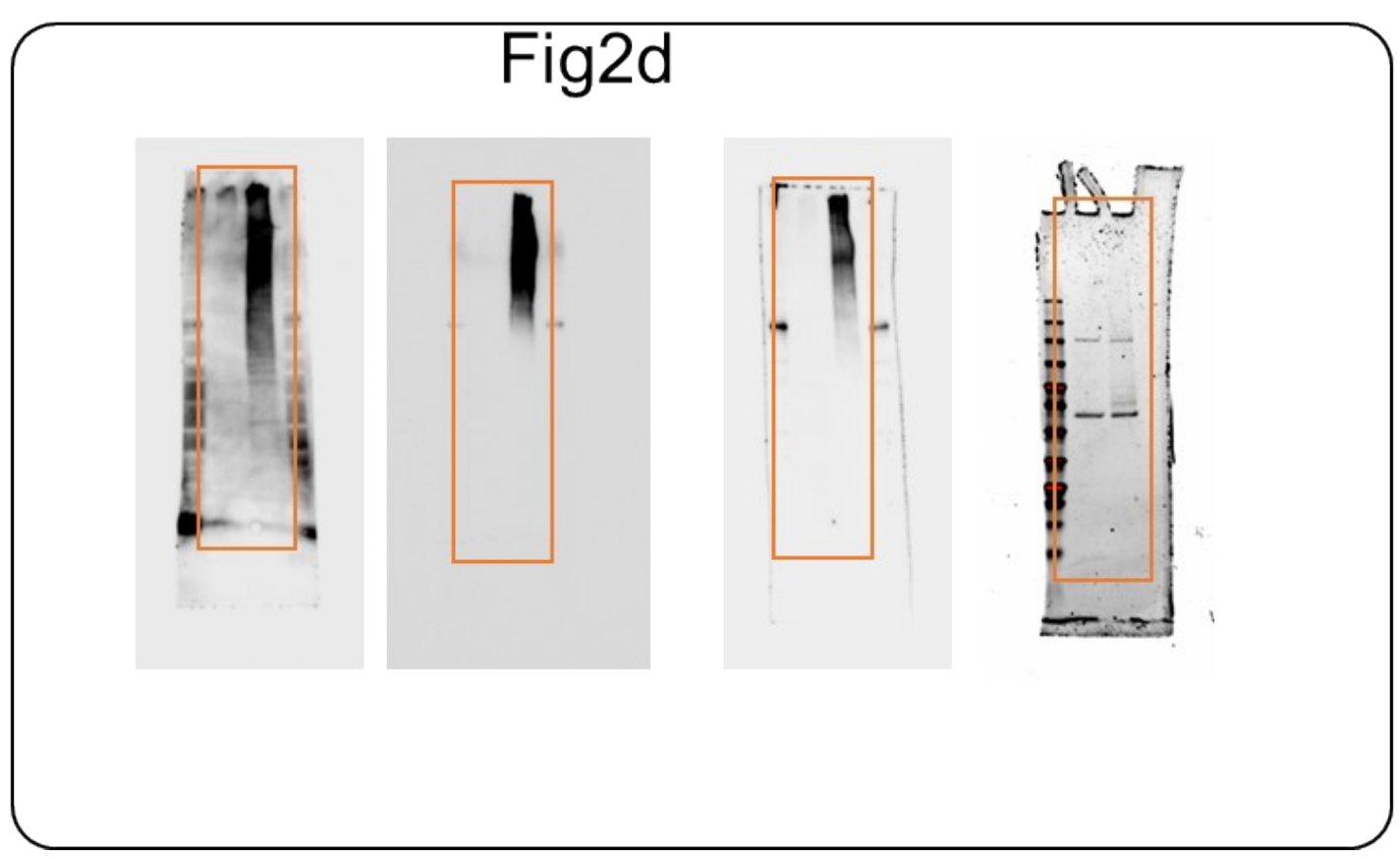

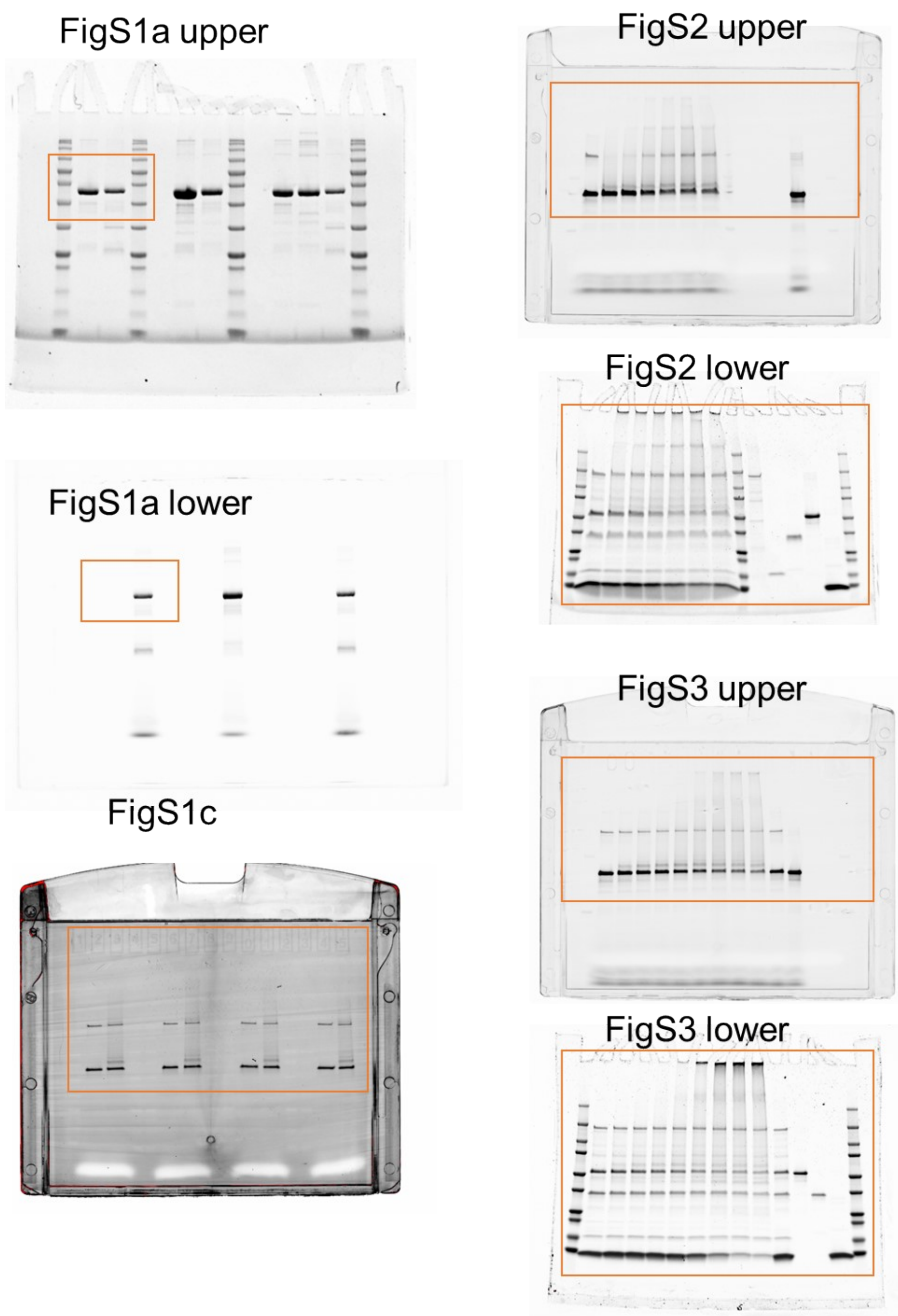
FigS1a upper
FigS1a lower
FigS1c
FigS2 upper
FigS2 lower
FigS3 upper
FigS3 lower

Fig5a

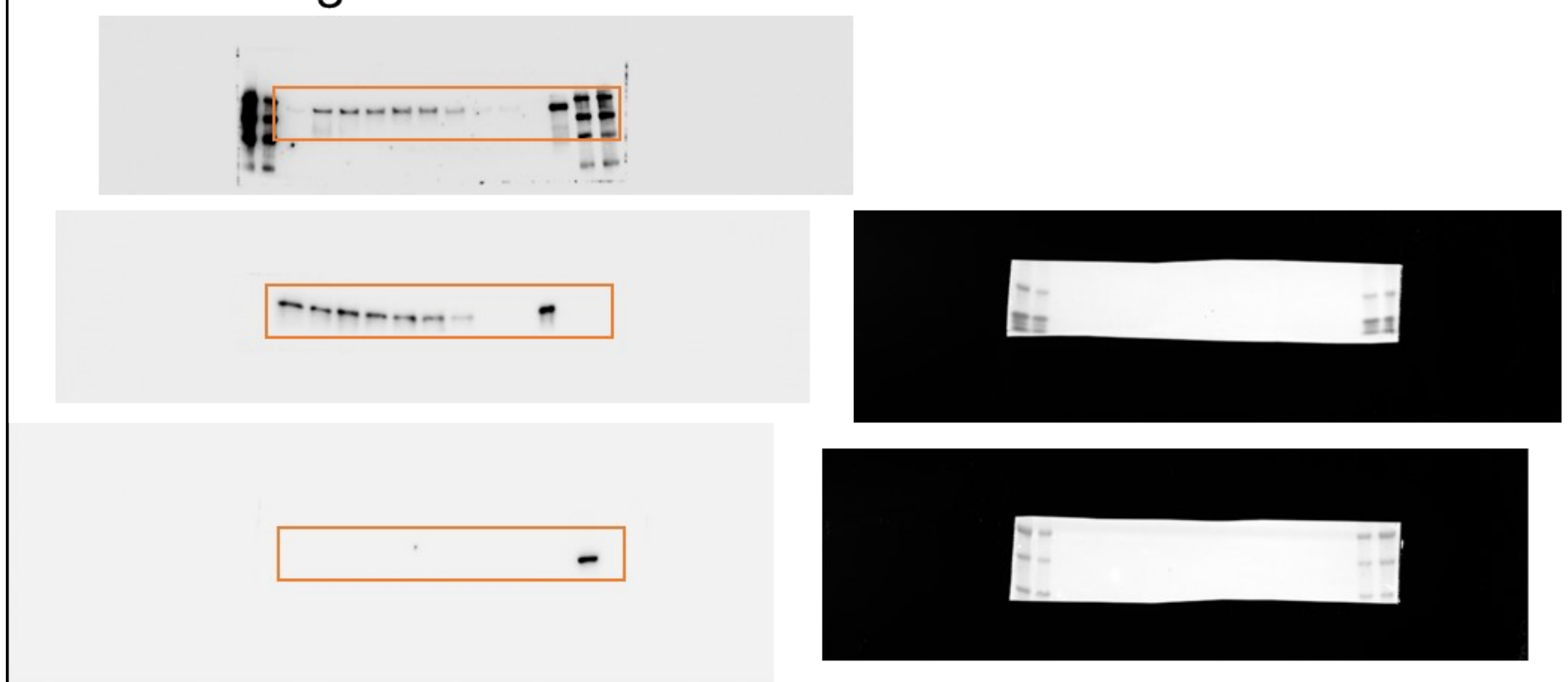

Fig5b

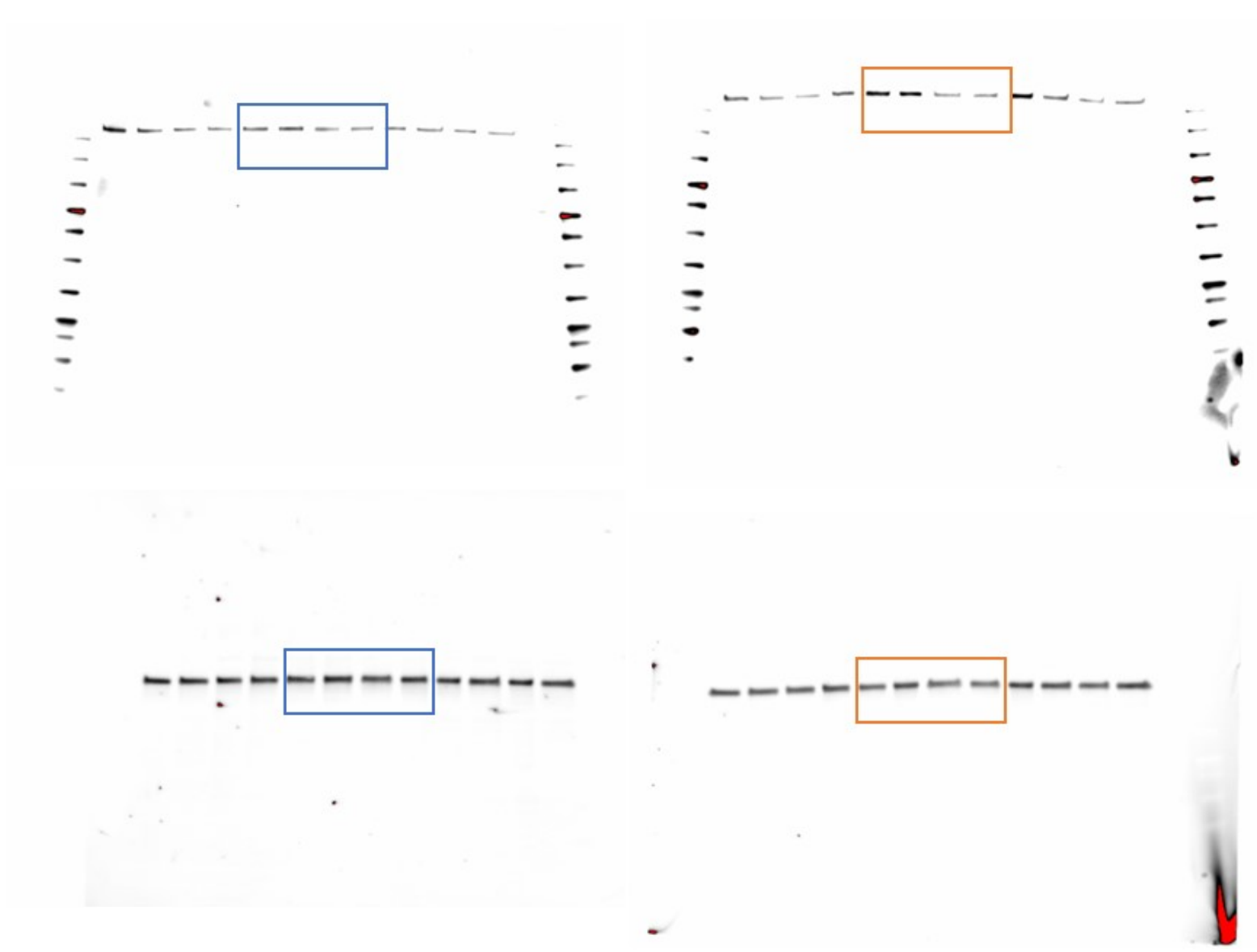

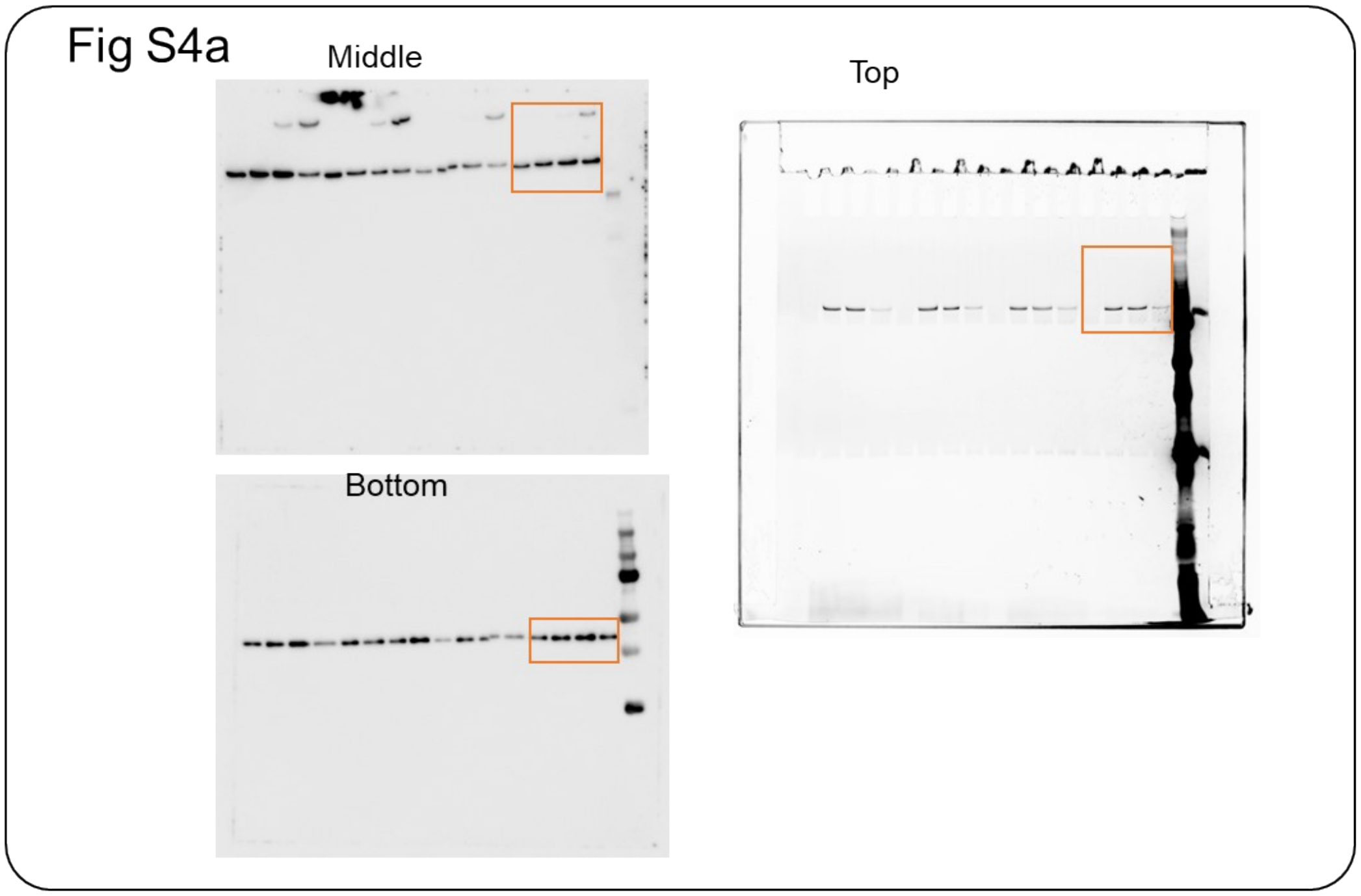
Fig S4a
Middle
Top
Bottom

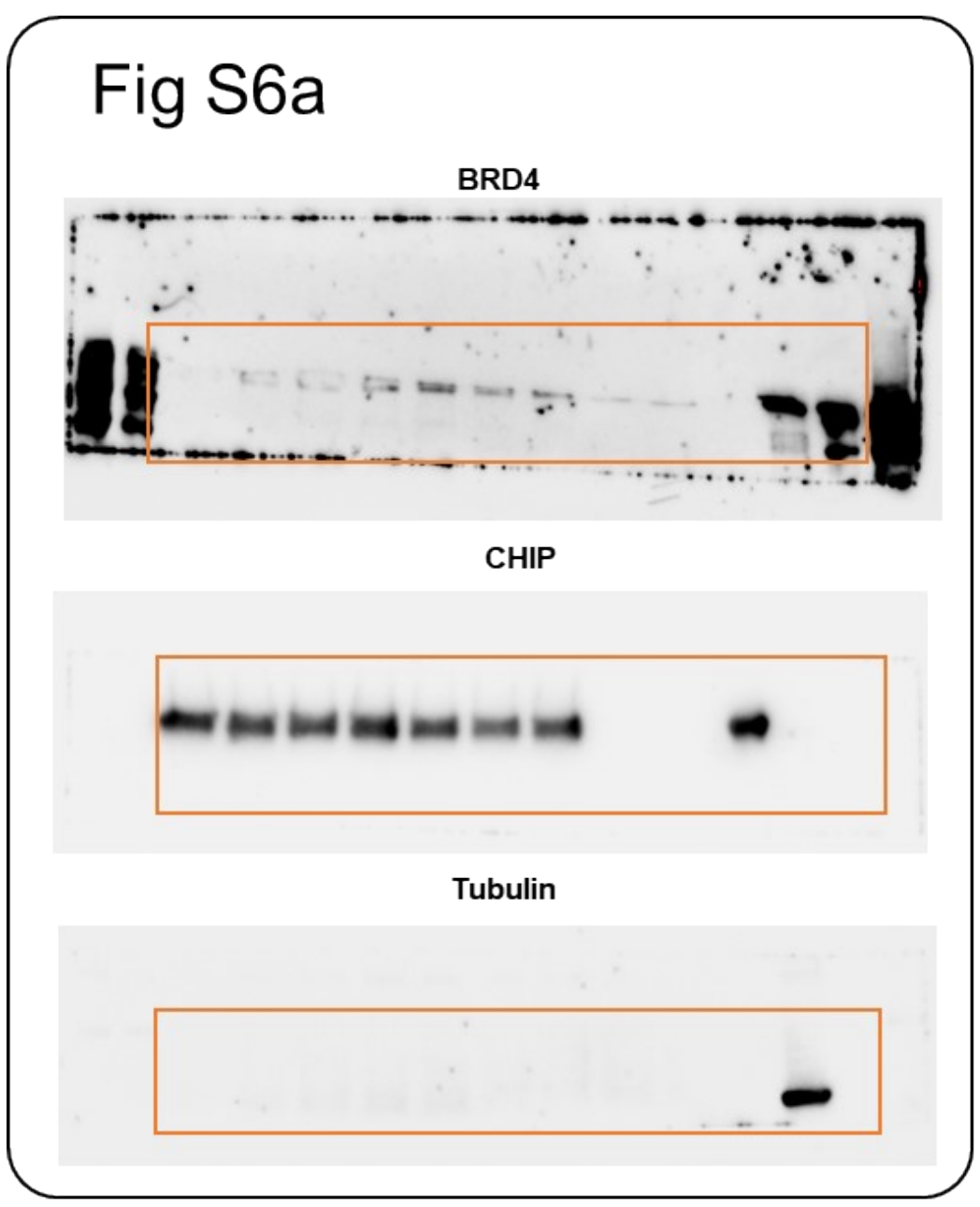
Fig S6a
BRD4
CHIP
Tubulin

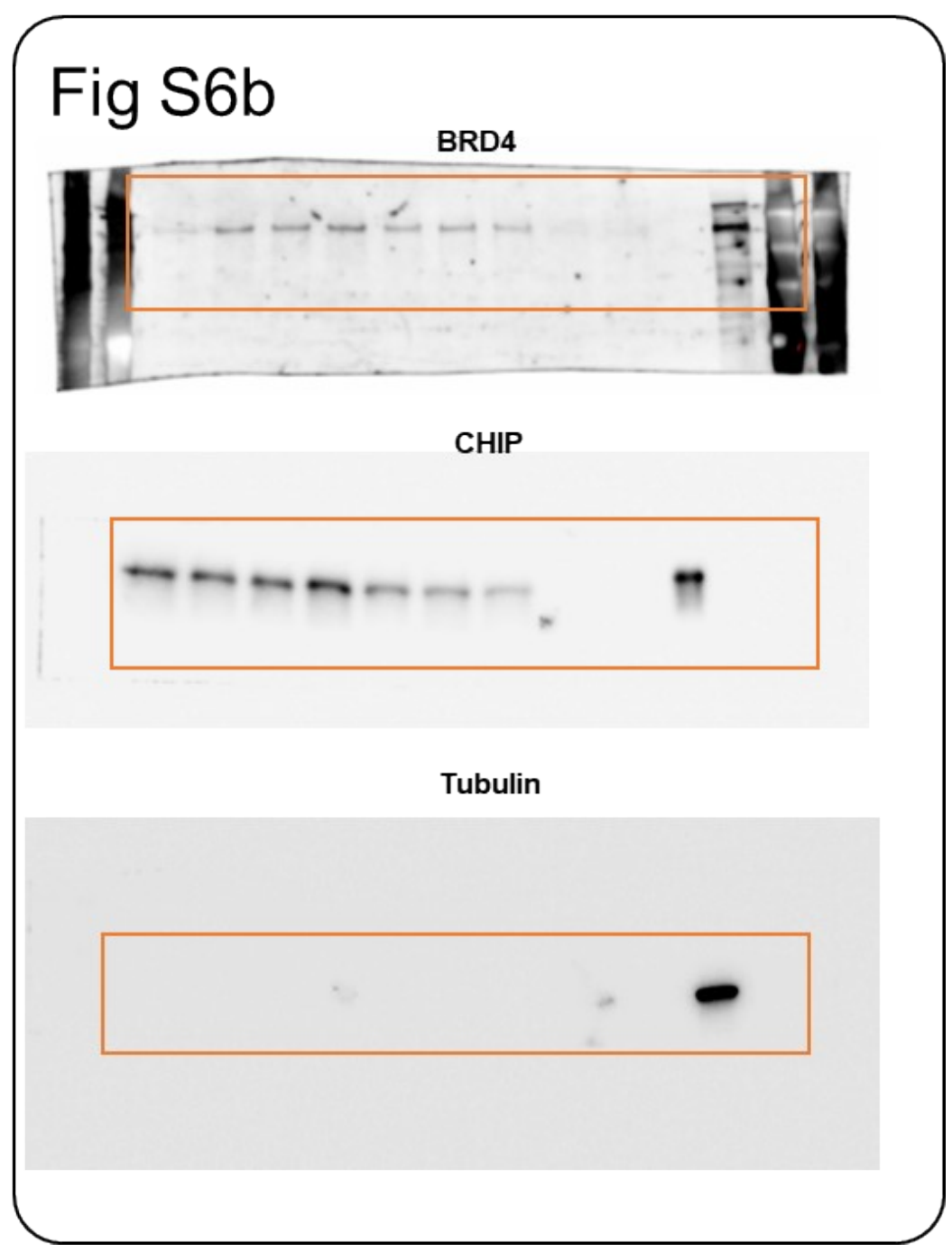
Fig S6b
BRD4
CHIP
Tubulin

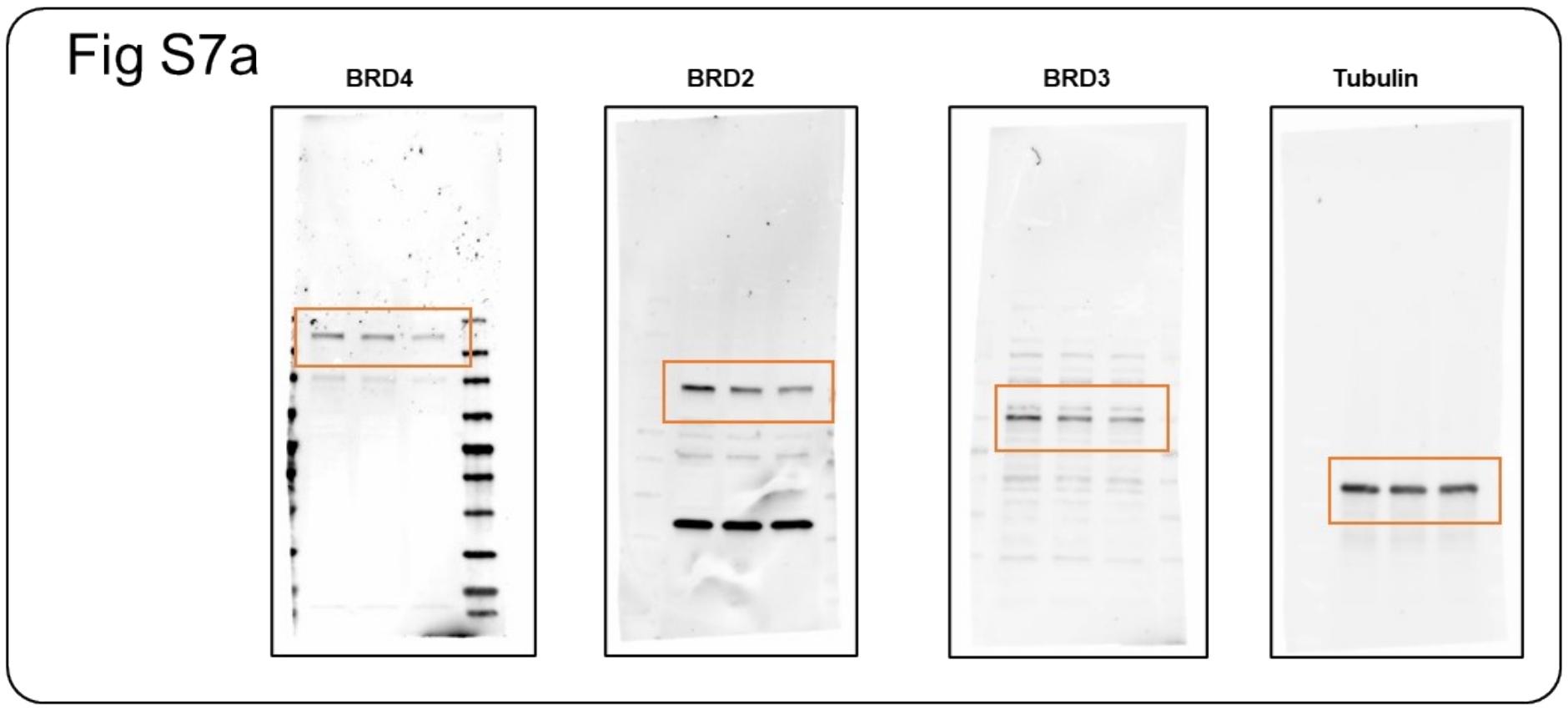

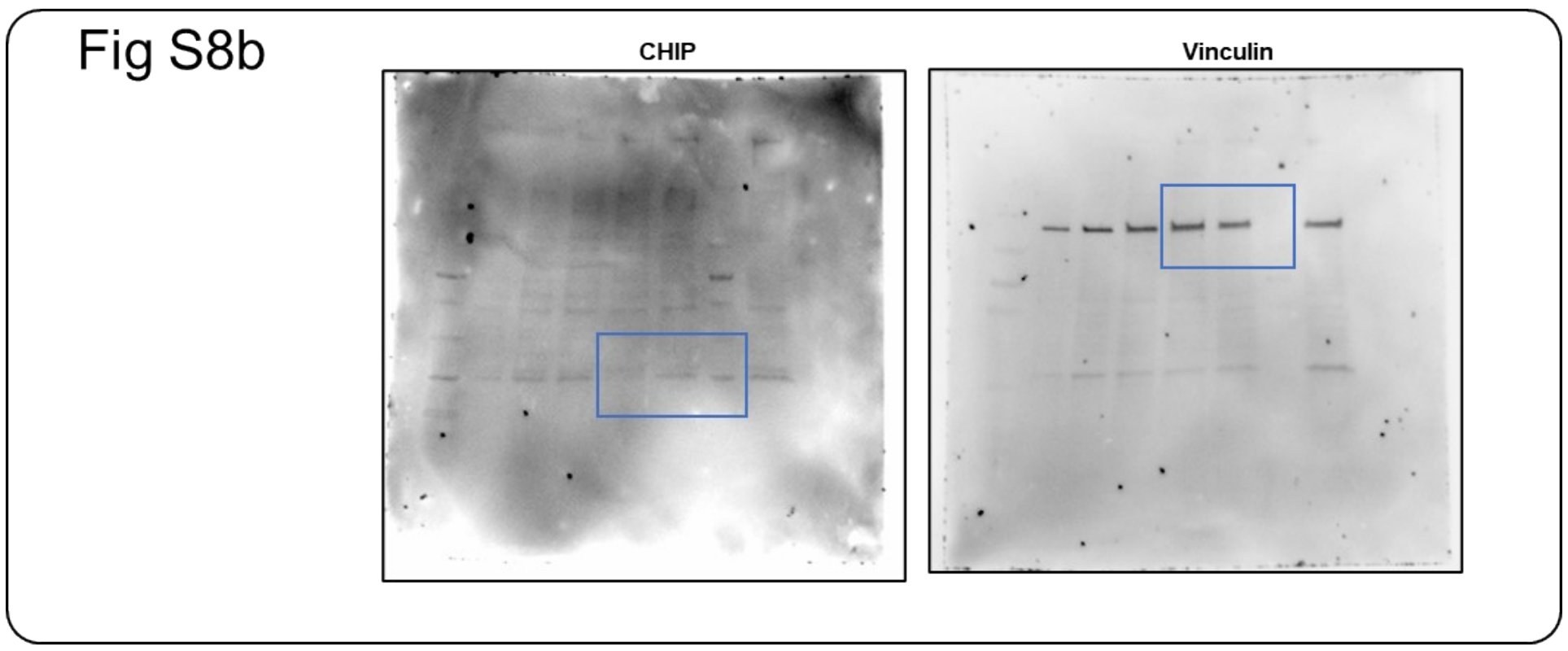

210

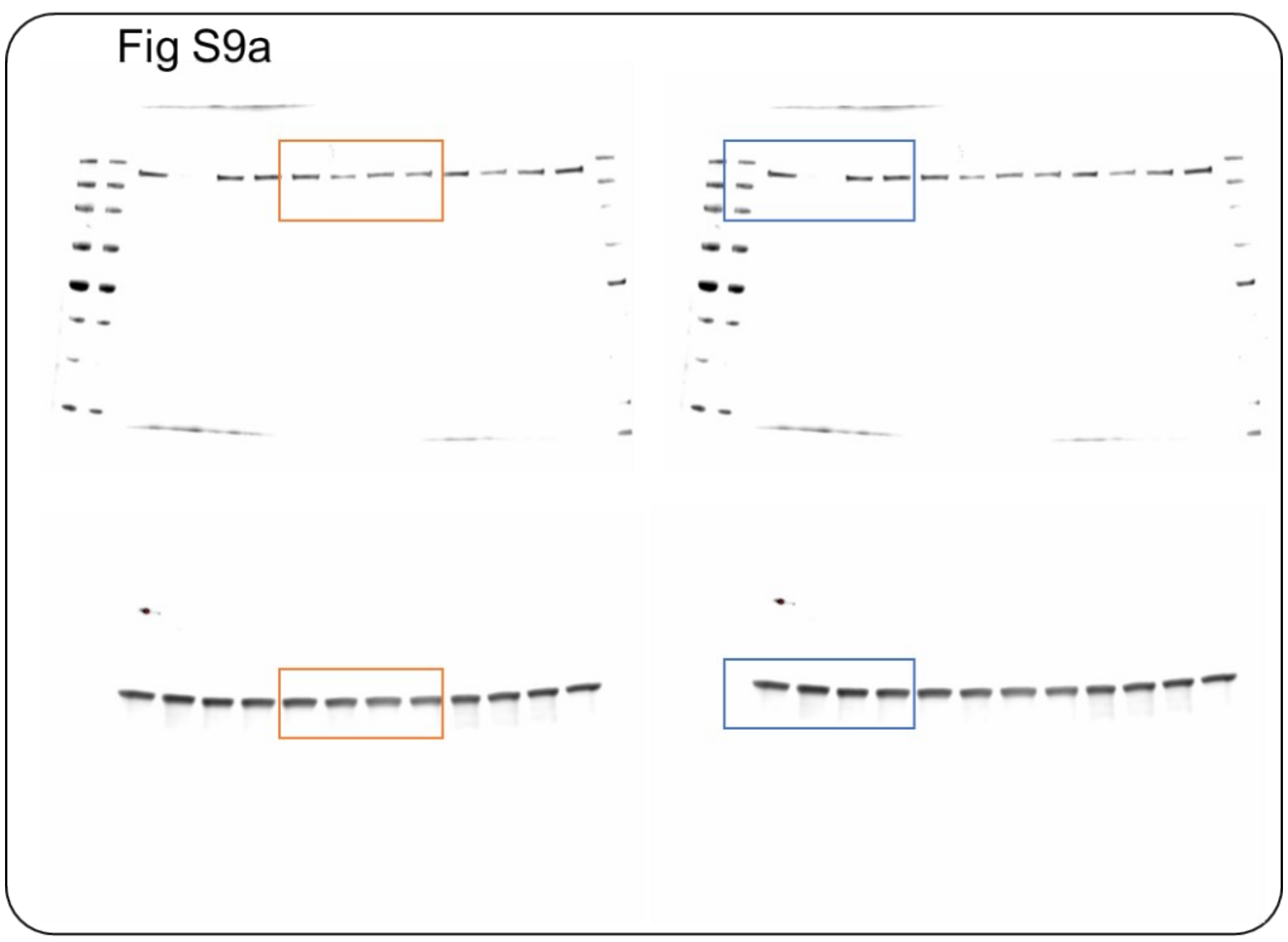

211
